# Manganese catalysed dehydrogenative synthesis of polyureas from diformamide and diamines†

**DOI:** 10.1039/d3cy00284e

**Published:** 2023-05-15

**Authors:** Angus McLuskie, Claire N. Brodie, Michele Tricarico, Chang Gao, Gavin Peters, Aaron B. Naden, C. Logan Mackay, Jin-Chong Tan, Amit Kumar

**Affiliations:** a School of Chemistry, University of St. Andrews North Haugh St. Andrews KY169ST UK ak336@st-andrews.ac.uk; b Department of Engineering Science, University of Oxford Parks Road Oxford OX13PJ UK jin-chong.tan@eng.ox.ac.uk; c School of Chemistry, University of Edinburgh EH93FJ UK

## Abstract

We report here the synthesis of polyureas from the dehydrogenative coupling of diamines and diformamides. The reaction is catalysed by a manganese pincer complex and releases H_2_ gas as the only by-product making the process atom-economic and sustainable. The reported method is greener in comparison to the current state-of-the-art production routes that involve diisocyanate and phosgene feedstock. We also report here the physical, morphological, and mechanical properties of synthesized polyureas. Based on our mechanistic studies, we suggest that the reaction proceeds *via* isocyanate intermediates formed by the manganese catalysed dehydrogenation of formamides.

## Abstract 2

Safer feedstock. Base-metal based catalyst. Mechanistic studies. Chemical, physical, morphological, and mechanical properties of polyureas.

## Graphical abstract

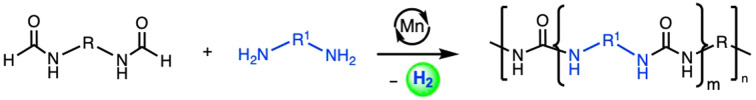

## Introduction

Polyureas are useful plastics with applications in the construction, coating, and biomedical industries.^1–5^ Their global market is £780 million per year and is expected to grow to £1.4 billion by 2030.^6^ Polyureas are industrially made from the reaction of diamines with diisocyanates; diisocyanates are made from phosgene gas which is extremely toxic and hazardous to human health and the environment. Thus, there is an urgent need to develop greener methods that avoid toxic feedstock for the synthesis of polyureas.

Indeed, a few methods that avoid diisocyanates feedstock have been reported for the synthesis of polyureas. For example, polyureas can be made from the condensation of diamines with CO_2_, however, either a dehydrating agent or a harsh reaction condition (*e.g.* 100 bar and 180 °C) is needed for this process.^7–9^ The use of other carbonylating agents such as urea,^10^ carbamate^11^ or biscarbamate^12^ instead of diisocyanates has also been demonstrated for the synthesis of polyureas.^13^ However, they suffer from the issues of limited substrate scope, low selectivity, or the use of harsh reaction conditions or specialty solvents.

Catalytic dehydrogenation is a green and atom-economic approach to the synthesis of organic compounds.^14,15^ The synthesis of urea derivatives has been reported from the dehydrogenative coupling of amines and methanol using ruthenium and iron pincer catalysts by Hong^16^ and Bernskoetter,^17^ respectively. Milstein has reported the synthesis of urea derivatives by the dehydrogenative coupling of formamide with amines in the presence of a ruthenium pincer catalyst.^18^ Gunanathan has also reported the synthesis of urea derivatives from the coupling of *N*,*N*′-dimethylformamide and amine where dimethylamine was observed as a by-product in the presence of a ruthenium-pincer catalyst.^19^

We have recently expanded the concept of the dehydrogenative synthesis of urea derivatives and reported a new method for the synthesis of polyureas from the dehydrogenative coupling of diamines and methanol using ruthenium^20^ and manganese^21^ pincer complexes. Liu has also reported the synthesis of polyureas from the dehydrogenative coupling of diamines and methanol using analogous manganese pincer complexes.^22^ The discovered method substitutes diisocyanates with methanol which is relatively much less toxic. Additionally, methanol is cheaper and can be made from renewable feedstock which makes the process potentially more sustainable.^23^ However, this method has two limitations: (a) molecular weight issue: the need for high temperature (150 °C) makes it difficult to use methanol under open conditions due to its low boiling point (65 °C) because of which reactions were conducted in a sealed system. This limits the polymer chain length or their molecular weight due to the accumulation of H_2_ gas in the system which can disfavour the dehydrogenation reaction. (b) Functionality issue: only one type of functionality (*e.g.* either aliphatic or aromatic) can be incorporated in the polyurea using this method whereas the conventional methodology can make polyurea containing two different functionalities – one coming from diisocyanate and the other from diamine. This is particularly useful for various applications where a rigid/hard segment (from aromatic groups) and a soft/flexible segment (from aliphatic chains) are needed in the polymer. The presence of both hard and soft segments in polyureas leads to a unique microphase separation in the polymer microstructure that leads to desired macroscopic properties, such as stability, high strength, and aging resistance.^4,24^

**Fig. 1:**
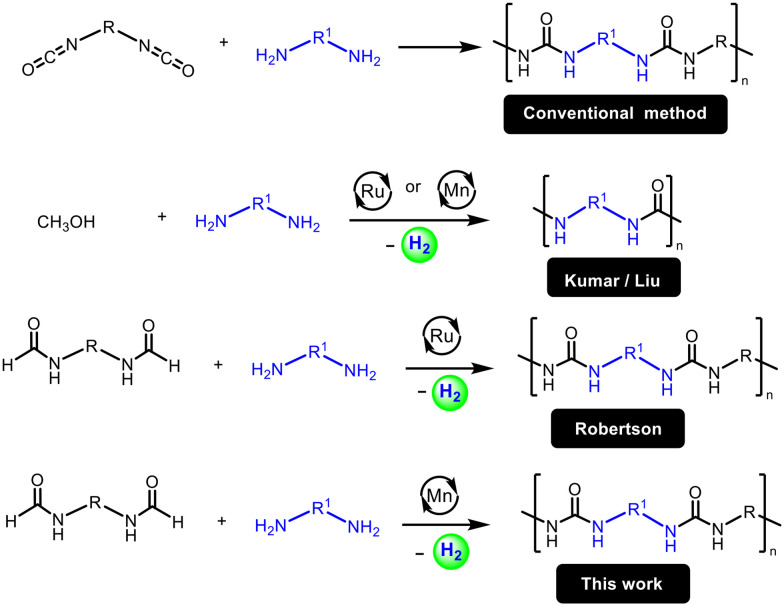
Synthesis of polyureas using conventional and dehydrogenative routes.

An alternative approach to avoid diisocyanates as well as overcome these limitations is to make polyureas from the dehydrogenative coupling of diformamides and diamines. This approach has been recently demonstrated by Robertson using a ruthenium pincer catalyst (Fig. 1).^25^ However, the use of precious metals such as ruthenium raises concerns of sustainability due to their high cost and low abundance on the earth's crust. The use of a catalyst based on an earth-abundant metal such as manganese, which is the third most abundant transition metal on earth's crust, can make the process more cost-effective and sustainable.^26^ Indeed, several (de)hydrogenative transformations catalysed by manganese complexes have been reported in the recent past.^27–30^ We report here that a manganese pincer complex Mn(PN^H^P-iPr)(CO)_2_Br, 1, (PN^H^P-iPr = *N*,*N*′-bis(diisopropylphosphinoethyl)amine) is an effective precatalyst for the dehydrogenative coupling of diformamides and diamines to form polyureas (Fig. 1). We also present their chemical (spectroscopy, mass spectrometry), physical (melting temperatures, decomposition temperatures), morphological (SEM) and mechanical (indentation modulus, hardness) properties of the synthesized polyureas.

## Results and discussion

**Fig. 2:**
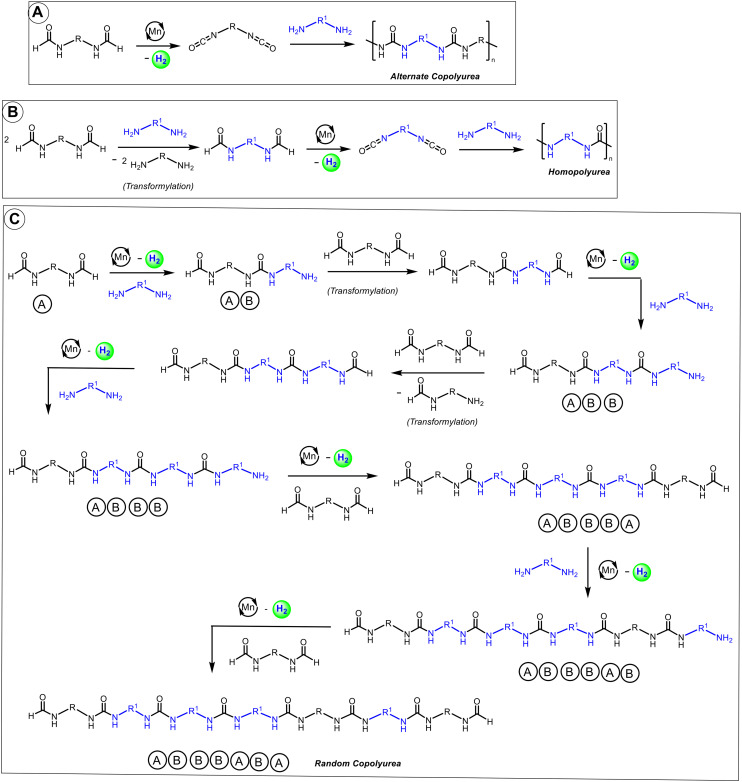
Proposed pathway for the formation of alternate copolyurea (A), homopolyurea (B) and random copolyurea (C) from the manganese catalysed dehydrogenative coupling of diformamides and diamines.

We started our investigation by studying the reaction of *N*,*N*′-(1,4-phenylene)diformamide (DF1, 0.5 mmol) with diaminooctane (DA1, 0.5 mmol) in the presence of a manganese pincer complex 1 (2 mol%) and KO^*t*^Bu (8 mol%) in THF solvent in a sealed system. Performing the reaction at 150 °C, in THF under sealed conditions for 24 h led to the isolation of an off-white solid in 82% yield (Table 1, entry 1). The product did not dissolve in common solvents such as water, THF, toluene, DCM, and CHCl_3_. It exhibited partial solubility in TFA (trifluoroacetic acid), therefore *d*-TFA was used for the NMR analysis. ^1^H and ^13^C{^1^H} NMR spectra showed signals corresponding to both aliphatic and aromatic protons that would come from (aliphatic) diamines and (aromatic) diformamides. The end group analysis of the partially soluble sample showed the number average molecular weight (*M*_n_) to be 1657 Da at the end of a 24 h reaction period. In the case of Robertson's report on the ruthenium catalysed synthesis of polyureas from diformamides and diamines, the molecular weight (*M*_n_) of a polyurea was found to significantly increase after 1 day of reaction time (*e.g.* 3700 Da after day 1; 13 300 Da after day 2; and 32 800 Da after day 3) in a manner expected for a step growth polymerisation mechanism.^25^ However, in our case, the formation of insoluble/poorly soluble material at the end of polymerisation reaction has made the estimation of molecular weight difficult. The IR spectrum of the polyurea (entry 1) showed a signal at 1680 cm^−1^ corresponding to the carbonyl stretching frequency and 1576 cm^−1^ corresponding to N–H bending both characteristics to a urea functional group. Interesting insights were obtained from MALDI FT-ICR mass spectrometry studies that showed the presence of repeating units corresponding to polyurea formed from aromatic diformamide (DF1) and aliphatic diamine (DA1). Mass spectrometry data also showed that the formed polymers contained all combinations of end groups: amine, amine; formyl, formyl; and formyl, amine. More interestingly, the mass spectrometry data revealed that the formed material is a mixture of copolyurea (formed from DF1 + DA1) as well as homopolyurea (resulting only from DA1). We speculate that the formation of the latter occurs *via* decarbonylation (*vide infra*) or/and the transformylation of DA1 with DF1 with the elimination of *p*-phenylenediamine (Fig. 2). Such transformylation reaction has been reported by us and others in the past.^21,31,32^ Considering the insoluble nature of the majority of the isolated material it was not possible to determine the exact ratio of homopolyurea and copolyurea. However, for an approximate comparison in different reaction conditions, we have provided the ratio of the integration of aliphatic *vs.* aromatic signals from the ^1^H NMR spectra (Table 1, see ESI† Fig. S1). These need to be used with caution as the isolated materials are only partially soluble in *d*-TFA. TGA (thermogravimetric analysis) and DSC (differential scanning calorimetry) studies were conducted to estimate the decomposition temperature (*T*_d_) and melting temperature (*T*_m_) of the polyurea which were found to be 246 °C and 221 °C respectively in this case.

**Table 1:** Catalytic conditions for the synthesis of polyureas from diformamides and diamines^a^

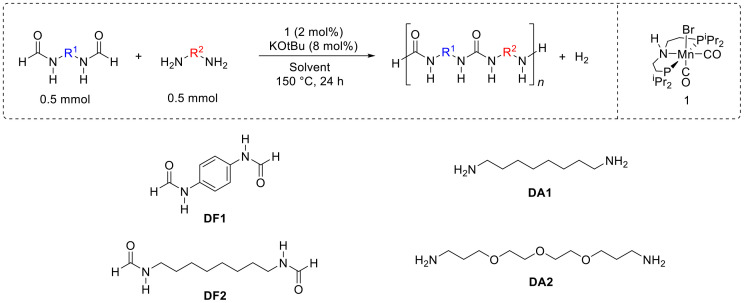										
Entry	Diformamide	Diamine	Base	Solvent	N_2_	Yield/%	*M*_n_ (MALDI)	Al : Ar	*T*_d_/°C	*T*_m_/°C
1	DF1	DA1	KO^*t*^Bu	THF	Sealed	82	1877	1.67	246	221
2	DF1	DA1	KO^*t*^Bu	Anisole	Sealed	77	2111	1.03	246	220
3	DF1	DA1	KO^*t*^Bu	Anisole	Open	32	2111	1.67	237	201
4	DF1	DA1	KO^*t*^Bu	Diglyme	Open	79	1904	2.01	238	212
5	DF1	DA1	KO^*t*^Bu	DMSO	Open	44	—	4.88	266	211
6	DF1	DA1	K_2_CO_3_	Anisole	Open	30	1770	1.73	242	204
7	DF2	DA1	KO^*t*^Bu	Anisole	Open	85	2111	N/A	253	216
8	DF2	DA1	KO^*t*^Bu	Diglyme	Open	25	2110	N/A	213	210
9	DF2	DA1	KO^*t*^Bu	THF	Open	78	2594	N/A	247	215
10	DF1	DA2	KO^*t*^Bu	Anisole	Open	94	3140^b^	1.16	223	—

a Catalytic conditions: diamine (0.5 mmol), diformamide (0.5 mmol), solvent (2 mL), complex 1 (2 mol%), and KO^*t*^Bu (8 mol%). Al : Ar = aliphatic : aromatic NMR integral relationship. *M*_n_ (MALDI, g mol^−1^): value is estimated as the maximum signal observed in MALDI FT-ICR mass spectrometry. *T*_d_ (decomposition temperature) was recorded at 5% mass loss. *T*_m_ stands for melting temperature.

b *M*_n_ of 44 000 g mol^−1^ was estimated using GPC.

We also studied the effect of solvents on the polymerisation reaction as a high boiling point solvent can allow the reaction to occur under open conditions (flow of N_2_ or Ar) and at high temperature, and the polarity of solvent can influence the solubility of oligomers. Both these factors can affect the polymer chain length. Changing the solvent to anisole keeping the remaining conditions the same led to the observation of similar composition of polymers, albeit at a slightly reduced yield (77%, Table 1, entry 2) with respect to that in the case of THF (entry 1). Performing the reaction in an open system using anisole as a solvent resulted in a drop in yield (32%; entry 3). However, this issue is overcome when the solvent is changed to diglyme and the catalysis is carried out in open conditions under the flow of N_2_ (entry 4), the product can be isolated in 79% yield. MALDI FT-ICR mass spectrometry showed a higher abundance of signals corresponding to copolyurea than those to homopolyurea from the transformylation reaction (see ESI,† Table S2). Performing the reaction in DMSO (entry 5), results in a significant reduction in yield (44%). An experiment carried out in anisole with a change in base to K_2_CO_3_ led to a low yield of 30% (entry 6). Additionally, conducting control experiments in the absence of either a base or complex 1 (remaining conditions as per described in Table 1, entry 1) did not lead to the isolation of any product and starting materials were recovered at the end of the reaction. Attempting to synthesise polyurea from DF1 with a secondary amine, piperazine, was also unsuccessful (see ESI,† Table S1, entry 15).

We next studied the coupling of diamine DA1 with another diformamide DF2 (*N*,*N*′-(octane-1,8-diyl)diformamide). The “R” groups of DF2 and DA1 are identical, so, regardless of transformylation, a reaction of these together would generate polymers of the same chemical structure, and identical to the polyurea made from the dehydrogenative coupling of 1,8-diaminooctane and methanol as previously reported by us using a manganese pincer complex 1.^20^ The catalytic studies were conducted in anisole, diglyme, and THF under the open flow of nitrogen. Anisole and THF led to higher yields (85% and 78%) whereas lower yields were obtained in the case of diglyme as described in Table 1 (entries 7–9). *T*_d_ (253 °C) and *T*_m_ (216 °C) in the case of polyurea made from DF2 and DA1 (entry 7) were found to be higher than that of polyurea made from DA1 and methanol (*T*_d_, 190 °C and *T*_m_, 89 °C) under the reaction condition: complex 1 (2 mol%), KO^*t*^Bu (8 mol%), 150 °C, 24 h THF.^21^

To resolve the difficulty in determining polyurea molecular weight because of poor solubility (*vide supra*), we have also performed the polymerisation of DF1 with a diamine which has a glycol-rich backbone, DA2. To our satisfaction, this reaction yielded polyurea in high yield (94%) that is water-soluble. While MALDI-MS returned the highest molecular weight fragment at *m*/*z* 3140 g mol^−1^, end-group analysis of the ^1^H NMR spectrum of this soluble sample in D_2_O estimates *M*_n_ to be ∼19 000 g mol^−1^. Furthermore, GPC analysis of this sample (H_2_O eluant, relative to monodisperse PEG/PEO standards) estimates a higher molecular weight still, *M*_n_ 44 000 g mol^−1^, with polydispersity, *Đ* = 1.4. Despite narrow dispersity measured, the GPC curve obtained for this sample does exhibit bimodality (see Fig. S86†), possibly due to the presence of both alternate copolyureas and random copolyureas, as suggested by MALDI-MS. Taking the molecular weight information obtained from end-group analysis and GPC for this soluble polymer, it is likely, therefore, that the molecular weights obtained from MALDI-MS for other polyurea samples here are underestimated. Indeed, observation of polymers with molecular weight >5000 g mol^−1^ is known to be difficult by MALDI-MS.^33,34^ We also studied the morphology of polyureas made from DF1 and DA1 (containing both aromatic and aliphatic groups, Table 1, entry 4) as well as from DF2 and DA1 (containing only aliphatic groups, Table 1, entry 7). The data obtained from the scanning electron microscope showed that the aliphatic polyurea (from DF2 and DA1, Table 1, entry 7) is composed of small sheet-like features typically ∼1–3 μm long and ∼50–70 nm thick whereas the aromatic polyurea (from DF1 and DA1; Table 1, entry 7) exhibits a more porous and less densely packed microstructure within the larger agglomerations (see ESI,† Fig. S83). Energy dispersive X-ray spectroscopy (EDX) provides a convenient means of rapidly assessing the elemental composition of the samples. The EDX spectra for both samples confirmed the presence of C, N, and O.

To get an understanding of the mechanical properties of polyureas made from this method, we performed nanoindentation analysis of both aromatic (from DF1 and DA1; Table 1, entry 4) as well as aliphatic polyurea (from DF2 and DA1; Table 1, entry 7). The tests were carried out using a KLA iMicro nanoindenter, equipped with a 50 mN force actuator and a Berkovich tip. Continuous stiffness measurements (CSM) were performed, allowing to measure the indentation modulus E* (E* = *E*/1 − *ν*^2^) which can be used in place of Young's modulus *E* when the Poisson's ratio *ν* is unknown. Our studies showed a high indentation modulus of 4.25 ± 0.72 GPa and a hardness of 252 ± 64 MPa for aromatic polyurea (from DF1 and DA1, entry 4) as well as for the aliphatic polyurea (indentation modulus: 4.93 ± 0.63 GPa; hardness: 291 ± 59 MPa made from DF2 and DA1, entry 7, see ESI,† Section S12). The elastic modulus of a commercial polyurea XS-350 has been reported to be less than what we observe here (100 MPa).^35^

**Fig. 3:**
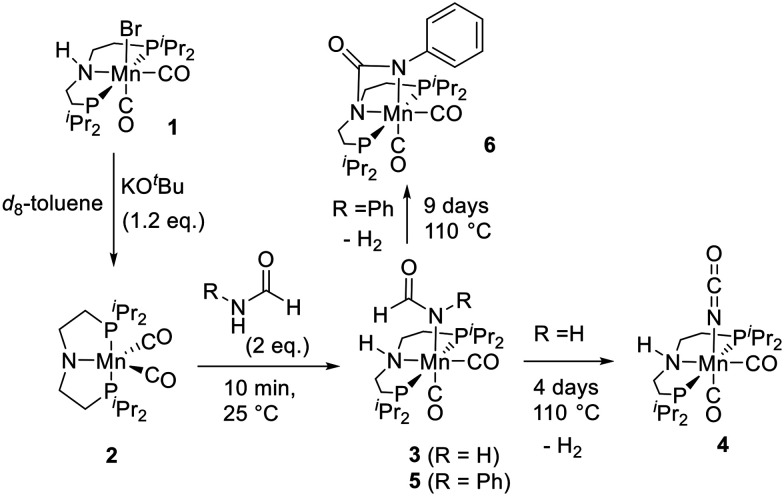
Reaction of formamide with *in situ* generated complex 2.

In our recent work on the manganese catalysed synthesis of urea derivatives from formamides and amines, we reported a mechanism based on the DFT computation where the manganese complex 2 dehydrogenates a formamide to form an isocyanate followed by its subsequent reaction with an amine to form a urea derivative.^21^ We speculate that the synthesis of polyureas from diformamides and diamines as reported here proceeds *via* a similar pathway. To probe further, we monitored the reaction under stoichiometric and catalytic conditions using NMR spectroscopy and mass spectrometry. The reaction of complex 1 with KO^*t*^Bu (1.2 equivalents) in an NMR tube in toluene-*d*_8_ resulted in the formation of the amido-complex 2 as also previously reported (Fig. 3).^36^ Addition of 2 equivalents of formamide (HCONH_2_) or formanilide (PhCONH_2_) to the *in situ* formed complex 2 resulted in the formation of a new complex as evidenced by the complete consumption of the signal corresponding to 2 (*δ*_P_ 113.0 ppm) in 10 minutes (room temperature) and the concomitant appearance of a new signal in the ^31^P{^1^H} NMR spectra. Analysis of the ^1^H and ^13^C{^1^H} NMR spectra as well as HRMS (EI) data suggests the formation of complexes 3 and 5 through N–H activation of formamide and formanilide *via* metal–ligand cooperation.^37^ The N–H activation of formanilide by complex 2 to form complex 5*via* metal–ligand cooperation has been recently reported by Boncella and Tondreau.^38^ Interestingly, heating the NMR tube containing the *in situ* formed complex 3 at 110 °C for 4 days led to the quantitative formation of a new complex (^31^P{^1^H} NMR: *δ*_P_ 88.6 ppm) which was characterised as the manganese-isocyanate complex 4 using NMR spectroscopy and mass spectrometry. The ^1^H NMR spectrum also shows a signal at *δ*_H_ 4.51 corresponding to dissolved H_2_ that would be produced during the formation of isocyanate upon dehydrogenation of formamide. These results agree with a recent report by Milstein where a ruthenium-PNP pincer complex was found to activate and dehydrogenate formamide to form a ruthenium-coordinated isocyanate complex, analogous to 4.^18^ Heating complex 5 at 110 °C slowly formed a new complex as observed by a clean signal in ^31^P{^1^H} NMR spectrum at *δ*_P_ 89.2 ppm after 9 days. NMR spectroscopy and HRMS data suggested the formation of complex 6 featuring the Mn–N–(C=O)–N cycle that would be formed from the dehydrogenation of complex 5. Indeed, dissolved H_2_ gas was observed in the ^1^H NMR spectrum (*δ*_H_ 4.51) of the reaction mixture. The ^13^C{^1^H} NMR chemical shift of the carbonyl carbon in complex 6 shifts further upfield (*δ*_C_ 156.8) in comparison to that of complex 5 (*δ*_C_ 171.2) as would be expected upon decreasing electron density of the carbonyl carbon on becoming a urea moiety. Additionally, a ^1^H,^13^C-HMBC (toluene-*d*_8_) NMR spectrum of complex 6 shows the correlation between the carbonyl carbon (*δ*_C_ 156.8 ppm) and the C–H protons of the ligand backbone (*δ*_H_ 2.22–2.12 ppm) supporting the proposed structure of complex 6 (Fig. S100, see ESI†). This is similar to a recent report by Milstein where an analogous ruthenium-pincer complex activates the formanilide and dehydrogenates to form a complex featuring Ru–N–(C=O)–C cycle.^18^ Analogous manganese complex featuring Mn–N–(C=O)–O cycle has also been recently reported by Milstein from the activation of CO_2_ across a manganese–amido bond.^39^ GC-MS analysis of the reaction mixture upon heating complex 5 (110 °C, 9 days) showed the formation of aniline and diphenylurea. We speculate that under this condition formanilide can undergo decarbonylation to form aniline which could then react with isocyanate to form diphenylurea.^19,40^ This could be an alternative pathway to the formation of homopolyurea in addition to the transformylation route proposed in Fig. 2. Conducting a catalytic reaction of diformamide (DF1, 0.5 mmol) in the absence of diamine (remaining conditions described as per Table 1 entry 7), resulted in the formation of homopolyurea in ∼5% yield. It is possible that both pathways (transformylation and decarbonylation) occur under the catalytic conditions. Furthermore, the formation of complexes from the N–H activation of diformamide and the corresponding dehydrogenation product analogous to complexes 5 and 6 were also obtained in catalyst speciation studies when we measured the NMR spectra for a sample taken after 1 h from the catalytic experiment as described in Table 1, entry 3 (see ESI,† Fig. S103). These observations support our hypothesis that the manganese complex 2 is capable of dehydrogenating formamide and forming an isocyanate intermediate that can further react with an amine to form a urea functional group. Our efforts to grow crystals suitable for single-crystal X-ray diffraction for complexes 4 and 6 were not successful. Interestingly, the reaction of complex 6 with aniline (toluene, 110 deg, 24 h) did not lead to the formation of any urea derivative suggestive of complex 6 to be an off-cycle intermediate. This is consistent with a recent report by Sanford and co-workers (that was published during the communication of this manuscript)^41^ where the reaction of an analogue of complex 6 with cyclohexanol doesn't yield any carbamate derivative. They have suggested that the O-coordinated isocyanate rather than the N-coordinated one (like in complex 6) is the active species which might also be possible in our case.

## Conclusions

In conclusion, we report here the synthesis of polyureas from the manganese catalysed dehydrogenative coupling of diformamides with diamines. Polyureas have been chemically characterized by NMR and IR spectroscopies as well as MALDI FT-ICR mass spectrometry. Further insights into the physical properties (decomposition temperature and melting temperature), morphology (SEM, EDX), and mechanical properties (indentation modulus, and hardness using nanoindentation) have also been provided. Based on our experimental studies reported here (Fig. 3) and previous report by us using DFT computation,^21^ we propose that the polymerization reaction proceeds by the dehydrogenation of diformamides to form diisocyanates followed by their reaction with diamines to form polyureas. Additionally, our studies also suggest the possibility of competitive pathways based on transformylation (Fig. 2) and decarbonylation (*vide supra*) that would lead to the formation of homopolyurea or random copolyurea as observed by the MALDI FT-ICR mass spectrometry in this case. The presented methodology replaces the use of diisocyanates (which can be toxic) with a safer feedstock – diformamides. The use of an earth-abundant metal-based catalyst is an added advantage that makes the process more sustainable.

## Data availability

The raw research data supporting this publication can be accessed at the University of St Andrews Research Portal: https://doi.org/10.17630/36c115ac-4dbb-4a87-a3e9-c042f8f1a91e.

## Conflicts of interest

There are no conflicts to declare.

## Supplementary Material

CY-013-D3CY00284E-s001
