# Supplementary material for: Manganese catalysed dehydrogenative synthesis of polyureas from diformamide and diamines
Source: Catal Sci Technol. 2023 May 15;13(12):3551–7. doi: 10.1039/d3cy00284e (PMC10278093; doi:10.1039/d3cy00284e)
Supplement: CY-013-D3CY00284E-s001 [file CY-013-D3CY00284E-s001.pdf]

Electronic Supplementary Material (ESI) for Catalysis Science & Technology.
This journal is © The Royal Society of Chemistry 2023

Supporting Information for Manuscript:

# Manganese Catalysed Dehydrogenative Synthesis of Polyureas from Diformamides and Diamines.

Angus McLuskie,[a†] Claire N Brodie,[a†] Michele Tricarico,[b] Chang Gao,[a] Gavin Peters,[a] Aaron B Naden,[a] C. Logan Mackay,[c] Jin-Chong Tan,*[b] Amit Kumar[a]*

[a] *School of Chemistry, University of St. Andrews, North Haugh, St. Andrews, KY169ST, UK.*
[b] *Department of Engineering Science, University of Oxford, Parks road, Oxford. OX13PJ, UK.*
[c] *School of Chemistry, University of Edinburgh, EH93FJ.*
*†These authors contributed equally.*

* Amit Kumar (ak336@st-andrews.ac.uk)
* Jin-Chong Tan (jin-chong.tan@eng.ox.ac.uk)

## Table of Contents


1. General information ....................S3
2. General procedure for the synthesis of diformamide ....................S5
3. General procedure for the synthesis of polyureas from diformamides and diamines ....................S6
4. Solubility of polyureas ....................S7
5. Characterisation of polyureas....................S8
6. Spectral data of the isolated polyureas ....................S9
7. NMR spectra of the isolated polyureas ....................S14
8. IR spectra of the isolated polyureas ....................S36
9. Polymers observed via MALDI FT-ICR MS....................S44
10. Results of the MALDI-FT-ICR mass spectrometry measurements ....................S45
11. TGA and DSC data ....................S55
12. Mechanical characterisation of polyureas (Entries 4 and 7, Table S1) ....................S65
13. SEM and EDX studies. ....................S69
14. Mechanistic studies ....................S71
    14.1. Stochiometric reaction of Mn(PN$^{H}$P-iPr)$(CO)_2$Br (1) with formamides ....................S71
    14.2 NMR spectra of complex 3....................S76
    14.3 NMR spectra of complex 4....................S78
    14.4 NMR spectra of complex 5....................S81
    14.5 NMR spectra of complex 6....................S83
    14.6 NMR spectra from *in situ* speciation....................S86
    14.7. GC-MS selected chromatograms and mass spectra....................S87
    14.8 Mass Spectra....................S89
15. References ....................S91

## 1. General information

All experiments were carried out under inert atmosphere using standard Schlenk techniques unless specified. Complex **1**[1] and N,N'-(octane-1,8-diyl)diformamide[2] were prepared as described by methods reported in literature. Ethyl formate, N,N'-(1,4-phenylene)diformamide, and diamines were purchased from Sigma- Aldrich, Alfa Aesar, Strem, or TCI and used as received. THF and toluene were dried using a Grubbs type solvent purification system and degassed by Freeze-Pump-Thaw under nitrogen before use. Anhydrous anisole, diglyme and DMSO were purchased from Sigma-Aldrich and used as received. Deuterated solvents ($CDCl_3$, DMSO-$d_6$, *d*-TFA and $D_2O$) were purchased from Sigma-Aldrich and used as received. Toluene-$d_8$ was purchased from Sigma-Aldrich, dried over $CaH_2$, distilled and degassed by three successive freeze-pump-thaw cycles before use. Schlenk flasks of 250 mL were used for the synthesis of polyureas from diformamides and diamines.

For the preparation of the MALDI samples, polyureas were dissolved in neat TFA. 0.5 µL of the resulting solution was applied to a stainless steel MALDI target plate, 0.5 µL of the matrix was co-spotted and allowed to dry. Matrix was either 2,5-dihydroxybenzoic acid or alpha-cyano-4-hydroxycinnamic acid prepared at 10 mg/mL in 50:50 acetonitrile: 0.1% TFA. MALDI data was acquired using a 12T SolariX FT-ICR (Bruker Daltonics) equipped with a Nd:YAG 355 nm laser. The sample was acquired in positive MS mode between *m*/*z* 200 and *m*/*z* 5000.

Infrared spectra (ATR-FTIR) were recorded using a MIRacle™ from Pike or using a Shimadzu IRAffinity-1.

Thermal Gravimetric Analysis (TGA) was carried out using a Netzsch STA449C with a heating rate of 10 °C/min from 30 to 600 °C in an inert gas flow. Decomposition temperatures ($T_d$) were recorded by TGA at 5% weight loss. 5% weight loss was taken after solvent/water/residual reactants loss. DSC were collected using a Netzsch STA449C (10 °C/min from 30 to 600 °C under a flow of nitrogen gas at 25 mL/min) or using a Netzsch DSC204 –(10 °C/min between 80–600°C under a flow of nitrogen gas at 20 mL/min, after an initial heat/cool cycle [25–120 °C at 10 °C/min with a 20 minute isothermal at 120 °C] to remove the thermal history of the sample).

NMR spectra were recorded on a Bruker AVIII-HD 500 MHz NMR spectrometer at 298 K unless otherwise specified. Residual protio solvent was used as reference for $^1H$ spectra in deuterated solvent samples. Where *d*-TFA is used as an NMR solvent, samples were run with the presence of a $D_2O$ sealed capillary to aid locking when required. $^{31}P\{^1H\}$ NMR spectra were externally referenced to 85% $H_3PO_4$. All chemical

shifts (δ) are quoted in ppm and coupling constants (*J*) in Hz. NMR assignments were aided by 2D spectra ($^{1}H$,$^{1}H$-COSY, $^{1}H$,$^{13}C$-HSQC, $^{1}H$,$^{13}C$-HMBC) where required.

Scanning electron microscopy (SEM) was performed on an FEI Scios dualbeam FIB/SEM operated at an acceleration voltage of 3 kV and energy dispersive X-ray spectroscopy (EDX) was performed on a JEOL JSM-IT200 equipped with an embedded JEOL EDX detector and operated at 10 kV.

GC-MS spectra were collected as solutions in HPLC grade DCM using an Agilent 8860 GC system coupled to an Agilent 5977B EI instrument. EI spectra were collected as solutions in acetonitrile using a Micromass LCT spectrometer.

Gel permeation chromatography (GPC) was performed on an Agilent 1260 InfinityLab II GPC fitted with a refractive index (RI) detector (35 °C). The single (plus guard column) Agilent PolarGel column setup was contained within an oven (35 °C). $H_2O$ was used as the eluent at a flow rate of 1.0 mL $min^{-1}$. Samples were dissolved in the eluent (2.0 mg $mL^{-1}$), filtered (0.2 µm pore size) and run immediately. The calibration was conducted using a series of monodisperse poly(ethylene glycol) ($M_n$ = 194–20,000 g $mol^{-1}$) and poly(ethylene oxide) ($M_n$ = 30,000–55,000 g $mol^{-1}$) standards obtained from Agilent Technologies.

## 2. General procedure for the synthesis of diformamide

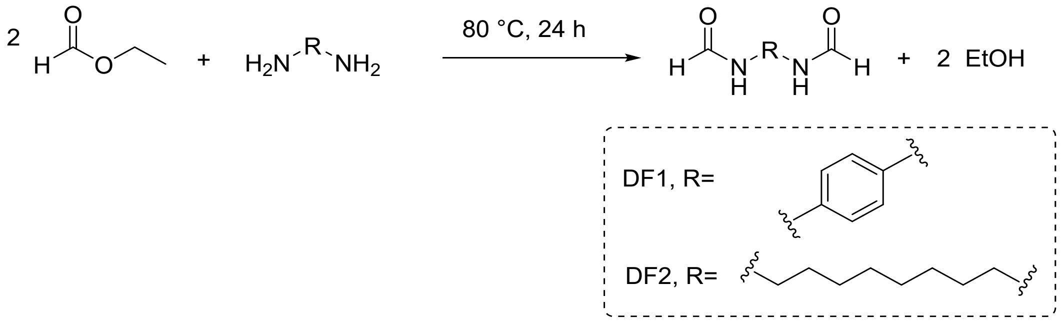


Ethyl formate (10 mL) and 1,8-diaminooctane (8.58 mmol, 1.44 g) were added to a round bottom flask and heated to reflux at 130 °C for 24 hours as per the method reported in the literature. The product mixture was left to cool before the excess ethyl formate was removed under vacuum to yield a white solid (1.72 g, 89%). The isolated product was characterized using $^{1}H$ and $^{13}C\{^{1}H\}$ NMR and IR spectroscopy. The spectral details correlate to literature results.[2]

## 3. General procedure for the synthesis of polyureas from diformamides and diamines

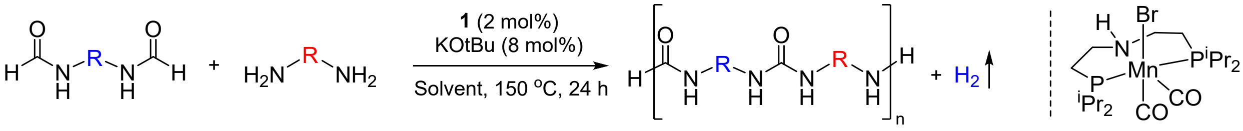


1

**1** (0.01 mmol, 2 mol%), diformamide (0.5 mmol), diamine (0.5 mmol) and $KO^tBu$ (0.04 mmol, 8 mol%) were added to a 250 mL J-Young's flask before solvent (2 mL) was added and the reaction heated to 150 °C. After 24 hours, the contents of the flask were cooled to room temperature, and the lid was slowly opened to release the gas pressure. *For reactions open under $N_2$ atmosphere, reactions were carried out in a Schlenk flask connected to Schlenk line open to $N_2$ supply and attached with a bubbler.* The reaction mixture (that could contain a solid) at the end of 24 h was transferred to a vial in which 5-10 mL of hexane was added. The vial was cooled to –30 ° C to aid precipitation of the product. The solid product was then collected by filtration, washed with hexane (3 × 10 mL) and then dried under vacuum.

The isolated product was characterised using $^1$H NMR, $^{13}$C{$^1$H} NMR and IR spectroscopies and MALDI-FT-ICR mass spectrometry.

**Note:** Caution must be taken as the reaction can produce up to ~96 mL of $H_2$ gas (298 K).

## 4. Solubility of polyureas

The solubility of a polyureas was tested by adding 10 mg of a polyurea in a vial containing 2 mL of a solvent ($CHCl_3$, THF, MeOH, acetone, $H_2O$, DMF, DMSO, TFA). All polyureas reported here were found to dissolve only partially in TFA, and not in other tested solvents, with the exception of that produced from DF-1 and DA-2. In the case of the polymer produced from DF-1 and DA-2, this polymer is soluble in water by design to allow GPC analysis.

## 5. Characterisation of polyureas

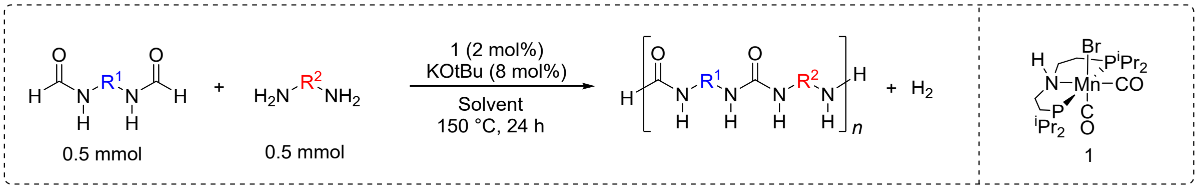


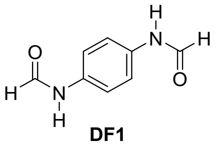
DF1

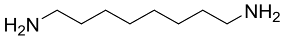
DA1

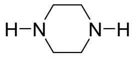
DA3

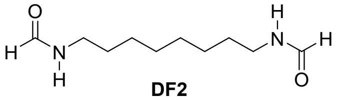
DF2

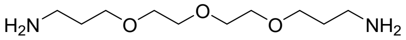
DA2

**Table S1** Reaction optimisation for the coupling of diformamide and diamine catalysed by **1**.[a]

| Entry | Diformamide | Diamine | Base | Solvent | $N_2$ | Yield /% | $M_n$ (MALDI) | Al:Ar | $T_d$ /°C | $T_m$ /°C |
|---|---|---|---|---|---|---|---|---|---|---|
| 1 | DF1 | DA1 | KO$^t$Bu | THF | Sealed | 82 | 1877 | 1.67 | 246 | 221 |
| 2 | DF1 | DA1 | KO$^t$Bu | Anisole | Sealed | 77 | 2111 | 1.03 | 246 | 220 |
| 3 | DF1 | DA1 | KO$^t$Bu | Anisole | Open | 32 | 2111 | 1.67 | 237 | 201 |
| 4 | DF1 | DA1 | KO$^t$Bu | Diglyme | Open | 79 | 1904 | 2.01 | 238 | 212 |
| 5 | DF1 | DA1 | KO$^t$Bu | DMSO | Open | 44 | - | 4.88 | 266 | 211 |
| 6 | DF1 | DA1 | $K_2CO_3$ | Anisole | Open | 30 | 1770 | 1.73 | 242 | 204 |
| 7 | DF2 | DA1 | KO$^t$Bu | Anisole | Open | 85 | 2111 | N/A | 253 | 216 |
| 8 | DF2 | DA1 | KO$^t$Bu | Diglyme | Open | 25 | 2110 | N/A | 213 | 207 |
| 9 | DF2 | DA1 | KO$^t$Bu | THF | Open | 78 | 2594 | N/A | 247 | 215 |
| 10 | DF2 | DA1 | KO$^t$Bu | DMSO | Open | 67 | - | N/A | 214 | 204 |
| 11 | DF2 | - | KO$^t$Bu | Anisole | Open | 2 | - | N/A | - | - |
| 12[b] | DF2 | DA1 | KO$^t$Bu | Anisole | Open | 29 | - | N/A | - | 220 |
| 13[c] | DF2 | DA1 | KO$^t$Bu | Anisole | Open | 10 | - | N/A | 231 | 219 |
| 14[d] | DF1 | DA2 | KO$^t$Bu | Anisole | Open | 94 | 3140 | 1.16 | 223 | - |
| 15 | DF1 | DA3 | KO$^t$Bu | Anisole | Open | - | - | - | - | - |

[a] **1** (2 mol%), KO$^t$Bu (8 mol%), Solvent (2 mL), 150 °C, 24 h. [b] 16 mol% base added. [c] 0.1 mL of deionized water added. [d] $T_g$ observed at –20.2 °C by DSC. $M_n$ (MALDI) value is estimated as the maximum signal observed on MALDI FT-ICR. Al:Ar = Aliphatic:Aromatic NMR integral relationship (see example in Figure S1). $T_d$ (°C) was recorded at 5% mass loss.

## 6. Spectral data of the isolated polyureas

Entry 1

Off-white solid (127 mg, 82% yield).

**$^1$H NMR** (500 MHz, *d*-TFA): $\delta_H$ 7.70-7.13 (br, R-CHO /Ar-H, 2.39H), 3.94-3.27 (br, R-$CH_2$-NRH, 4.19H), 1.98-1.31 (br, R-$CH_2$-R, 12.42H).

**$^{13}$C{$^1$H} NMR** (126 MHz, *d*-TFA): $\delta_C$ 132.3, 127.8, 127.3, 126.3, 125.6, 53.7, 30.3, 27.8.

**IR** (ATR-FTIR, $cm^{-1}$): ν 3309 (m, N-H), 2927 (s, C-H), 2850 (m, C-H), 1616 (s, C=O), 1558 (s, N-H/C-N), 1508 (s, N-H/C-N), 1301, 1236, 825, 621.

Entry 2

Brown solid (118 mg, 77% yield).

**$^1$H NMR** (500 MHz, *d*-TFA): $\delta_H$ 8.12-7.56 (br, R-CHO /Ar-H, 3.87H), 4.81 (br, R-NHR, 0.37H), 3.93 (br, R-$CH_2$-NRH, 1.40H), 3.28 (br, R-CH2-NRH, 3.34H), 1.60-1.32 (br, R-$CH_2$-R, 12H).

**$^{13}$C{$^1$H} NMR** (126 MHz, *d*-TFA): $\delta_C$ 159.0, 135.2, 131.2 128.8, 127.8, 126.3, 124.7, 124.3, 116.2, 65.8, 57.3, 43.6, 30.3, 27.8.

**IR** (ATR-FTIR, $cm^{-1}$): ν 3329 (m, N-H), 2929 (s, C-H), 2852 (m, C-H), 1610 (s, C=O), 1573 (s, N-H/C-N), 1508, (s, N-H/C-N), 1301, 1240, 829, 758, 623.

Entry 3

Dark brown solid (50 mg, 32% yield).

**$^1$H NMR** (500 MHz, *d*-TFA): $\delta_H$ 8.14-6.94 (br, R-CHO /Ar-H, 2.40H), 4.82 (br, R-NHR, 0.92H), 3.93 (br, R-CH2-NRH, 0.59H), 3.57-3.29 (br, R-CH2-NRH, 3.21H), 1.61-1.34 (br, R-$CH_2$-R, 12H).

**$^{13}$C{$^1$H} NMR** (126 MHz, *d*-TFA): $\delta_C$ 159.0, 135.3, 131.7, 131.1, 116.1, 65.8, 57.3, 43.3, 30.2, 27.7.

**IR** (ATR-FTIR, $cm^{-1}$): ν 3307 (w, N-H), 2926 (m, C-H), 2848 (m, C-H), 1616 (s, C=O) 1560 (s, N-H/C-N), 1508 (s, N-H/C-N), 1458, 1375, 1303, 1236, 829.

Entry 4

Off-white solid (122mg, 79% yield).

**$^1$H NMR** (500 MHz, *d*-TFA): $\delta_H$ 8.19 (br, R-CHO, 0.33H), 7.42 (br, Ar-H, 1.66H), 4.88 (br, R-NHR, 0.35H), 3.62-3.34 (br, R-CH2-NRH, 3.49H), 1.66-1.38 (br, R-$CH_2$-R, 12H).

**$^{13}$C{$^1$H} NMR** (126 MHz, *d*-TFA): $\delta_C$ 135.2, 131.7, 128.5, 43.6, 30.2, 27.7.

**IR** (ATR-FTIR, $cm^{-1}$): ν 3319 (m, N-H), 2929 (m, C-H), 2855 (m, C-H), 1618 (s, C=O), 1570 (s, N-H/C-N), 1510, (s, N-H/C-N), 1301, 1234, 1092, 827, 624.

Entry 5

Dark brown solid (68 mg, 44% yield).

**$^1$H NMR** (500 MHz, *d*-TFA): $\delta_H$ 7.93-7.73 (br, R-CHO /Ar-H, 0.82H), 3.75 (br, R-CH2-NRH, 0.18H), 3.50 (br, R-CH2-NRH, 2.08H), 3.17 (br, R-CH2-NRH, 1.33H), 2.42 (br, R-$CH_2$-R, 0.12H), 2.24-1.55 (br, R-$CH_2$-R, 12H).

**$^{13}$C{$^1$H} NMR** (126 MHz, *d*-TFA): $\delta_C$ 127.8, 126.4, 43.8, 30.4, 30.2, 27.9.

**IR** (ATR-FTIR, $cm^{-1}$): ν 3331 (m, N-H), 2927 (m, C-H), 2852 (m, C-H), 1612 (s, C=O), 1573 (s, N-H/C-N), 1512, (s, N-H/C-N), 1303, 1236, 1018, 948, 831.

Entry 6

Dark brown solid (45 mg, 30% yield).

**$^1$H NMR** (500 MHz, *d*-TFA): $\delta_H$ 8.17 (br, R-CHO, 0.50H), 7.51-7.37 (br, Ar-H, 1.81H), 4.86 (br, R-NHR, 0.55H), 3.98-3.34 (br, R-CH2-NRH, 3.85H), 1.66-1.38 (br, R-$CH_2$-R, 12.00H).

**$^{13}$C{$^1$H} NMR** (126 MHz, *d*-TFA): $\delta_C$ 135.3, 131.8, 128.6, 65.9, 43.9, 43.7, 30.3, 27.8.

**IR** (ATR-FTIR, $cm^{-1}$): ν 3307 (w, N-H), 2927 (m, C-H), 2852 (m, C-H), 1618 (s, C=O), 1560 (s, N-H/C-N), 1508 (s, N-H/C-N), 1382, 1301, 1226, 825.

Entry 7

Off-white solid (144 mg, 85% yield).

**$^1$H NMR** (500 MHz, *d*-TFA): $\delta_H$ 7.36 (br, R-CHO/ R-NHR, 0.01H), 4.11-4.04 (br, R-NHR / R-CH2-NRH, 0.07H), 3.65-3.39 (br, R-NHR / R-CH2-NRH, 3.94H), 1.71-1.43 (br, R-CH2-NRH, 12.00H).

**$^{13}C\{^1H\}$ NMR** (126 MHz, *d*-TFA): $\delta_C$ 43.8, 30.4, 30.2, 28.0.

IR (ATR-FTIR, cm$^{-1}$): ν 3327 (m, N-H), 2927 (m, C-H), 2850 (m, C-H), 1614 (m, C=O), 1573 (s, N-H/C-N), 1477, 1392, 1321, 1099.

Entry 8

Off-white solid (43 mg, 25% yield).

**$^1H$ NMR** (500 MHz, *d*-TFA): $\delta_H$ 6.66 (br, R-CHO/ R-NHR, 0.29H), 3.29 (br, R-CH2-NRH, 3.62H), 1.59-1.30 (br, R-CH2-NRH, 12H).

**$^{13}C\{^1H\}$ NMR** (126 MHz, *d*-TFA): $\delta_C$ 43.4, 30.0, 29.9, 27.5, 27.1.

**IR** (ATR-FTIR, cm$^{-1}$): ν 3331 (w, N-H), 2927 (m, C-H), 2850 (m, C-H), 1618 (m, C=O), 1571 (s, N-H/C-N), 1489, 1384, 1313, 817.

Entry 9

Off-white solid (134 mg, 78% yield).

**$^1H$ NMR** (500 MHz, *d*-TFA): $\delta_H$ 8.56 (br, R-CHO/ R-NHR, 0.07H), 7.62-6.63 (br, R-CHO/ R-NHR, 0.14H), 3.92-3.26 (br, R-CH2-NRH, 3.92H), 1.95-1.30 (br, R-$CH_2$-R, 12H).

**$^{13}C\{^1H\}$ NMR** (126 MHz, *d*-TFA): $\delta_C$ 43.8, 30.4, 30.3, 27.9.

**IR** (ATR-FTIR, cm$^{-1}$): ν 3321 (m, N-H), 2926 (m, C-H), 2850 (m, C-H), 1614 (s, C=O), 1568 (s, N-H/C-N), 1477, 1240, 1010.

Entry 10

Off-white solid (114mg, 67% yield).

**$^1H$ NMR** (500 MHz, *d*-TFA): $\delta_H$ 8.80 (br, R-CHO/ R-NHR, 0.00H), 7.12 (br, R-CHO/ R-NHR, 0.00H), 3.78 (br, R-CH2-NRH, 0.16H), 3.52-3.21 (br, R-CH2-NRH, 5.45H), 2.46 (br, R-CH2-NRH /R-$CH_2$-R, 0.09H), 1.97-1.56 (br, R-$CH_2$-R, 12H).

**$^{13}C\{^1H\}$ NMR** (126 MHz, *d*-TFA): $\delta_C$ 43.7, 30.3, 30.2, 30.1, 29.4, 28.7, 27.8, 27.4.

**IR** (ATR-FTIR, cm$^{-1}$): ν 3329 (m, N-H), 2926 (m, C-H), 2850 (m, C-H), 1614 (s, C=O), 1570 (s, N-H/C-N), 1475, 1301, 1240, 1016, 952.

Entry 11

Dark brown solid (2.2 mg, 2% yield).

**$^{1}$H NMR** (500 MHz, *d*-TFA): $\delta_H$ 7.31 (br, R-CHO/ R-NHR, 0.11H), 4.06-4.00 (br, R-CH2-NRH, 0.14H), 3.66-3.36 (br, R-CH2-NRH /R-$CH_2$-R, 3.57H), 1.68-1.30 (br, R-$CH_2$-R, 12H).

**$^{13}$C{$^{1}$H} NMR** (126 MHz, *d*-TFA): $\delta_C$ 43.8, 31.4, 30.4, 30.1, 27.9.

**IR** (ATR-FTIR, $cm^{-1}$): ν 3280 (m, N-H), 2929 (m, C-H), 2850 (m, C-H), 1612 (s, C=O), 1571 (s, N-H/C-N), 1462, 1379, 1236, 1082, 1020, 979, 779.

Entry 12

Dark brown solid (49.3 mg, 29% yield).

**$^{1}$H NMR** (500 MHz, *d*-TFA): $\delta_H$ 7.22 (br, R-CHO/ R-NHR, 0.04H), 3.96-3.26 (br, R-CH2-NRH /R-$CH_2$-R, 4.02H), 1.58-1.30 (br, R-$CH_2$-R, 12H).

**$^{13}$C{$^{1}$H} NMR** (126 MHz, *d*-TFA): $\delta_C$ 43.3, 30.0, 29.7, 27.5.

**IR** (ATR-FTIR, $cm^{-1}$): ν 3329 (m, N-H), 2927 (m, C-H), 2851 (m, C-H), 1610 (s, C=O), 1573 (s, N-H/C-N), 1464, 1381, 1301, 1240, 1082.

Entry 13

Off-white solid (49.3 mg, 29% yield).

**$^{1}$H NMR** (500 MHz, *d*-TFA): $\delta_H$ 6.68 (br, R-C(=O)H/ R-NHR, 0.15H), 3.95-3.32 (br, R-CH2-NRH /R-$CH_2$-R, 3.83H), 1.63-1.34 (br, R-$CH_2$-R, 12H).

**$^{13}$C{$^{1}$H} NMR** (126 MHz, *d*-TFA): $\delta_C$ 43.6, 30.2, 30.0, 27.7.

**IR** ATR-FTIR, $cm^{-1}$): ν 3327 (m, N-H), 2927 (m, C-H), 2850 (m, C-H), 1612 (s, C=O), 1573 (s, N-H/C-N), 1477, 1382, 1300, 1240, 1087.

Entry 14

Brown solid (180 mg, 94 % yield)

**$^1$H NMR** (500 MHz, $D_2O$): $\delta_H$ 8.39 (terminal COH), 8.15 (C(=O)H/N-H), 8.00 (COH/NH), 7.47-6.70 (multiple signals, 2H, Ar-H), 3.66-3.63 (overlapping signals, 6H, $C\underline{H}_2O$) 3.20 – 3.09 (multiple signals, 2H, $C\underline{H}_2N$) and 1.79-1.71 (m, 2H, $C\underline{H}_2C$).

**$^{13}$C{$^1$H} NMR** (126 MHz, $D_2O$): $\delta_C$ 164.1 (N$\underline{C}$OH), 162.4 (N$\underline{C}$OH), 160.0 (NH$\underline{C}$ONH), 159.1 (NH$\underline{C}$ONH), 138.4 (Ar-q$\underline{C}$-NHCONH), 129.8 (Ar-q$\underline{C}$-NHCONH), 123.5 (Ar-q$\underline{C}$-NHCOH), 121.6 (Ar-$\underline{C}$-qC-NHCOH), 117.8 (Ar-C), 116.9 (Ar-C), 114.0 (Ar-C), 69.9 ($\underline{C}$-O), 69.5 ($\underline{C}$-O), 69.3 ($\underline{C}$-O), 69.0 ($\underline{C}$-O), 68.6 ($\underline{C}$-O), 68.4 ($\underline{C}$-O), 68.2 ($\underline{C}$-O), 37.0 ($\underline{C}$-$NH_2$), 36.9 ($\underline{CH_2}$-$NH_2$), 35.0 ($\underline{C}H_2$-N), 29.1 ($CH_2$), 28.1 ($CH_2$), 27.6 ($CH_2$).

**IR** (ATR-FTIR, $cm^{-1}$): ν 3302 (m, N-H), 2866 (m, C-H), 1618 (s, C=O), 1521 (s, C=O), 1512 (vs, C-O), 1257, 1088, 823.

**GPC** ($H_2O$, 30 °C): $M_n$ 44,000, PDI 1.4.

End group analysis using the $^1$H NMR spectrum obtained of the sample (soluble in $D_2O$) estimates MW as 19,000 g $mol^{-1}$.

**MALDI-TOF MS** measurement highest *m*/*z* observed was ~3140 Da.

<u>Entry 15</u>

Brown solid (42 mg)

No urea linkage observed by $^{13}$C{$^1$H} NMR or IR spectroscopies.

## 7. NMR spectra of the isolated polyureas

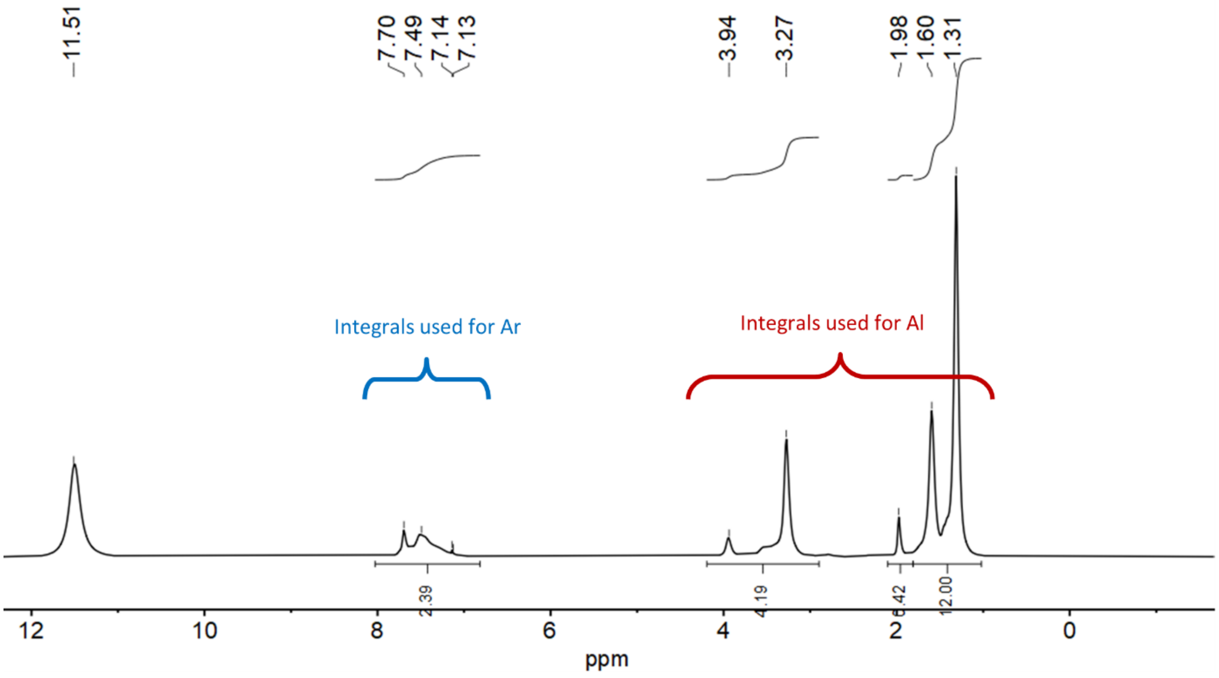


**Figure S1** $^{1}H$ NMR spectrum (d-TFA, 500 MHz, 298 K) of Entry 1. Blue/red indicated areas = signal integrals used for calculation of the aromatic/aliphatic relationship shown in Table S1.

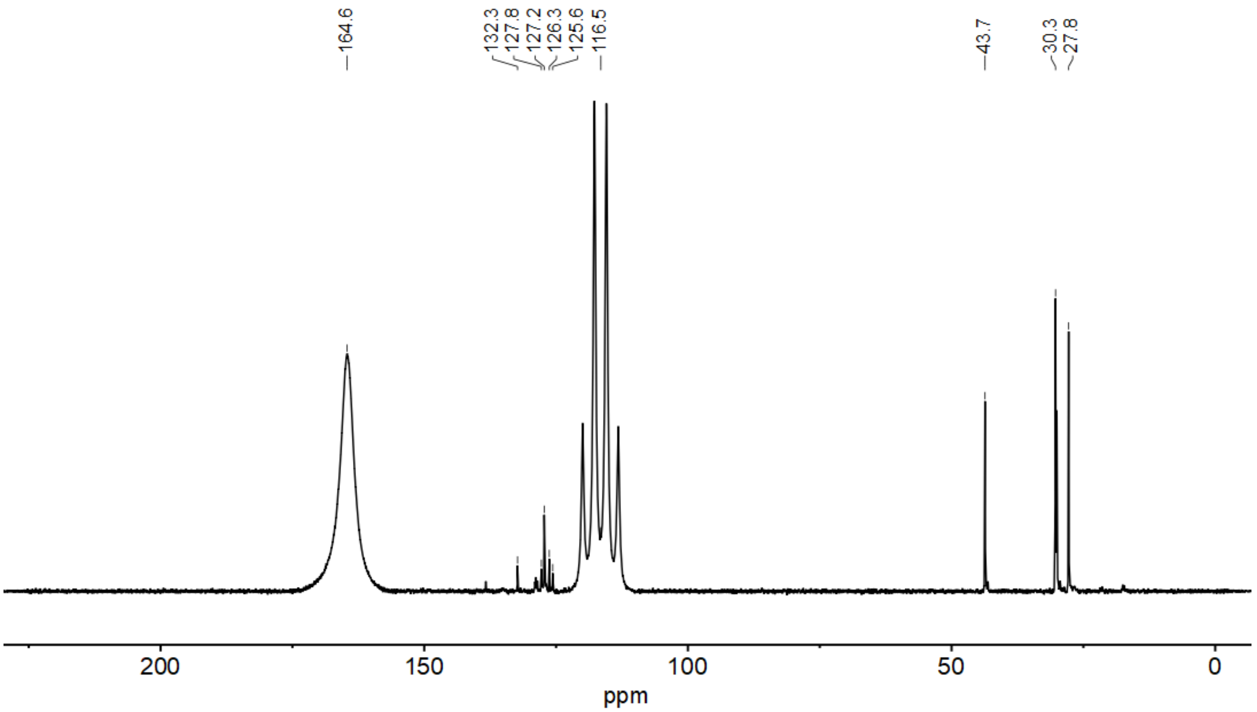


**Figure S2** $^{13}C\{^{1}H\}$ NMR spectrum (d-TFA, 126 MHz, 298 K) of Entry 1.

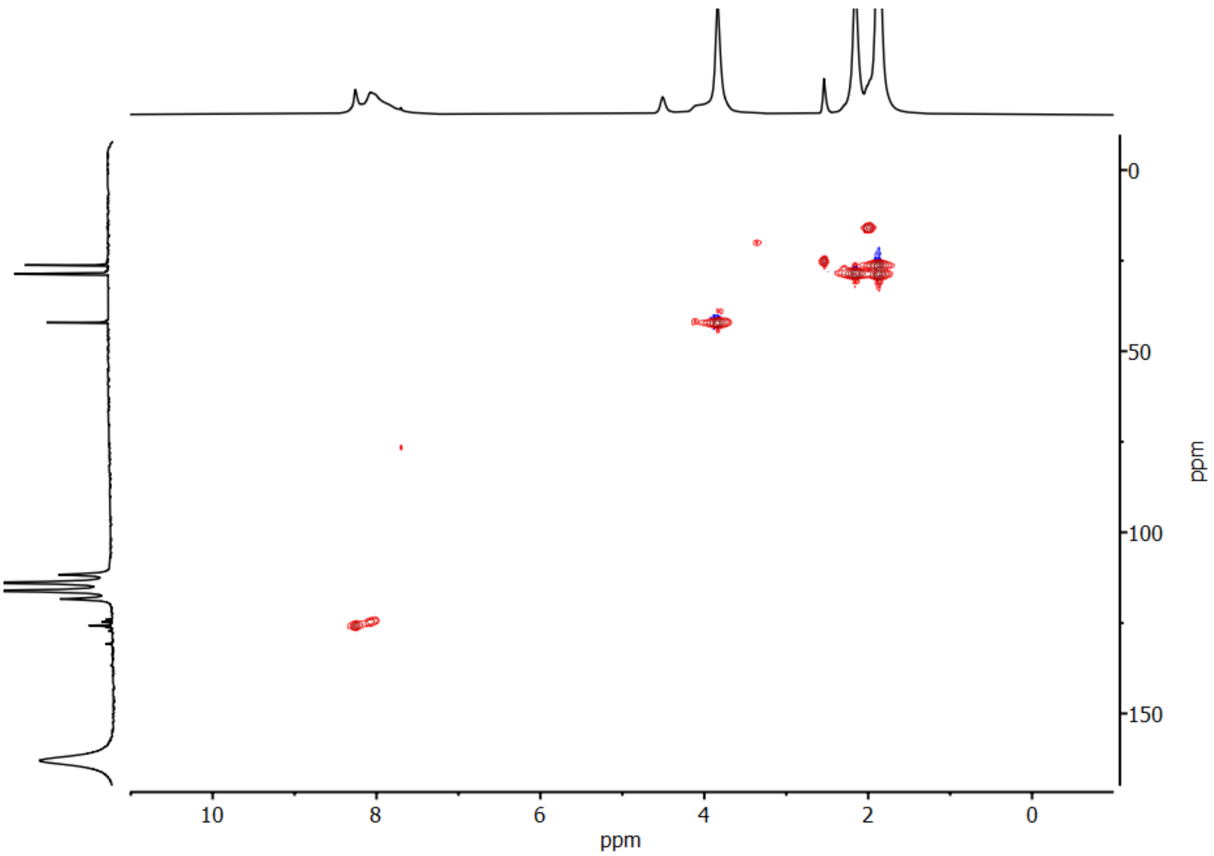


**Figure S3** $^{1}H$, $^{13}C$-HSQC NMR spectrum (d-TFA, 500-126 MHz, 298 K) of Entry 1.

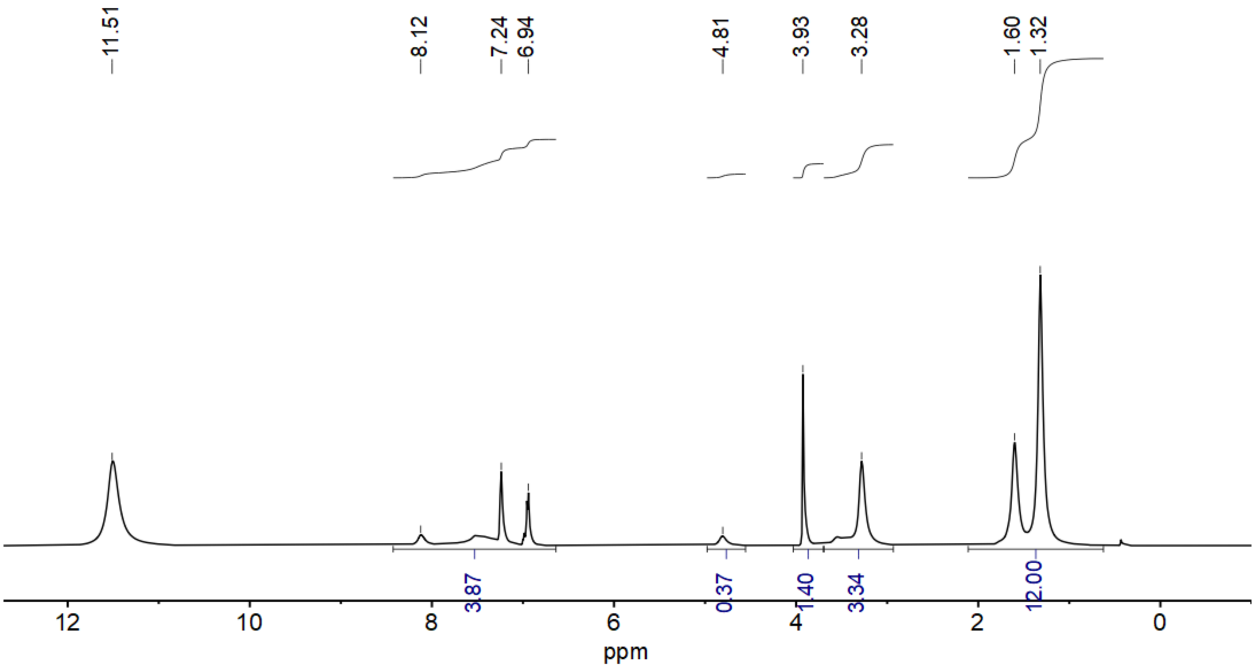


**Figure S4** $^{1}H$ NMR spectrum (d-TFA, 500 MHz, 298 K) of Entry 2.

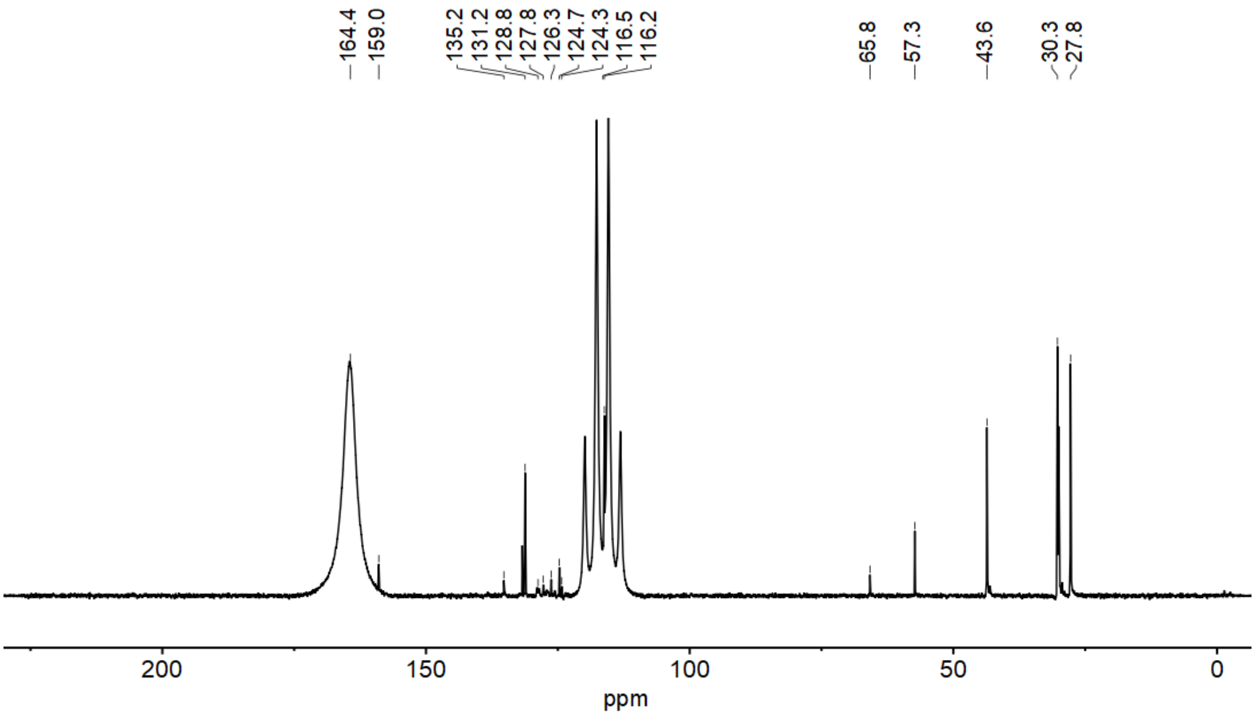


**Figure S5** $^{13}C\{^{1}H\}$ NMR spectrum (d-TFA, 126 MHz, 298 K) of Entry 2.

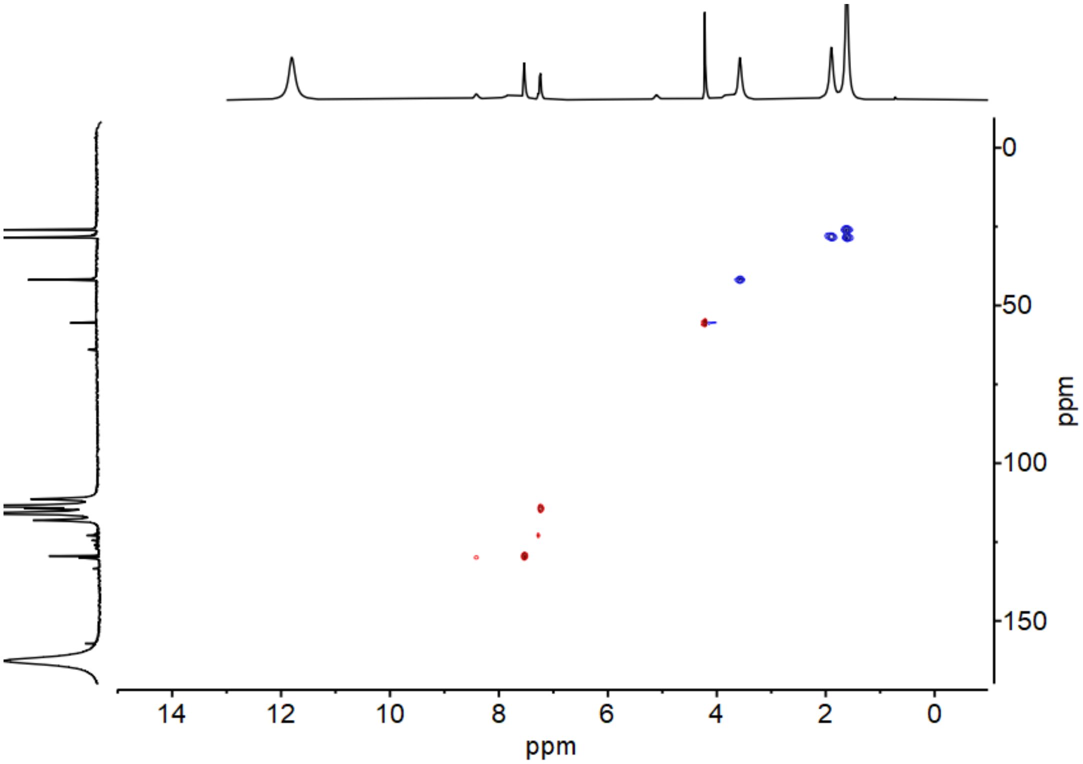


**Figure S6** $^{1}H,^{13}C$-HSQC NMR spectrum (d-TFA, 500-126 MHz, 298 K) spectrum for Entry 2.

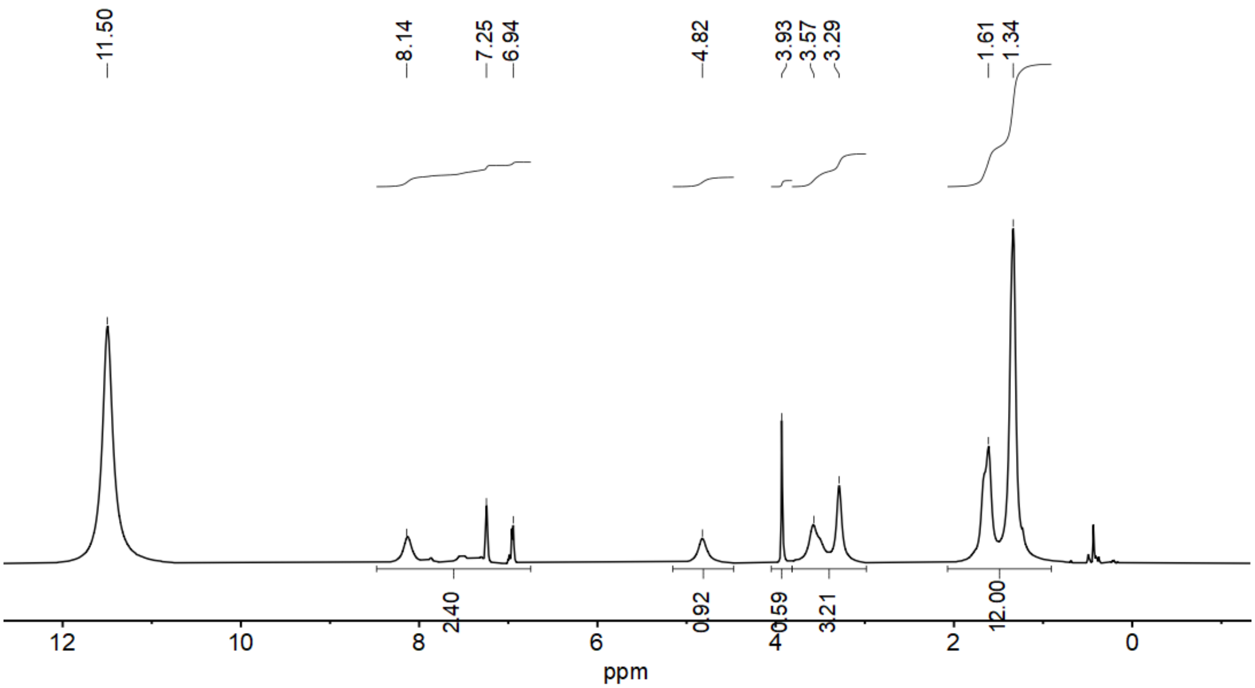


**Figure S7** $^{1}H$ NMR spectrum (d-TFA, 500 MHz, 298 K) of Entry 3.

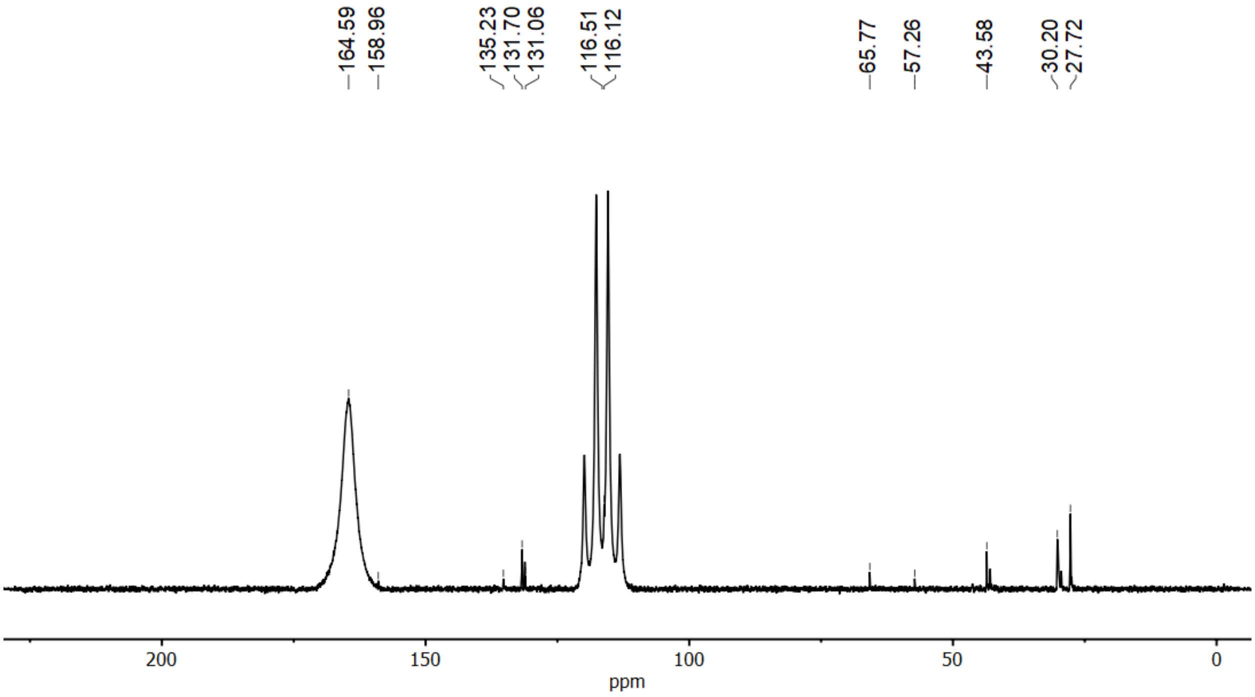


**Figure S8** $^{13}C\{^{1}H\}$ NMR spectrum (d-TFA, 126 MHz, 298 K) of Entry 3.

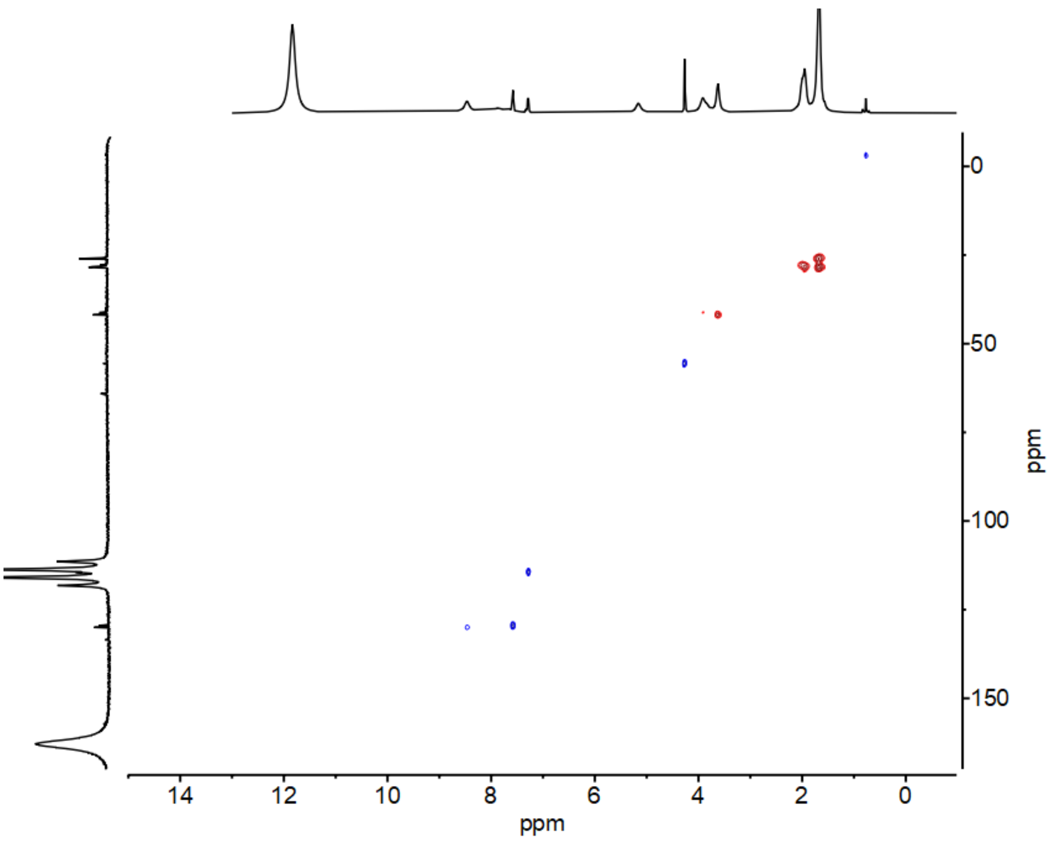


**Figure S9** $^{1}H$,$^{13}C$-HSQC NMR spectrum (d-TFA, 500-126 MHz, 298 K) spectrum of Entry 3.

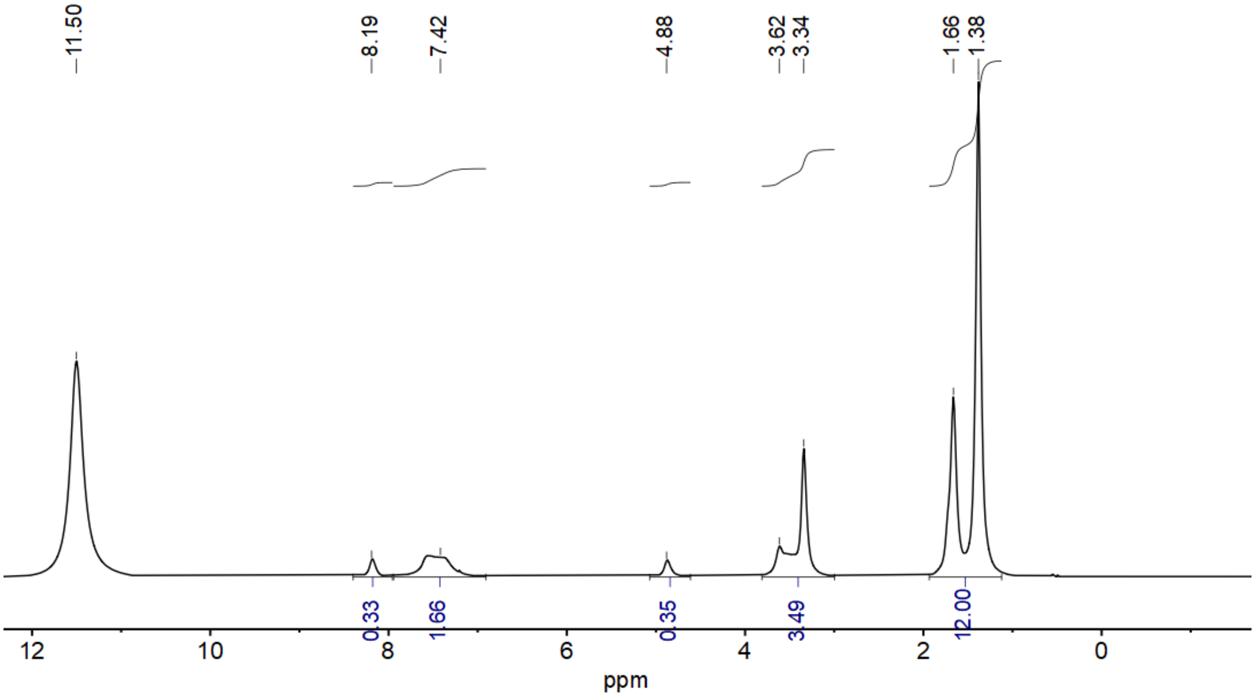


**Figure S10** $^{1}H$ NMR spectrum (d-TFA, 500 MHz, 298 K) of Entry 4.

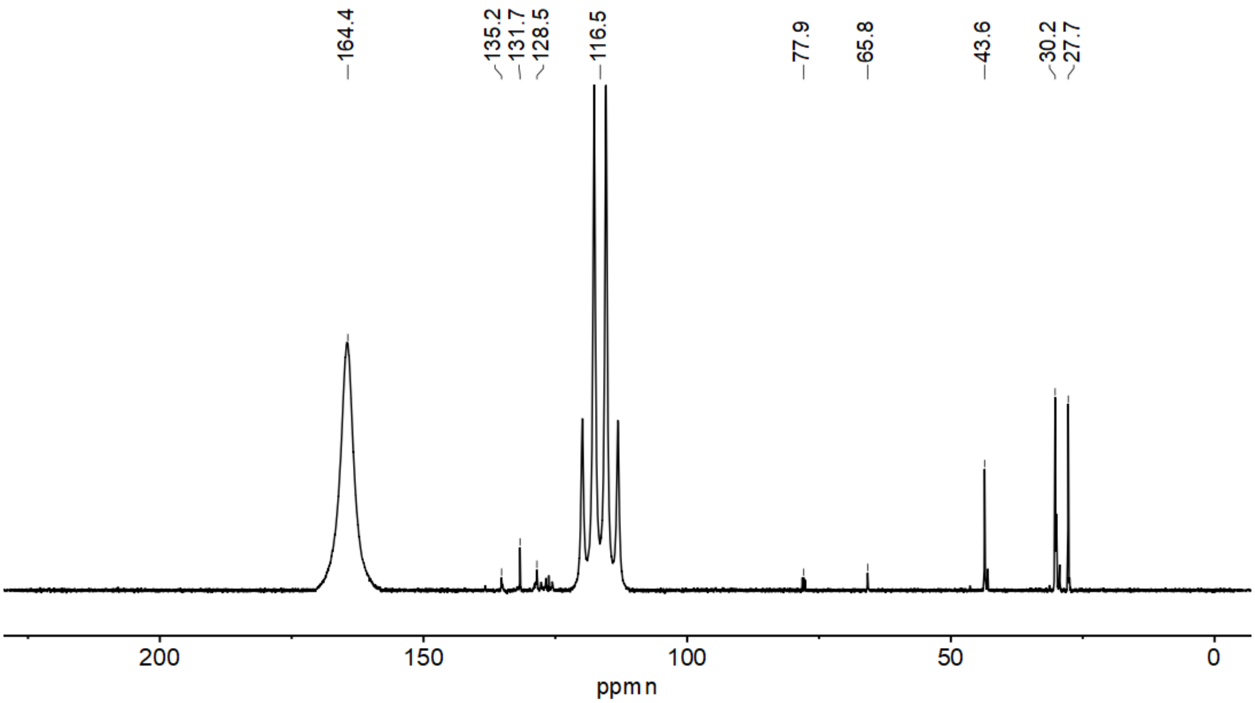


**Figure S11** $^{13}C\{^{1}H\}$ NMR spectrum (d-TFA, 126 MHz, 298 K) of Entry 4.

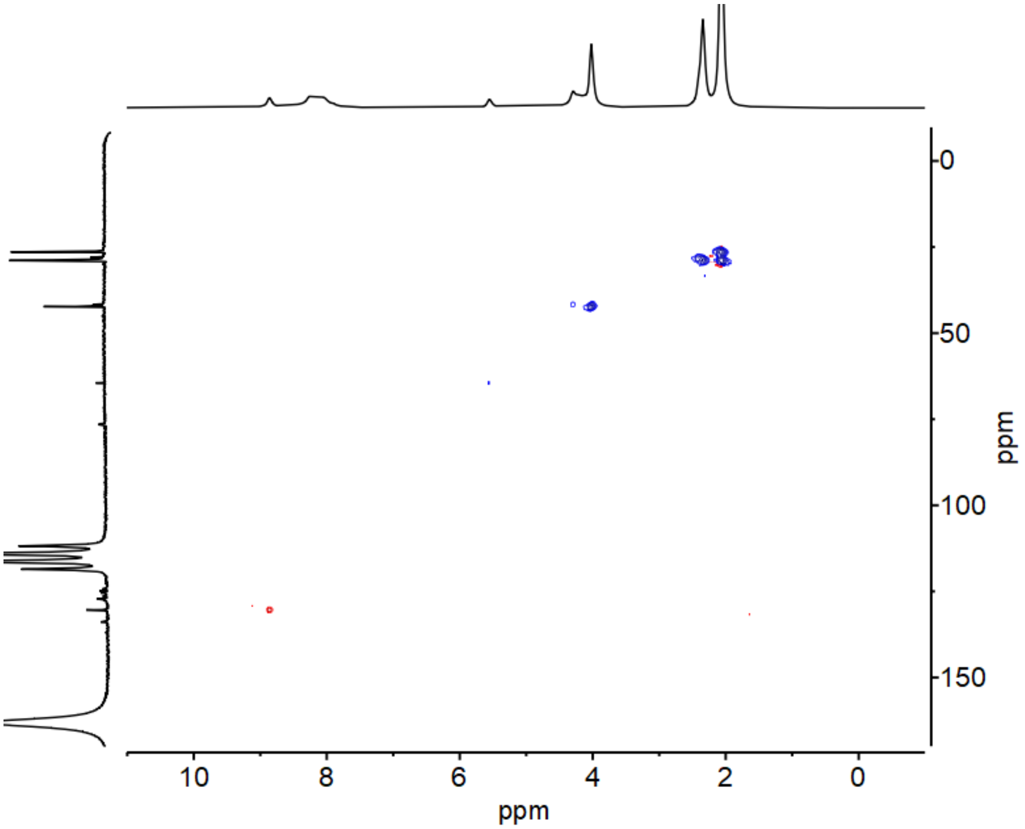


**Figure S12** $^{1}H$, $^{13}C$-HSQC NMR spectrum (d-TFA, 500-126 MHz, 298 K) spectrum of Entry 4.

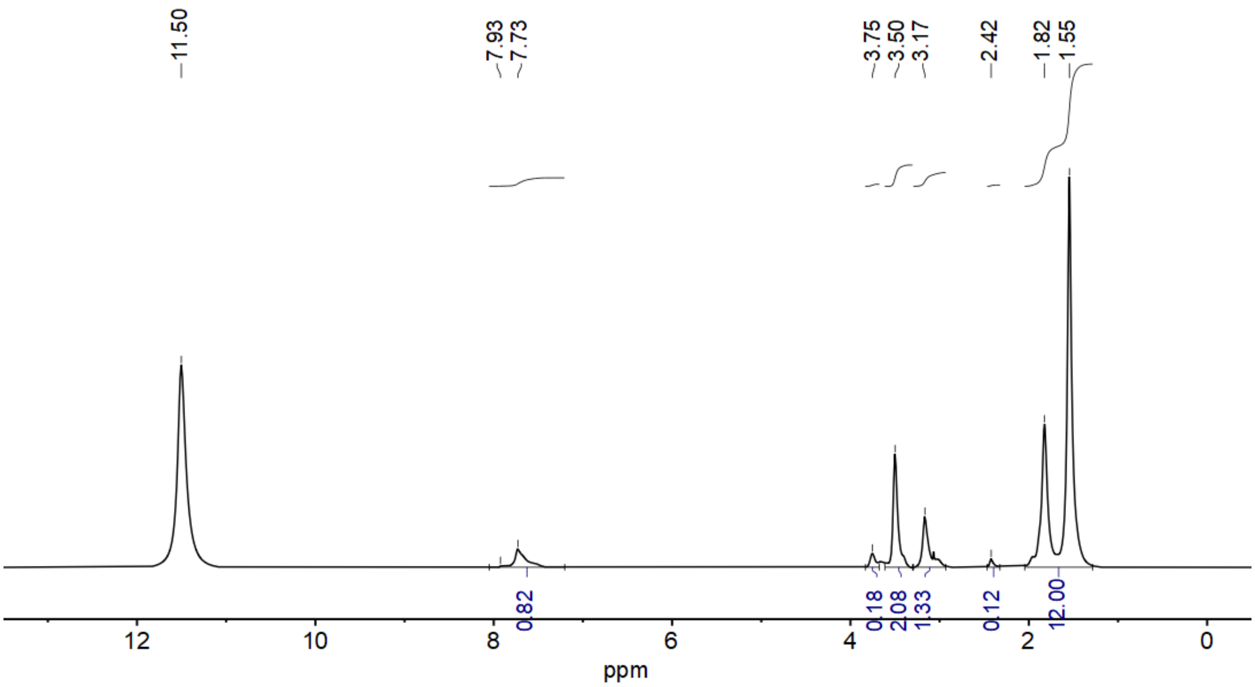


**Figure S13** $^{1}H$ NMR spectrum (d-TFA, 500 MHz, 298 K) of Entry 5.

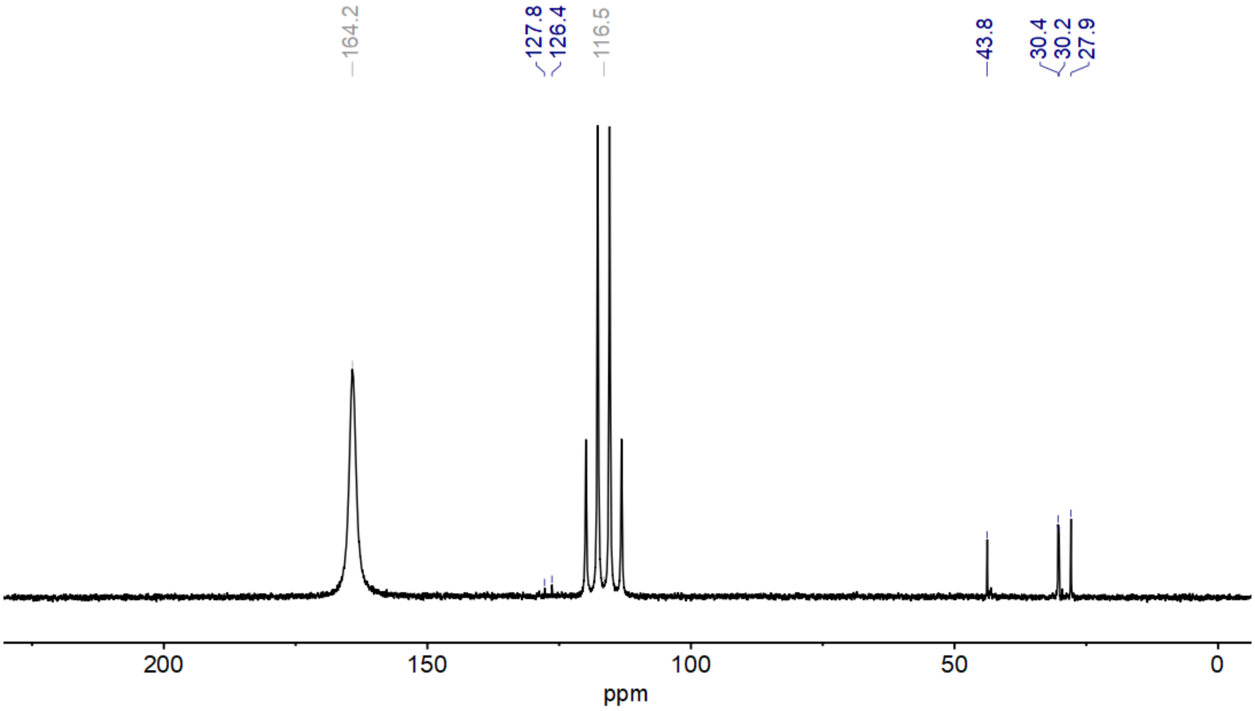


**Figure S14** $^{13}C\{^{1}H\}$ NMR spectrum (d-TFA, 126 MHz, 298 K) of Entry 5.

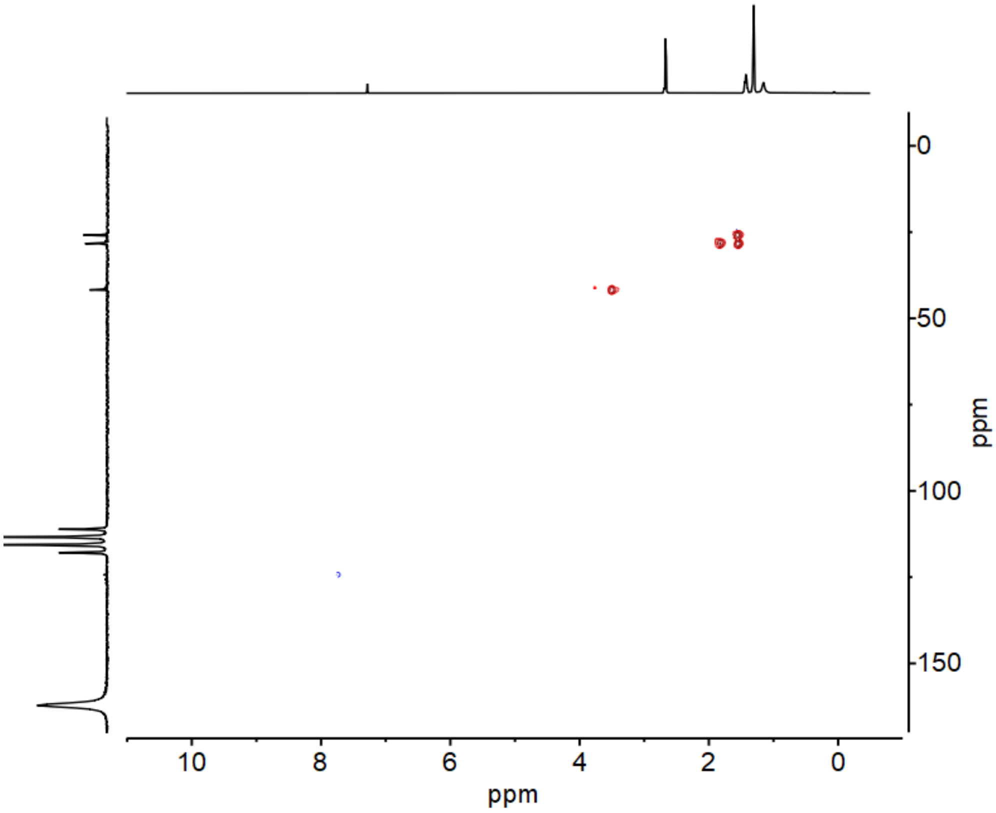


**Figure S15** $^{1}H$, $^{13}C$-HSQC NMR spectrum (d-TFA, 500-126 MHz, 298 K) of Entry 5.

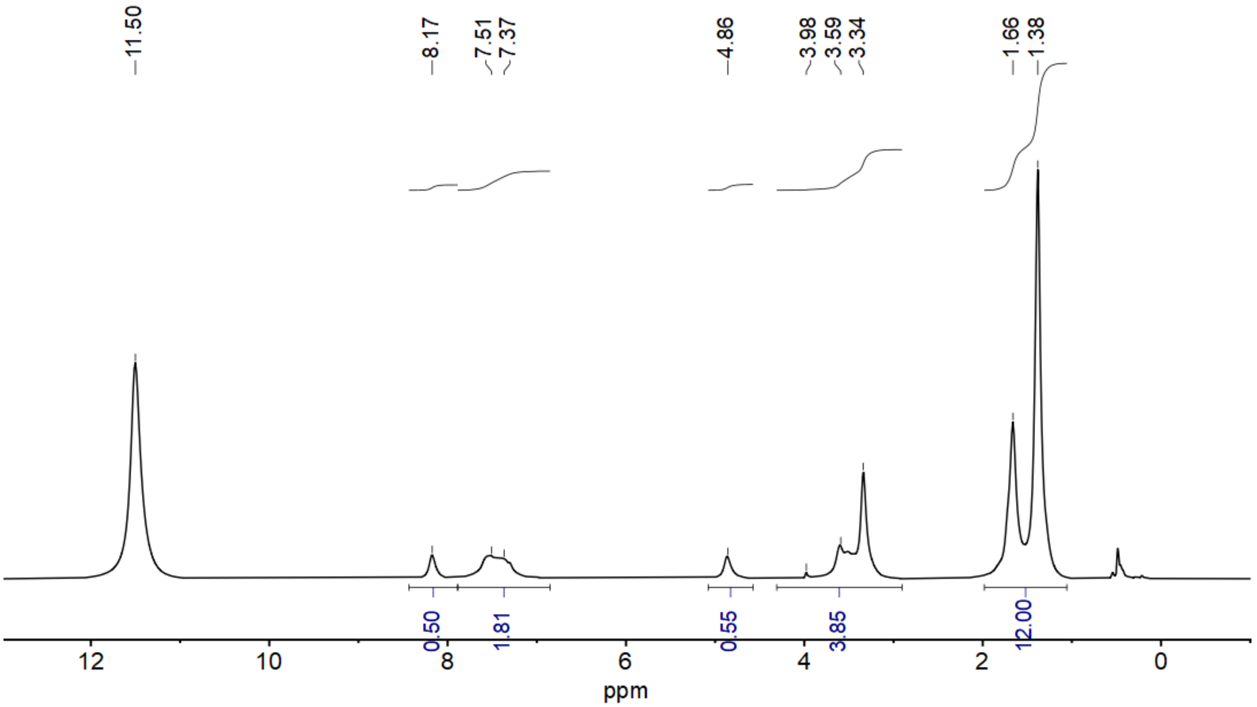


**Figure S16** $^{1}H$ NMR spectrum (d-TFA, 500 MHz, 298 K) of Entry 6.

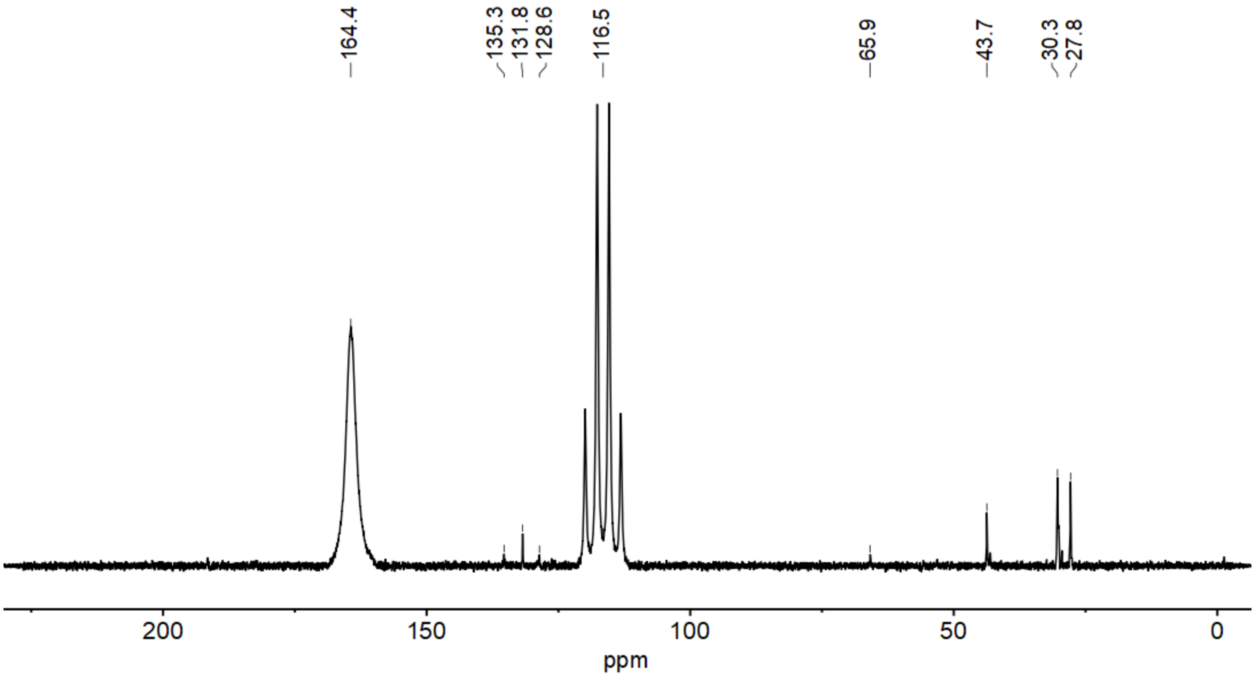


**Figure S17** $^{13}C\{^{1}H\}$ NMR spectrum (d-TFA, 126 MHz, 298 K) of Entry 6.

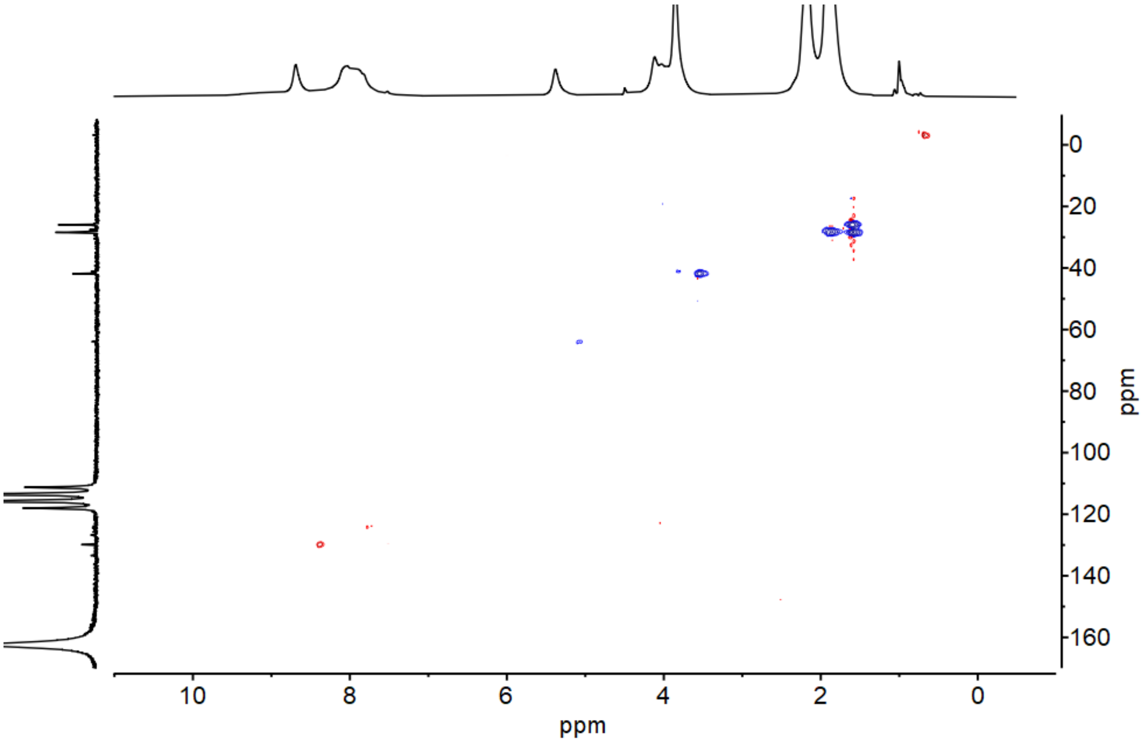


**Figure S18** $^{1}H$, $^{13}C$-HSQC NMR spectrum (d-TFA, 500-126 MHz, 298 K) of Entry 6.

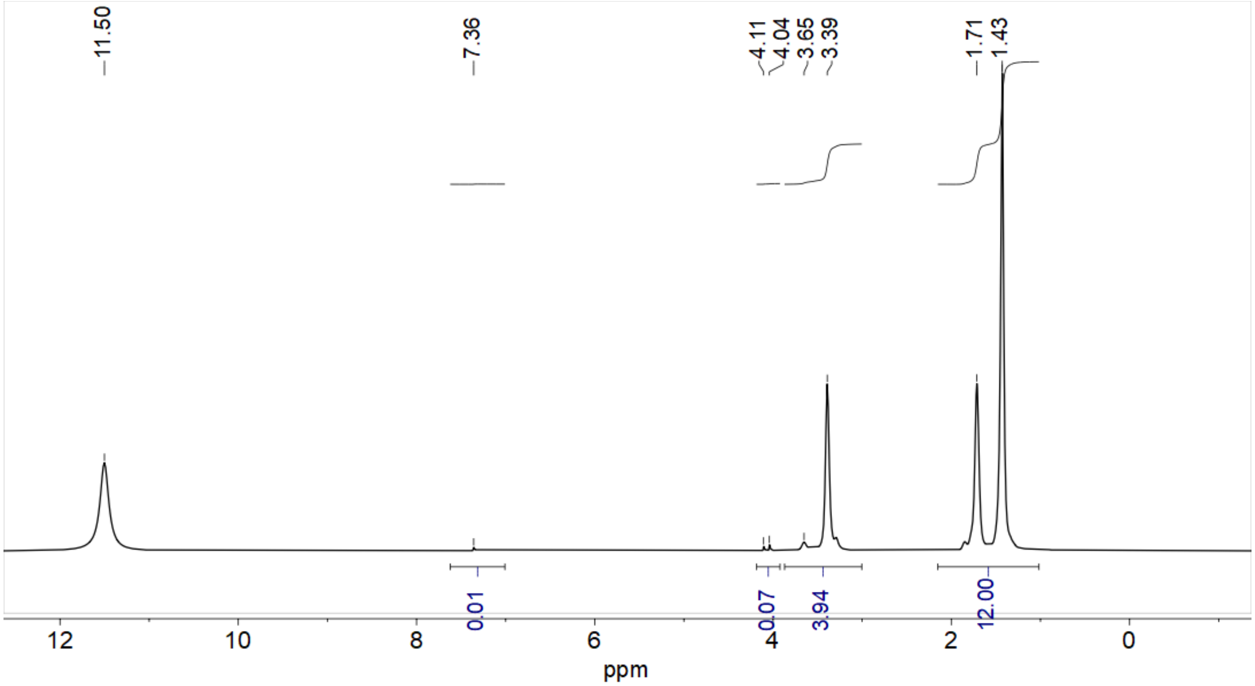


**Figure S19** $^{1}H$ NMR spectrum (d-TFA, 500 MHz, 298 K) of Entry 7.

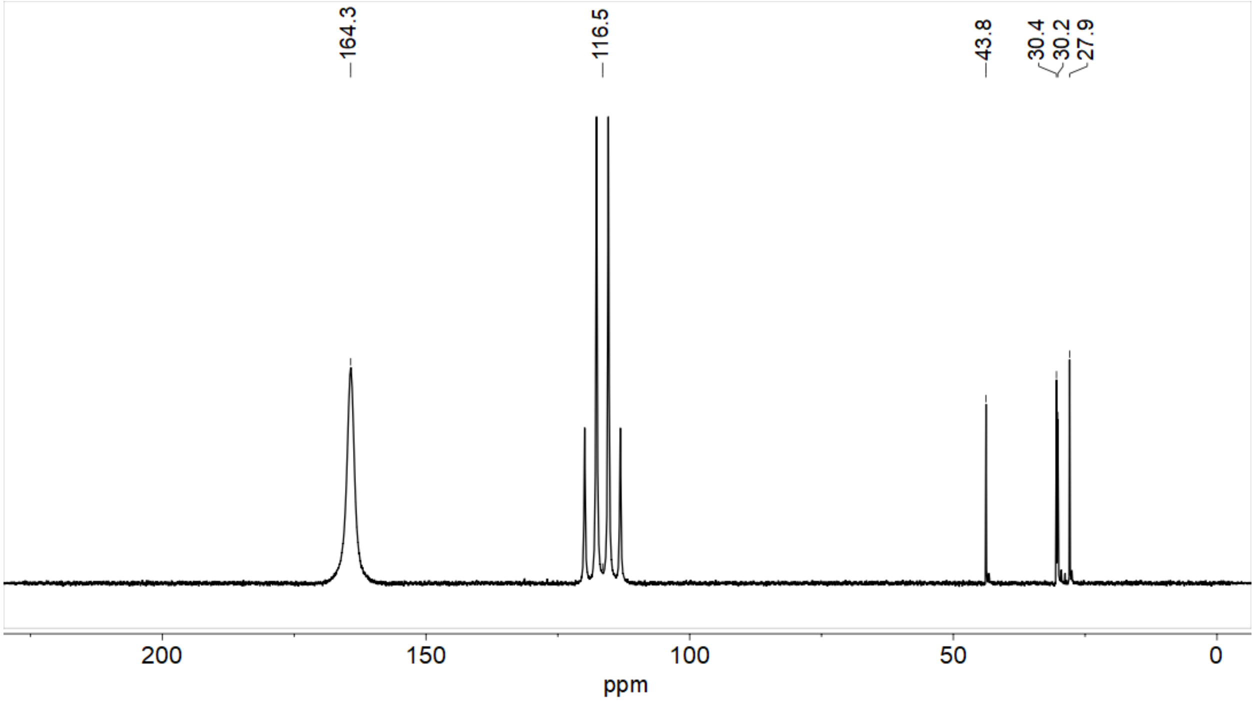


**Figure S20** $^{13}C\{^{1}H\}$ NMR spectrum (d-TFA, 126 MHz, 298 K) of Entry 7.

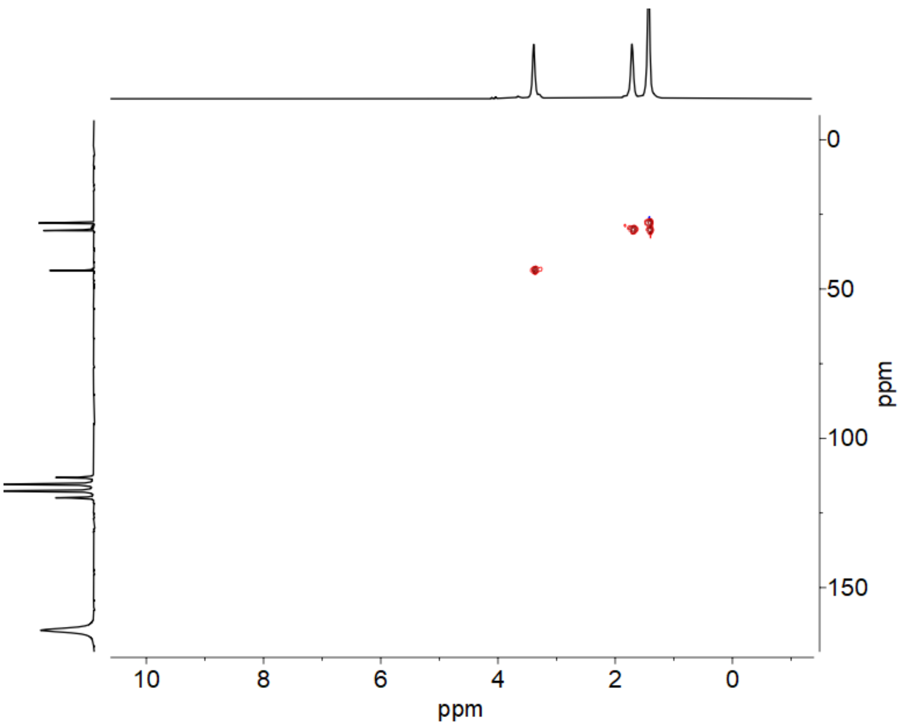


**Figure S21** $^{1}$H, $^{13}$C-HSQC NMR spectrum (d-TFA, 500-126 MHz, 298 K) of Entry 7.

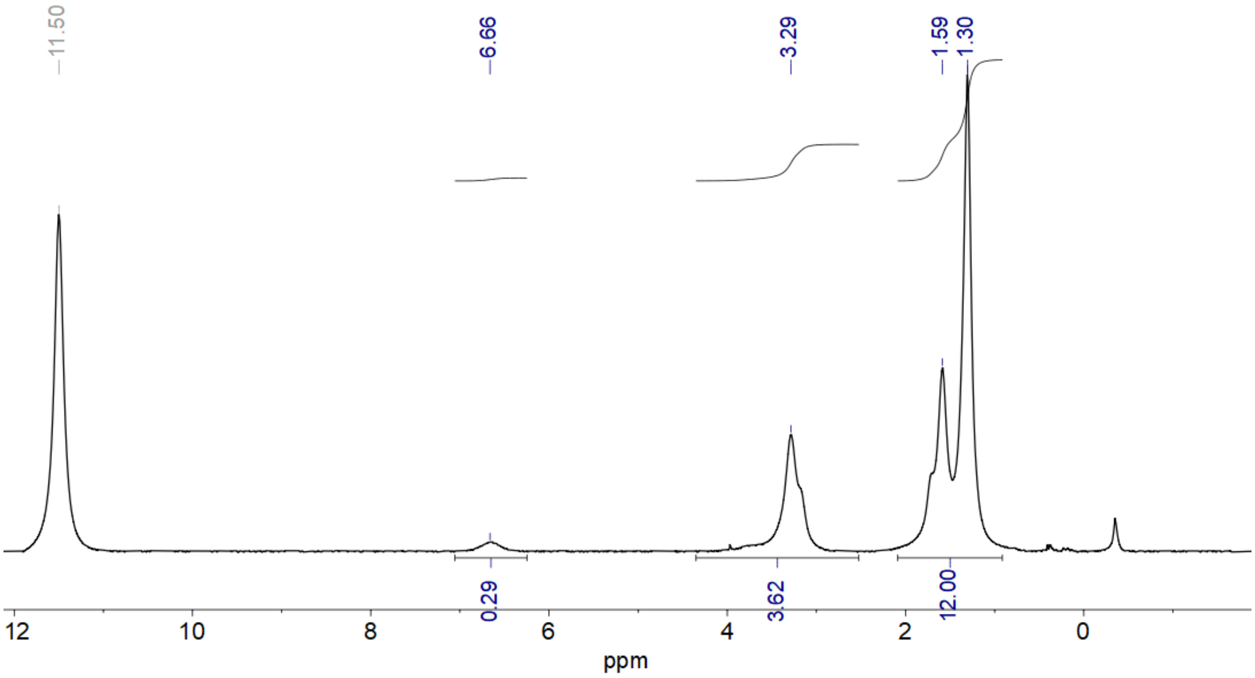


**Figure S22** $^{1}$H NMR spectrum (d-TFA, 500 MHz, 298 K) of Entry 8.

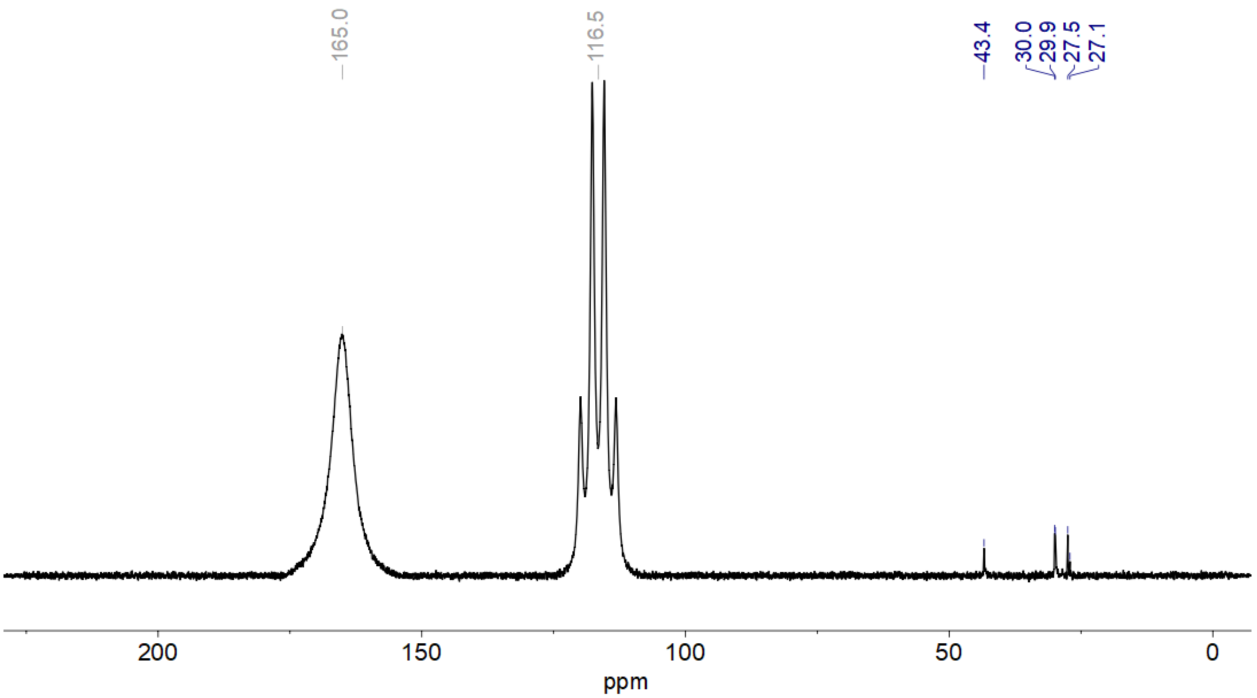


**Figure S23** $^{13}C\{^1H\}$ NMR spectrum (d-TFA, 126 MHz, 298 K) of Entry 8.

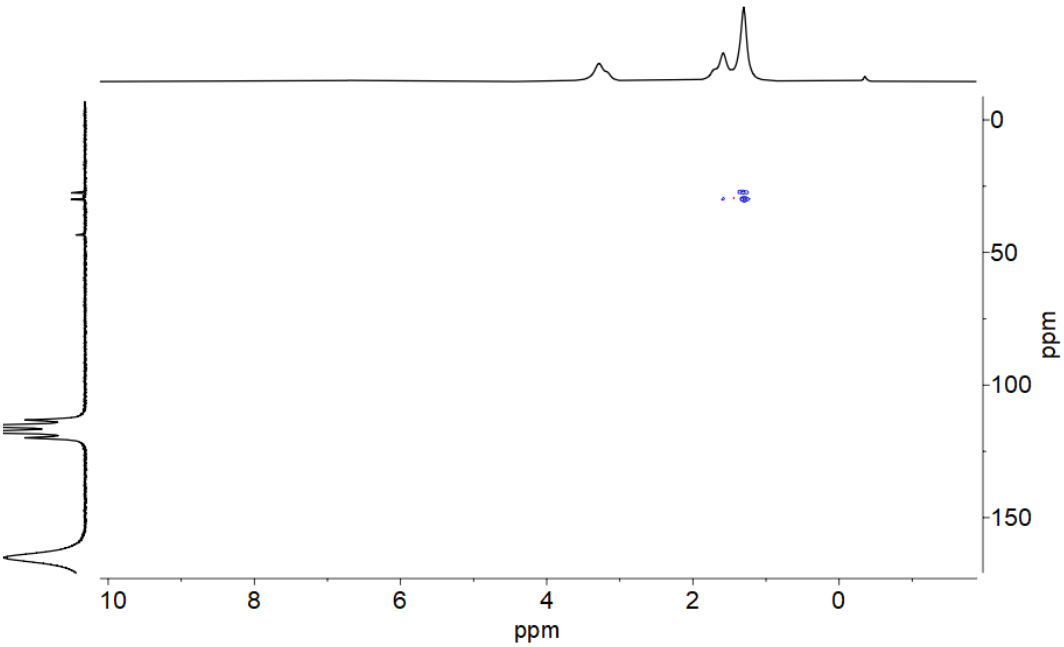


**Figure S24** $^1H$, $^{13}C$-HSQC NMR spectrum (d-TFA, 500-126 MHz, 298 K) of Entry 8.

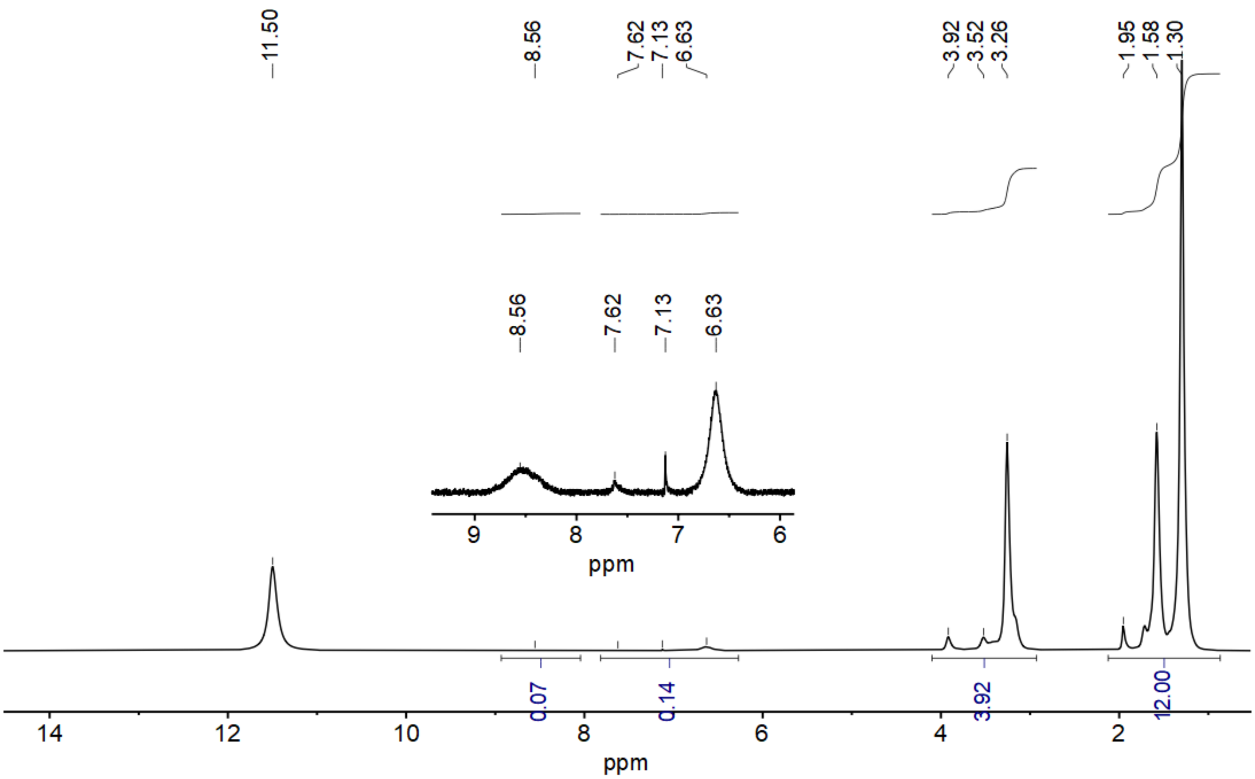


**Figure S25** $^{1}H$ NMR spectrum (d-TFA, 500 MHz, 298 K) of Entry 9.

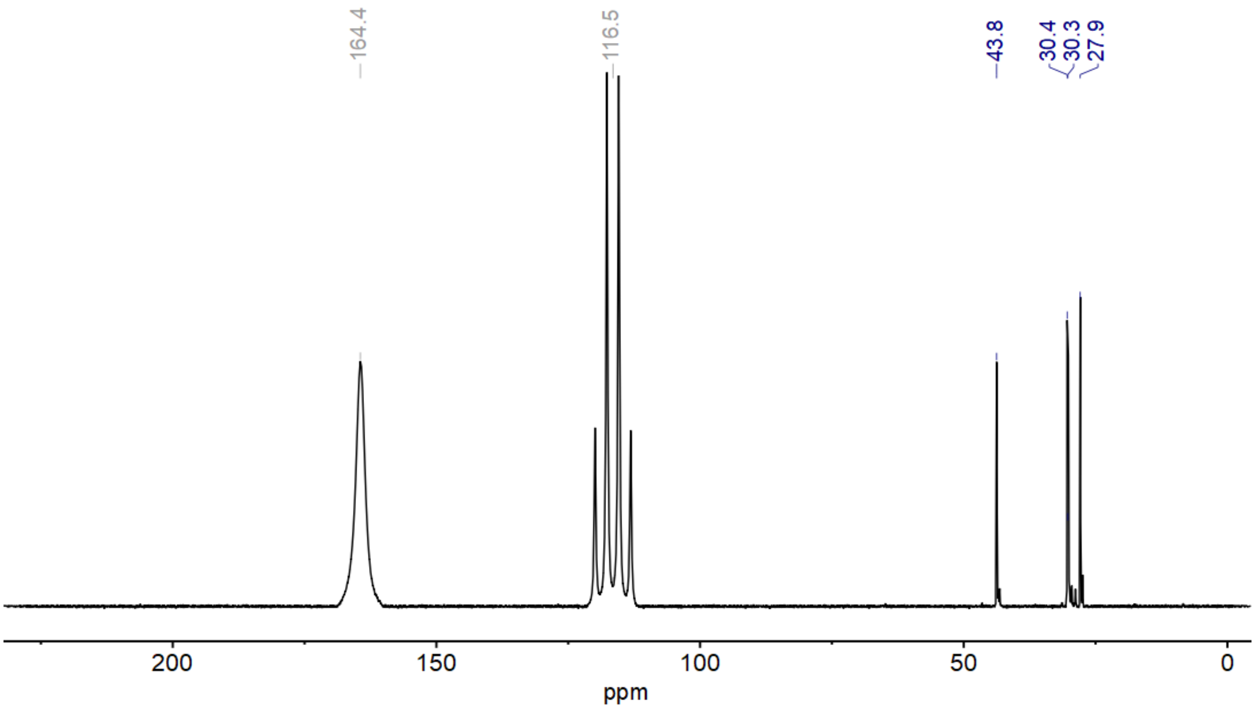


**Figure S26** $^{13}C\{^{1}H\}$ NMR spectrum (d-TFA, 126 MHz, 298 K) of Entry 9.

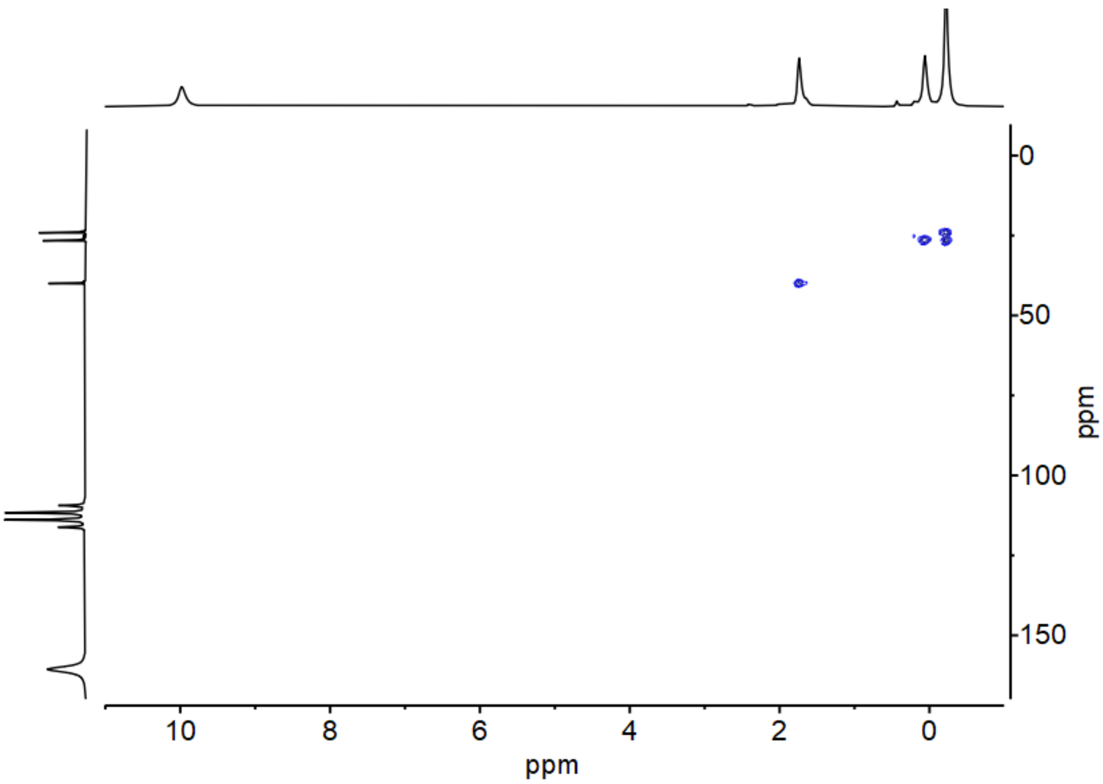


**Figure S27** $^{1}H$, $^{13}C$-HSQC NMR spectrum (d-TFA, 500-126 MHz, 298 K) of Entry 9.

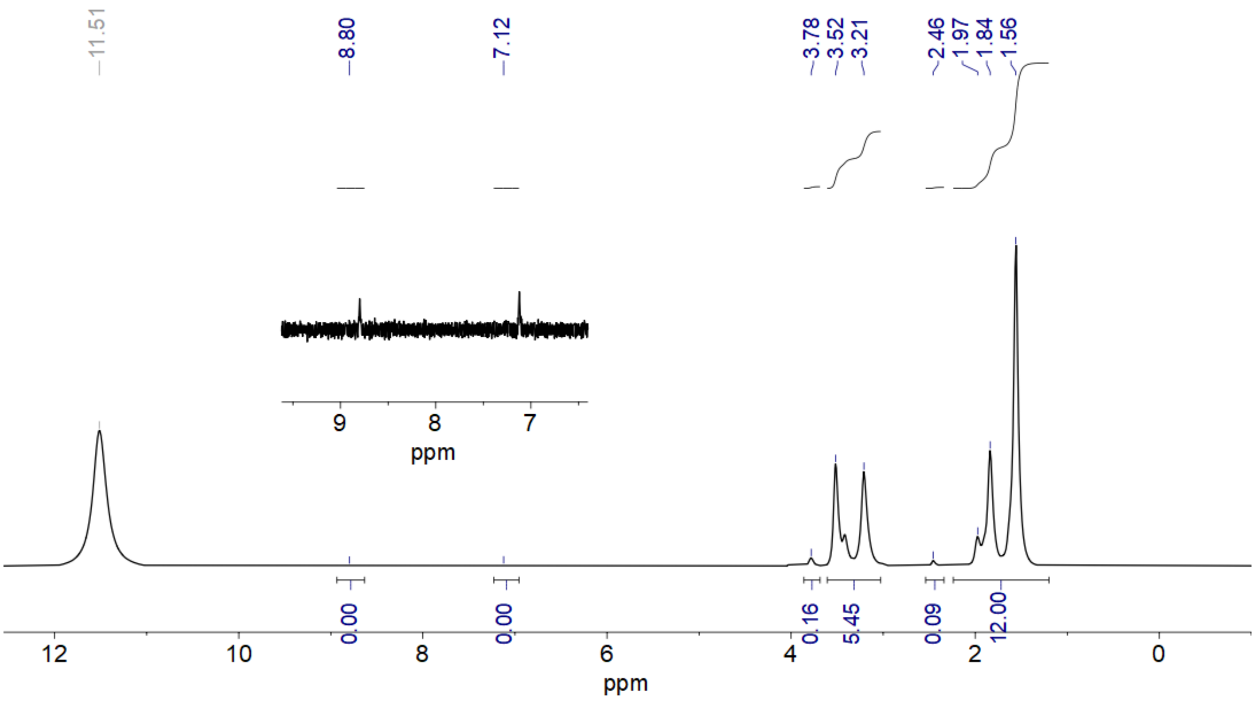


**Figure S28** $^{1}H$ NMR spectrum (d-TFA, 500 MHz, 298 K) of Entry 10.

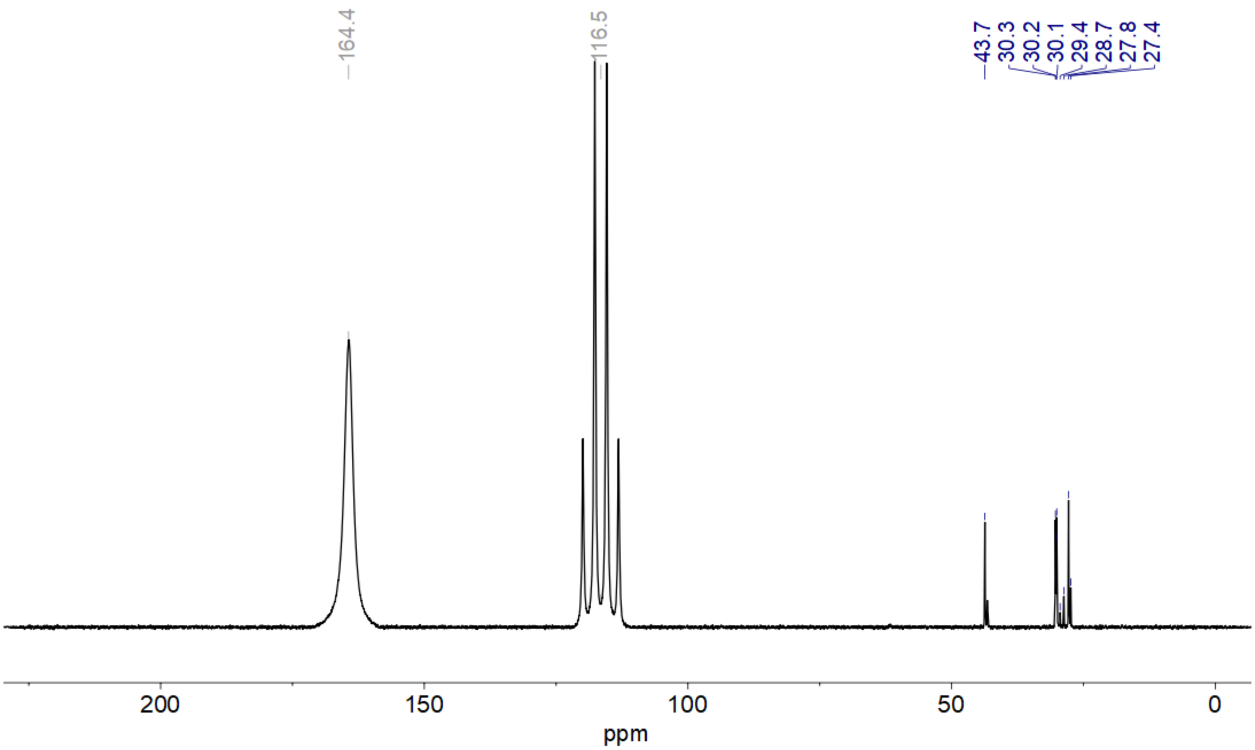


**Figure S29** $^{13}C\{^{1}H\}$ NMR spectrum (d-TFA, 126 MHz, 298 K) of Entry 10.

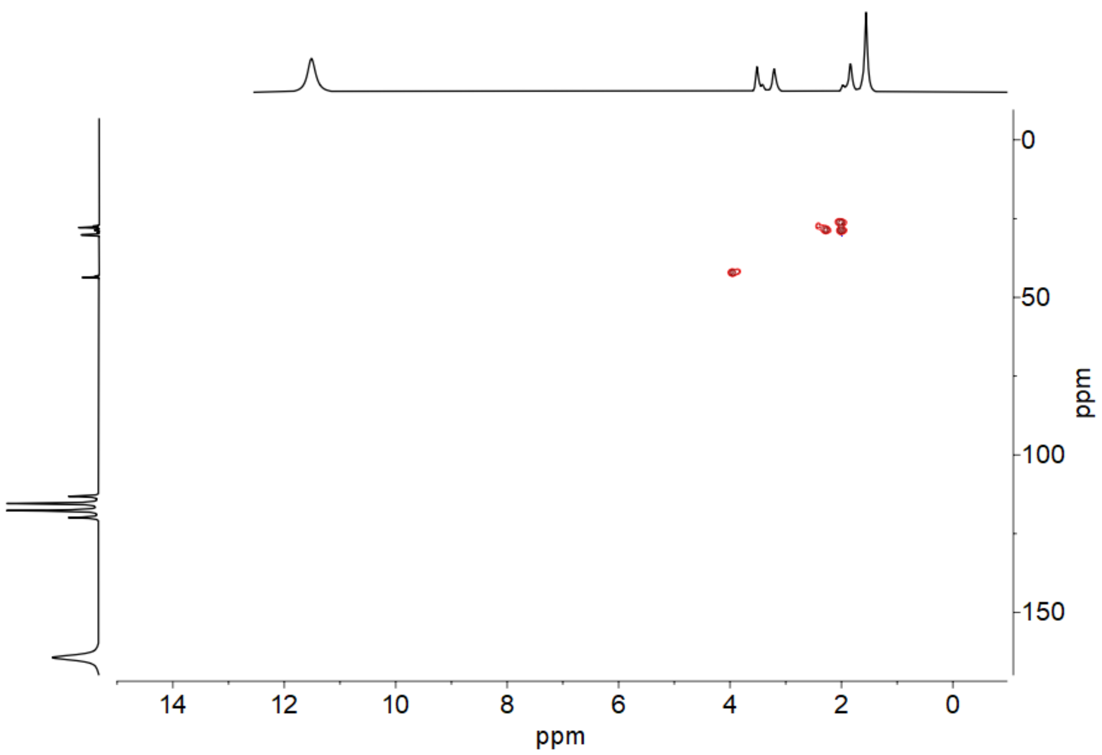


**Figure S30** $^{1}H$, $^{13}C$-HSQC NMR spectrum (d-TFA, 500-126 MHz, 298 K) of Entry 10.

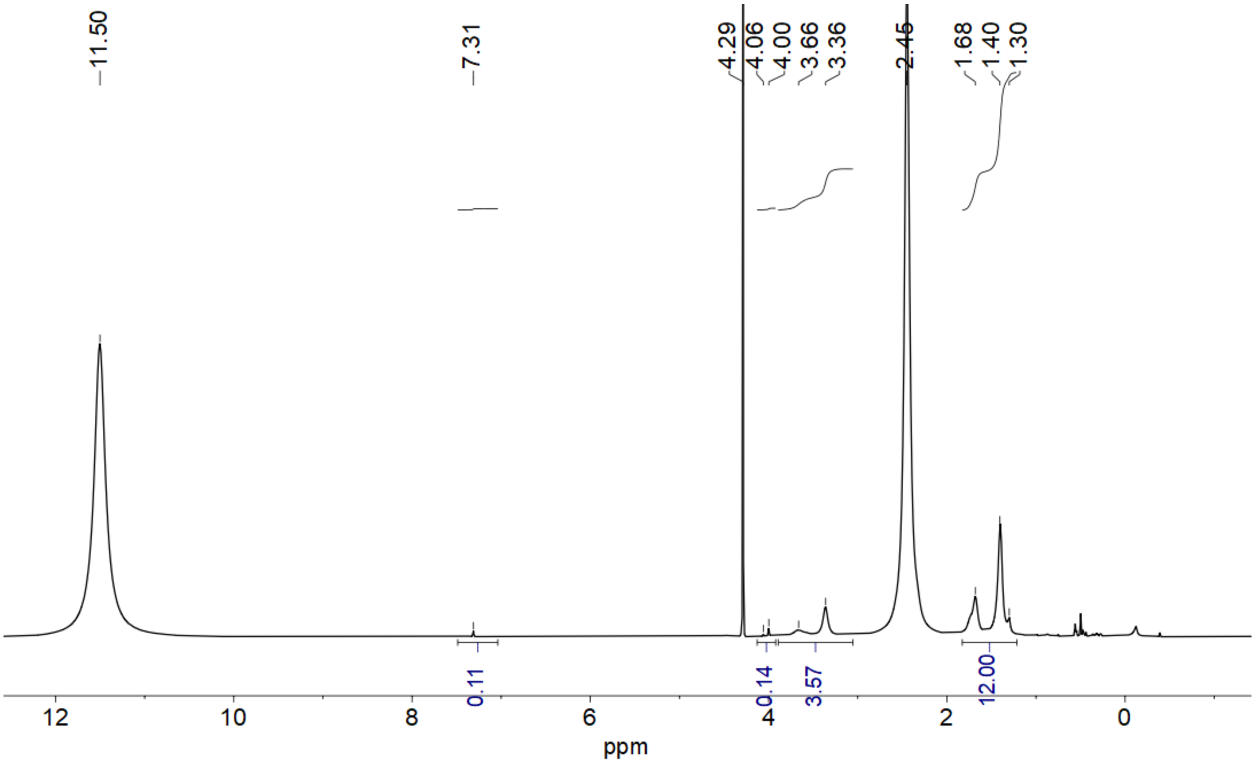


**Figure S31** $^{1}H$ NMR spectrum (d-TFA, 500 MHz, 298 K) of Entry 11. Signal at $\delta_H$ 2.45 corresponds to acetone.

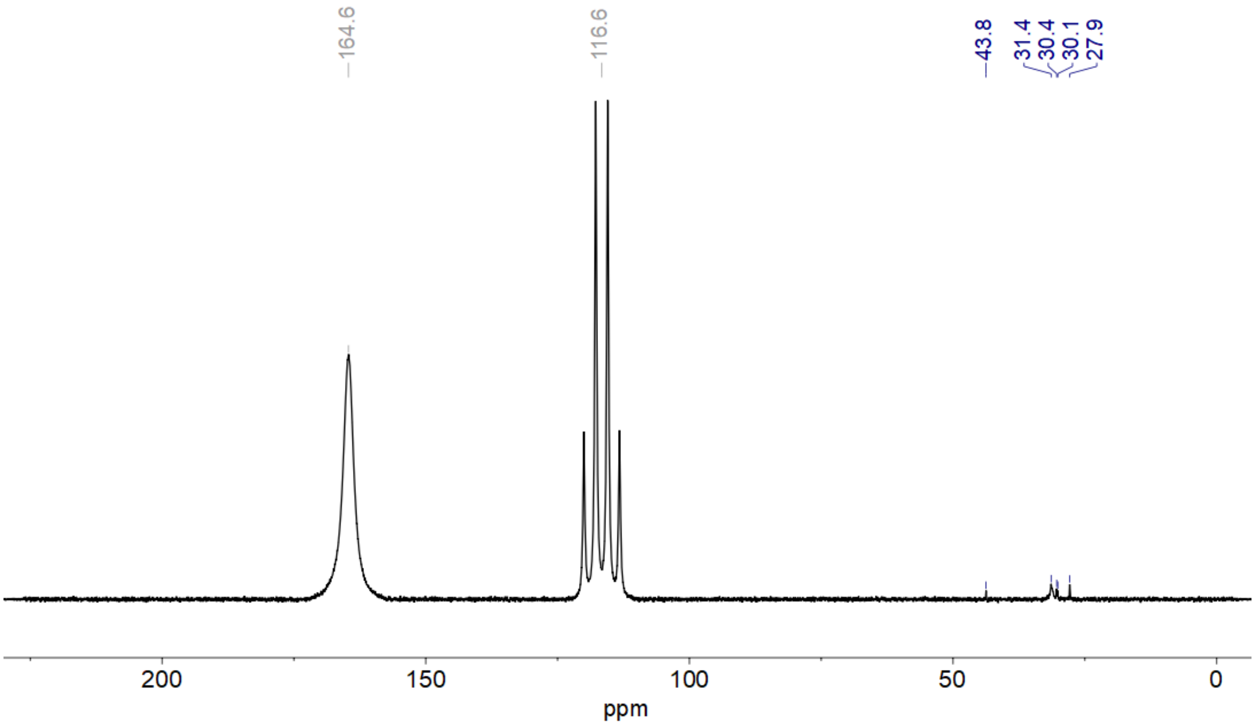


**Figure S32** $^{13}C\{^{1}H\}$ NMR spectrum (d-TFA, 126 MHz, 298 K) of Entry 11. Signal at $\delta_C$ 165.6 corresponds to acetone.

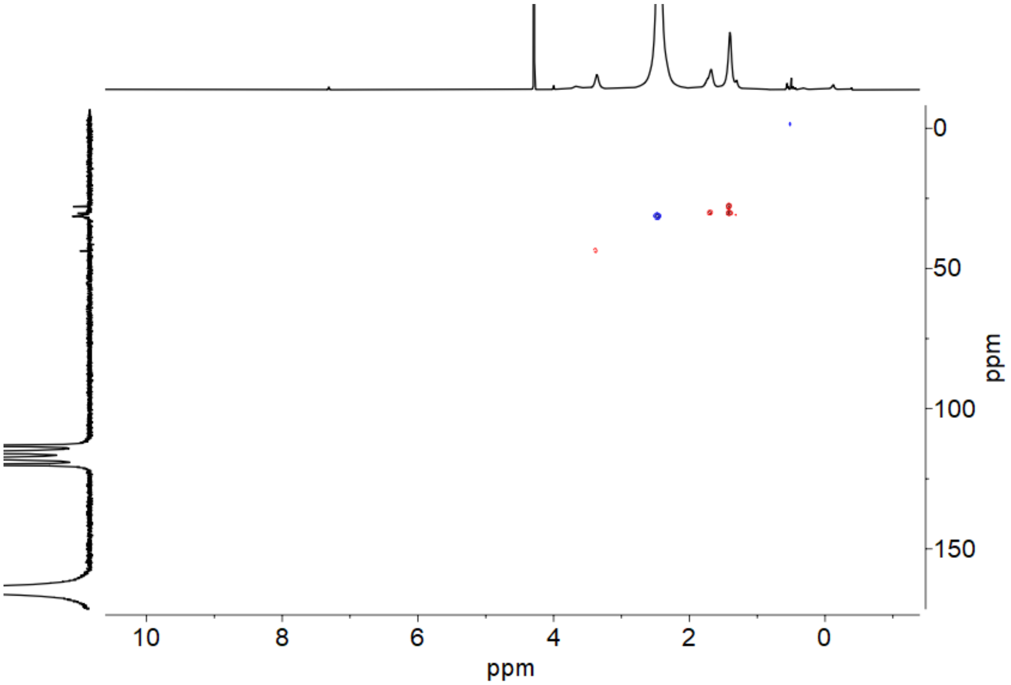


**Figure S33** $^{1}H$, $^{13}C$-HSQC NMR spectrum (d-TFA, 500-126 MHz, 298 K) of Entry 11.

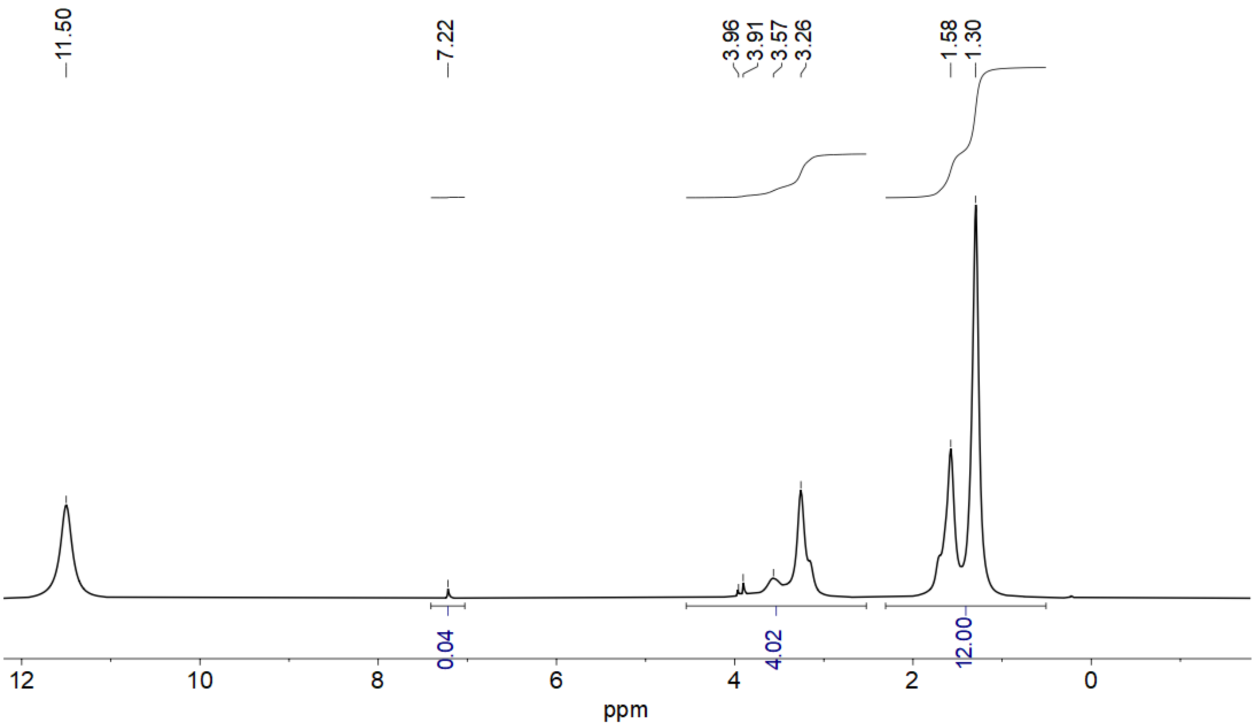


**Figure S34** $^{1}H$ NMR spectrum (d-TFA, 500 MHz, 298 K) of Entry 12.

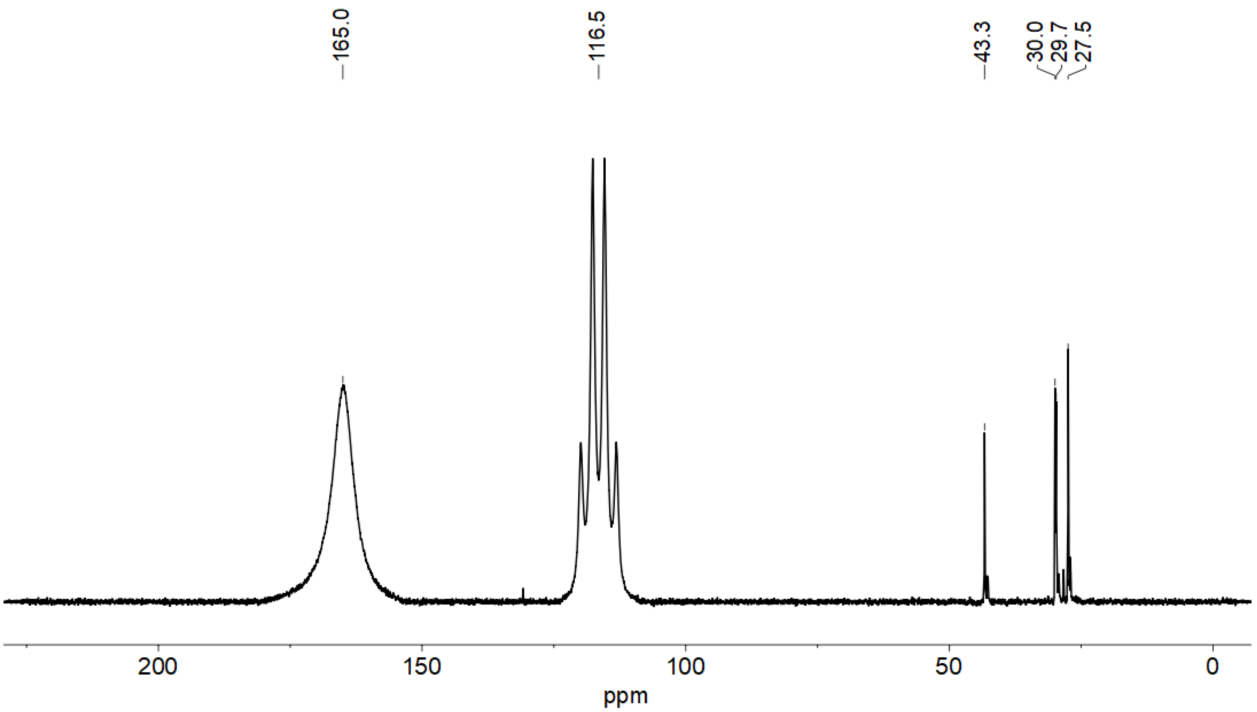


**Figure S35** $^{13}C\{^{1}H\}$ NMR spectrum (d-TFA, 126 MHz, 298 K) of Entry 12.

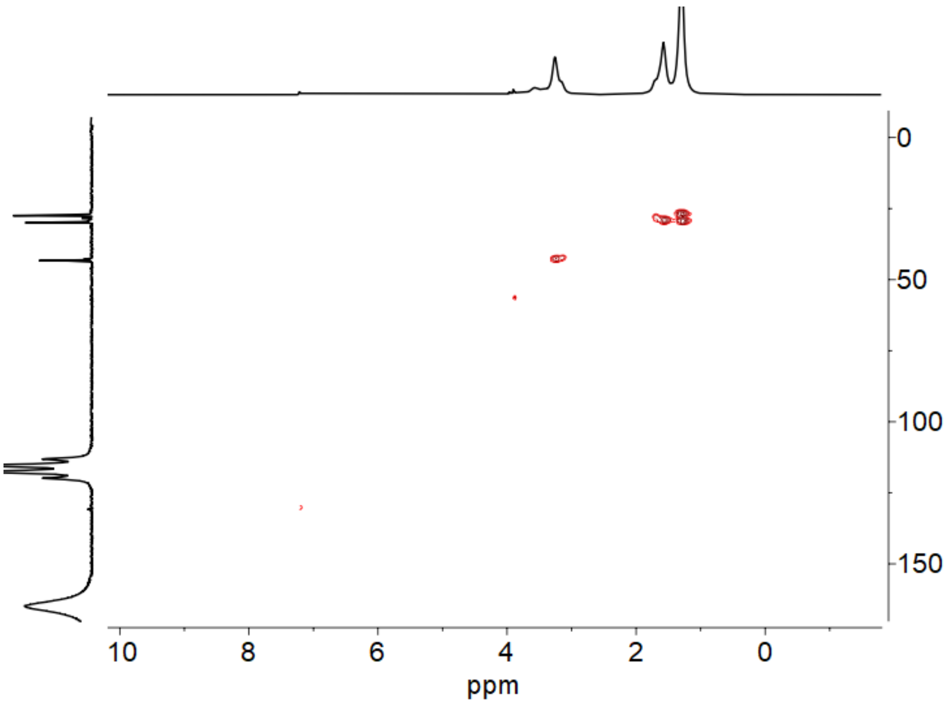


**Figure S36** $^{1}H$, $^{13}C$-HSQC NMR spectrum (*d*-TFA, 500-126 MHz, 298 K) of Entry 12.

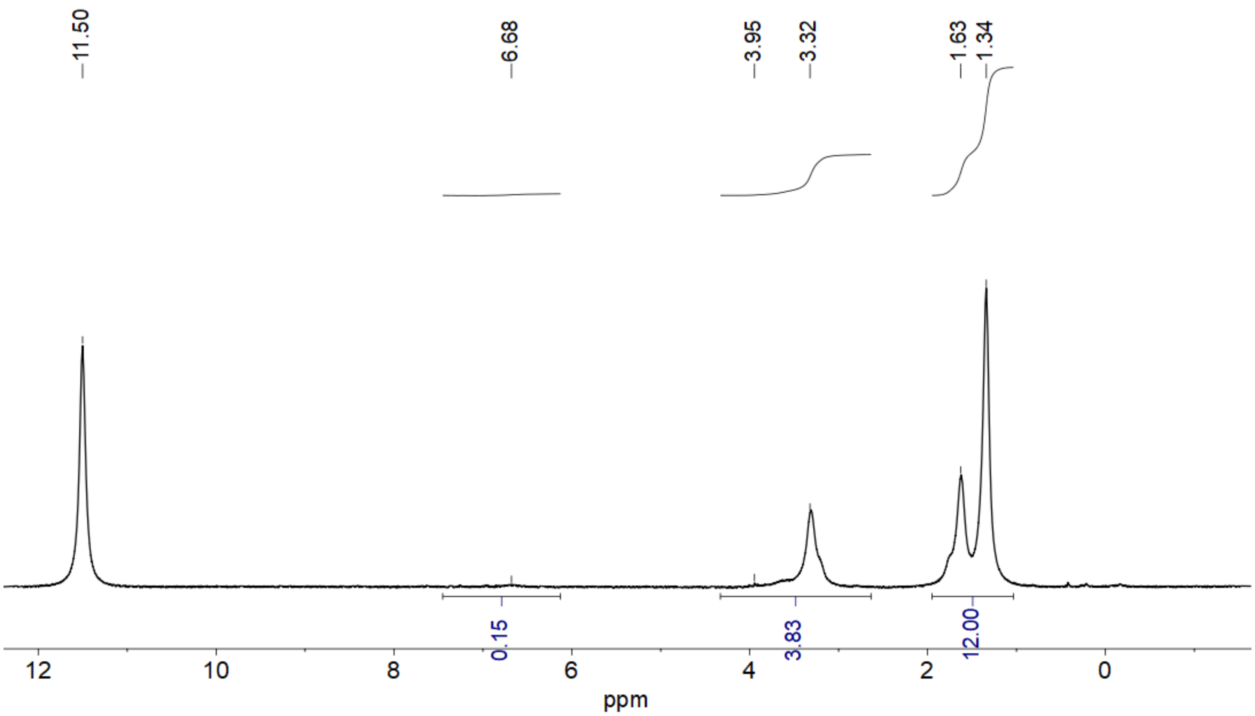


**Figure S37** $^{1}H$ NMR spectrum (*d*-TFA, 500 MHz, 298 K) of Entry 13.

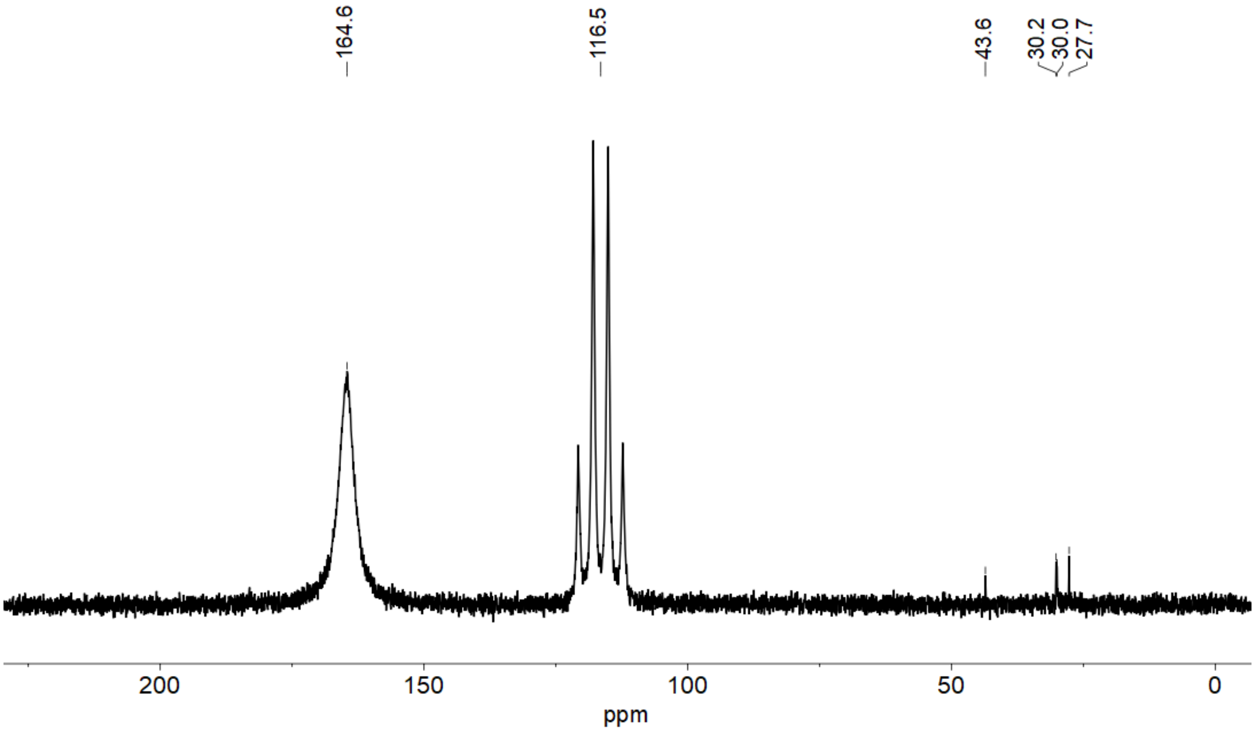


**Figure S38** $^{13}C\{^{1}H\}$ NMR spectrum (*d*-TFA, 126 MHz, 298 K) of Entry 13.

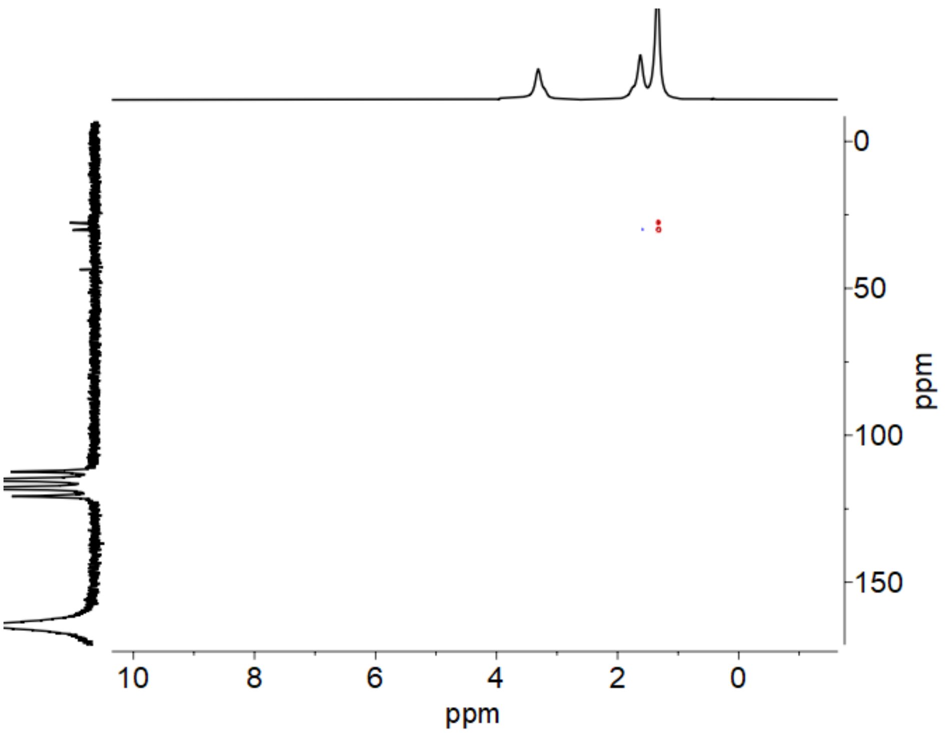

**Figure S39** $^{1}H$, $^{13}C$-HSQC NMR spectrum (*d*-TFA, 500-126 MHz, 298 K) of Entry 13.

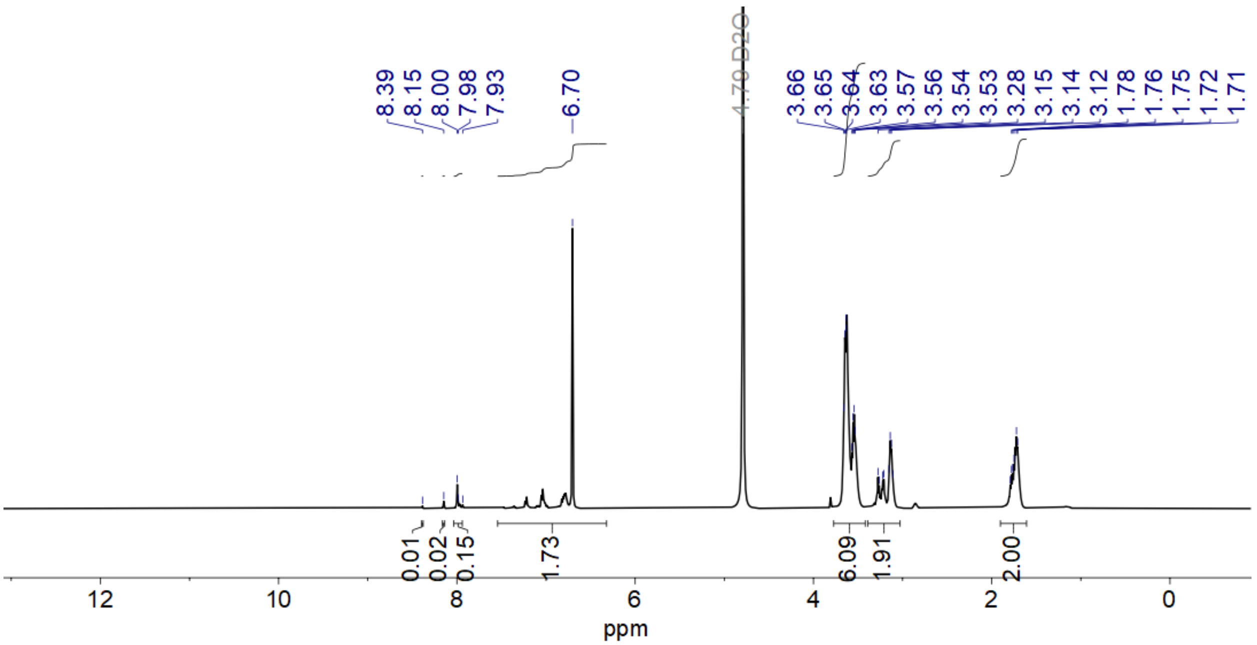

**Figure S40** $^{1}H$ NMR spectrum ($D_2O$, 500 MHz, 298 K) of Entry 14.

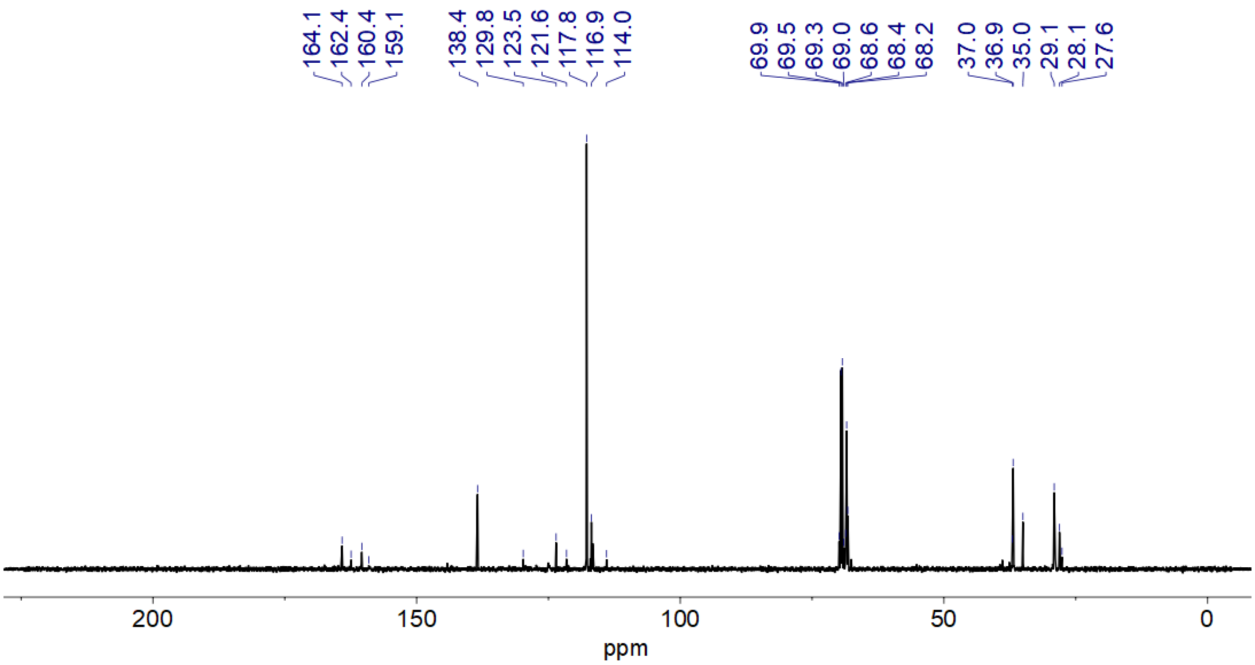


**Figure S41** $^{13}C\{^1H\}$ NMR spectrum ($D_2O$, 126 MHz, 298 K) of Entry 14.

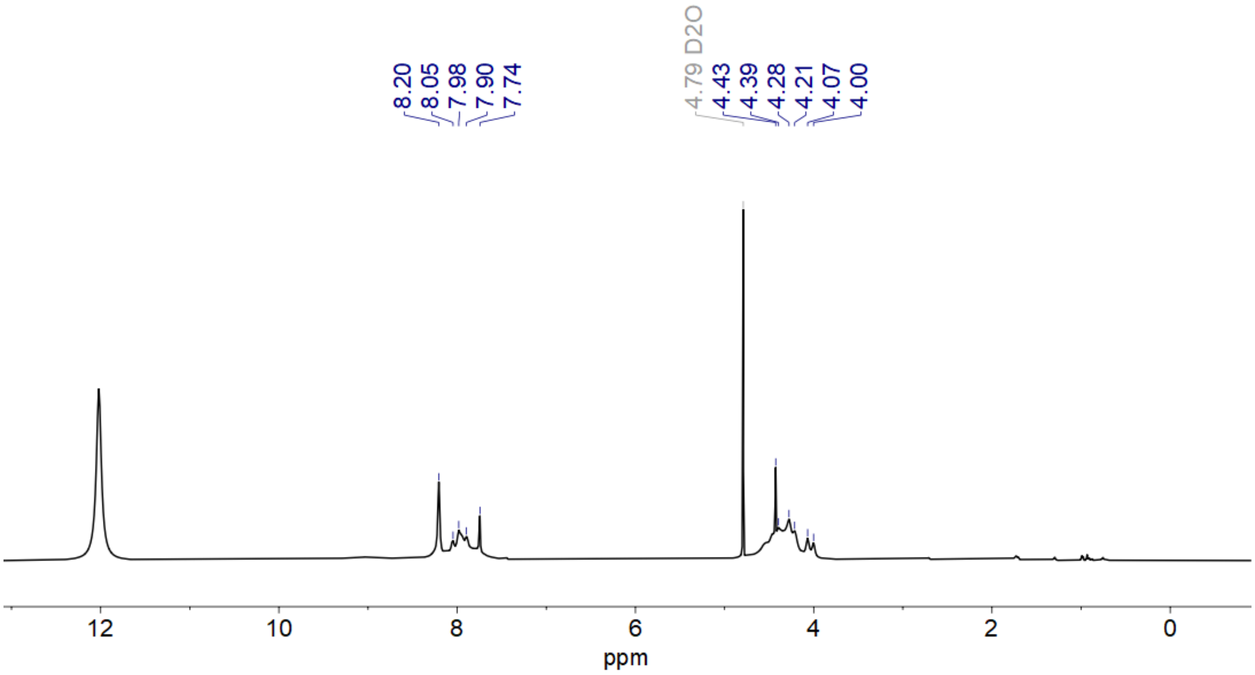


**Figure S42** $^1H$ NMR spectrum (d-TFA with $D_2O$ locking capillary, 500 MHz, 298 K) of Entry 15.

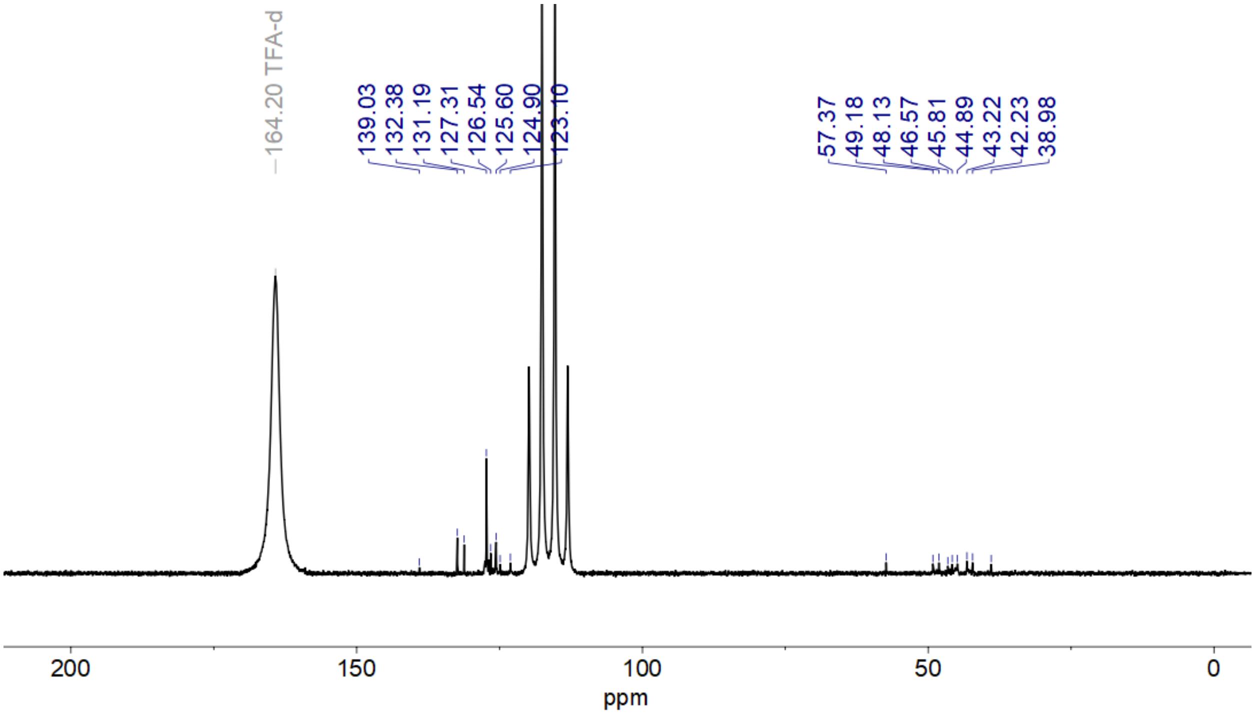


**Figure S43** $^{13}C\{^{1}H\}$ NMR spectrum ($D_2O$, 126 MHz, 298 K) of Entry 15.

## 8. IR spectra of the isolated polyureas

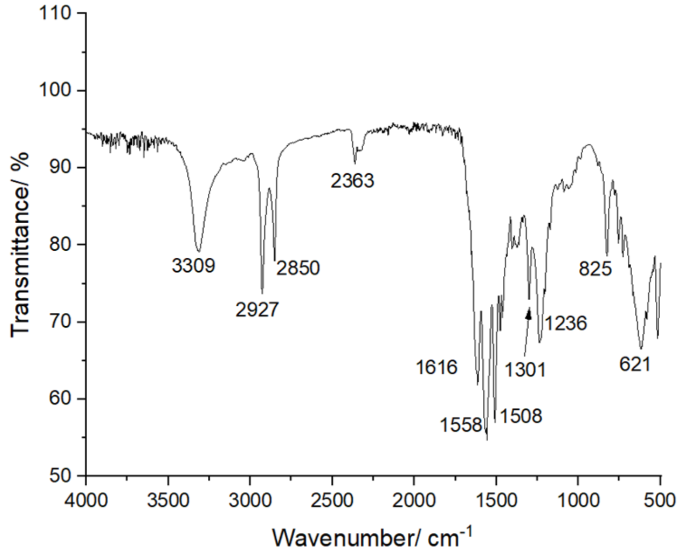


**Figure S44** IR spectrum of Entry 1.

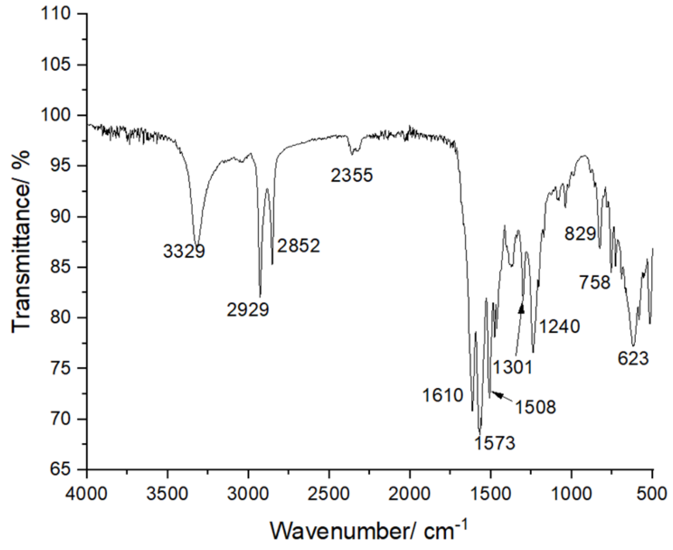


**Figure S45** IR spectrum Entry 2.

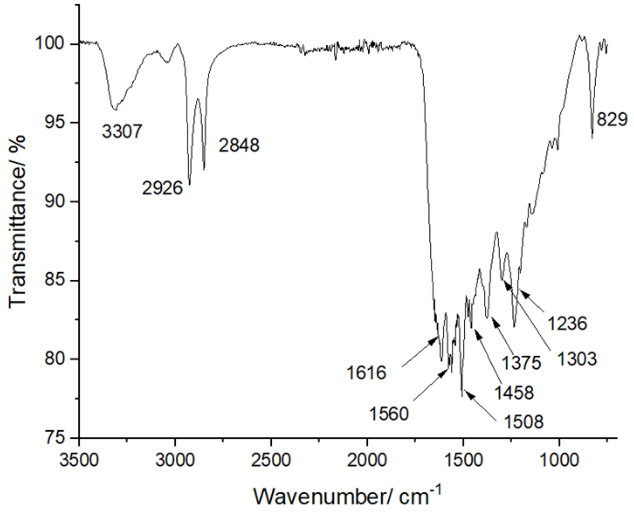


**Figure S46** IR spectrum of Entry 3.

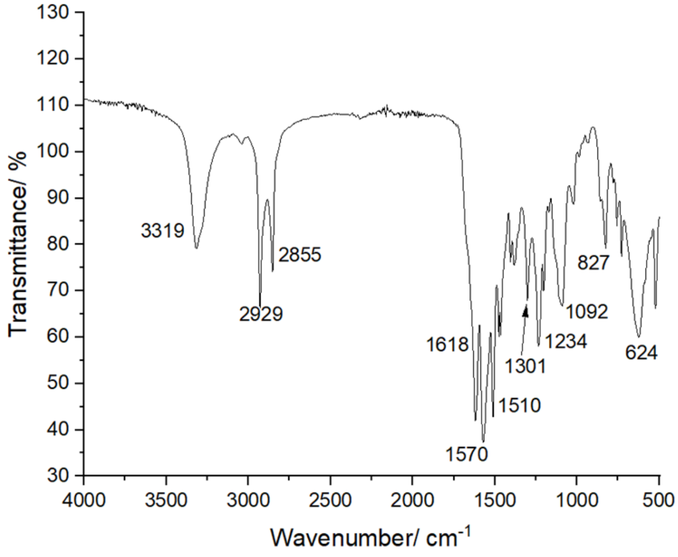


**Figure S47** IR spectrum of Entry 4.

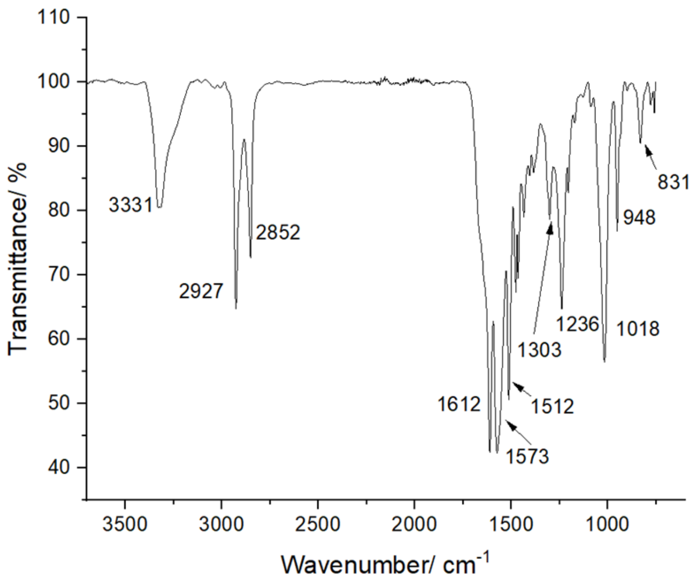


**Figure S48** IR spectrum of Entry 5.

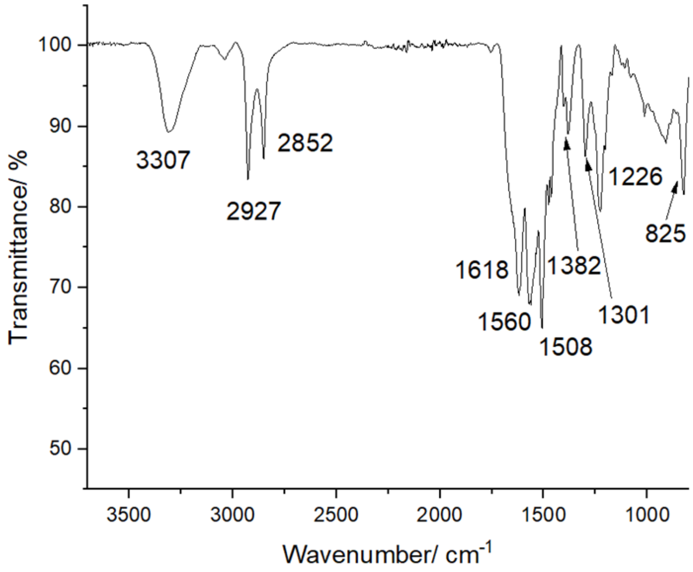


**Figure S49** IR spectrum of Entry 6.

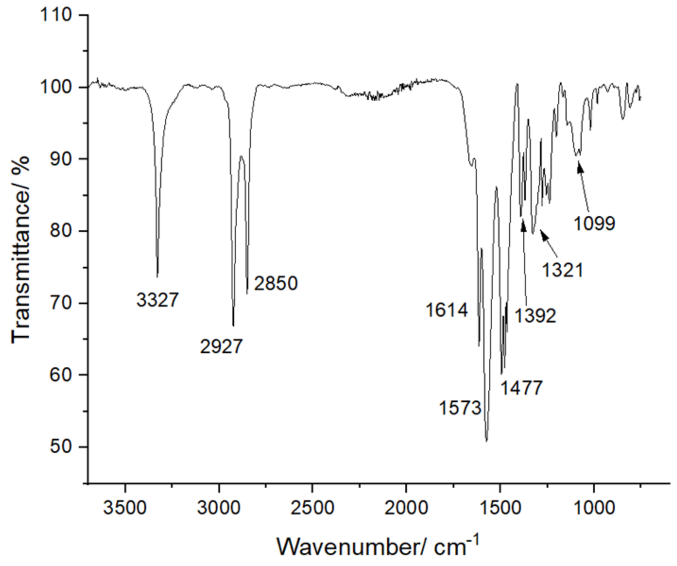


**Figure S50** IR spectrum of Entry 7.

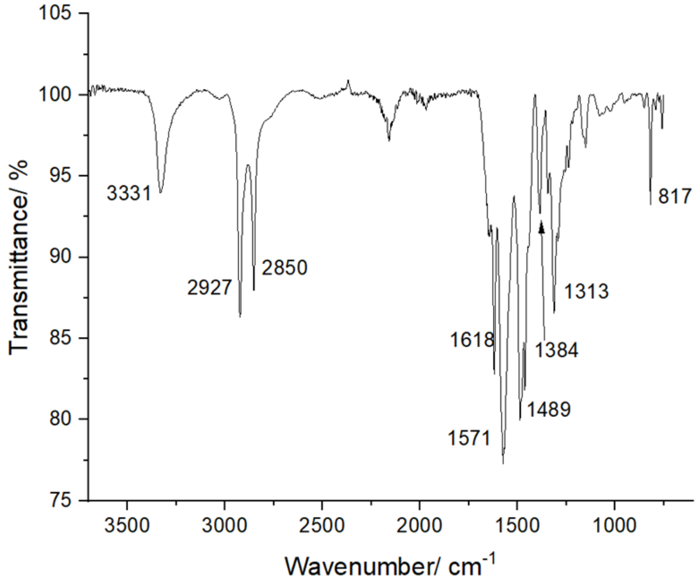


**Figure S51** IR spectrum of Entry 8.

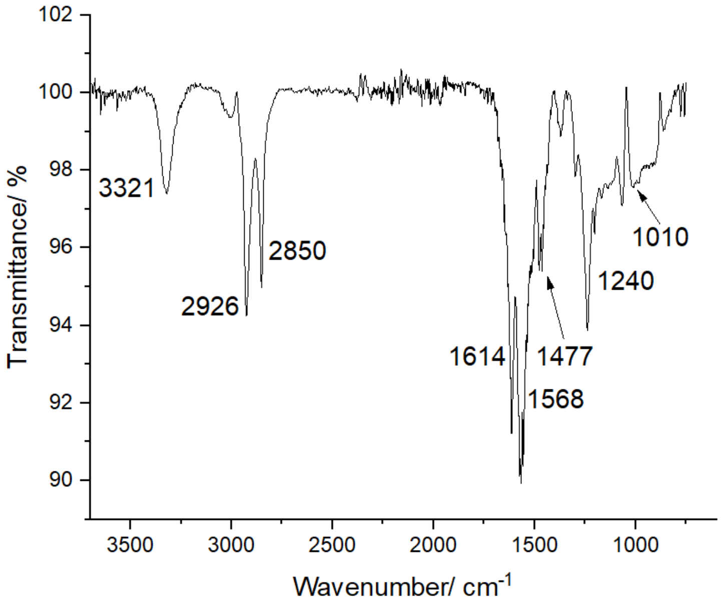


**Figure S52** IR spectrum of Entry 9.

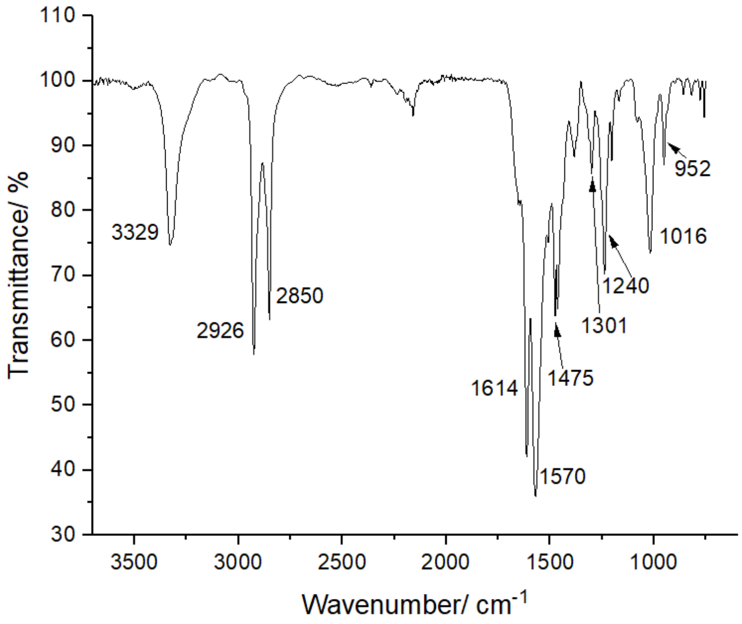


**Figure S53** IR spectrum of Entry 10.

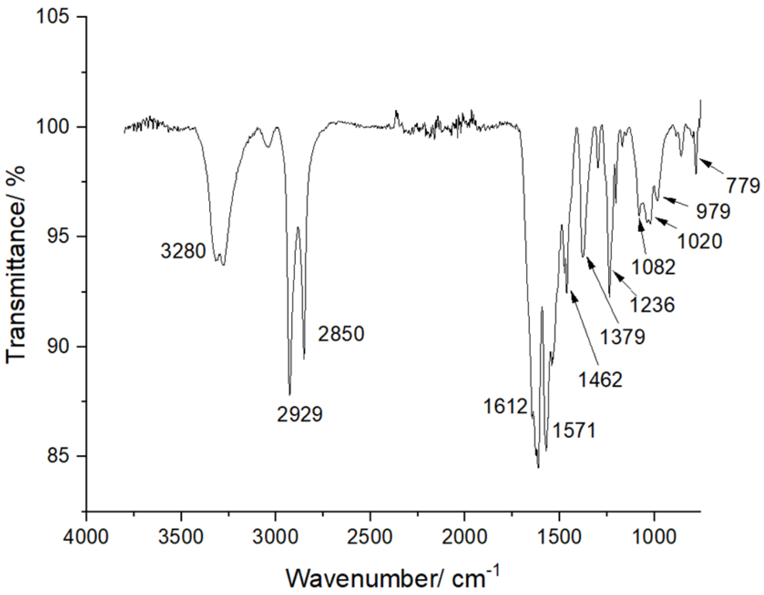


**Figure S54** IR spectrum of Entry 11.

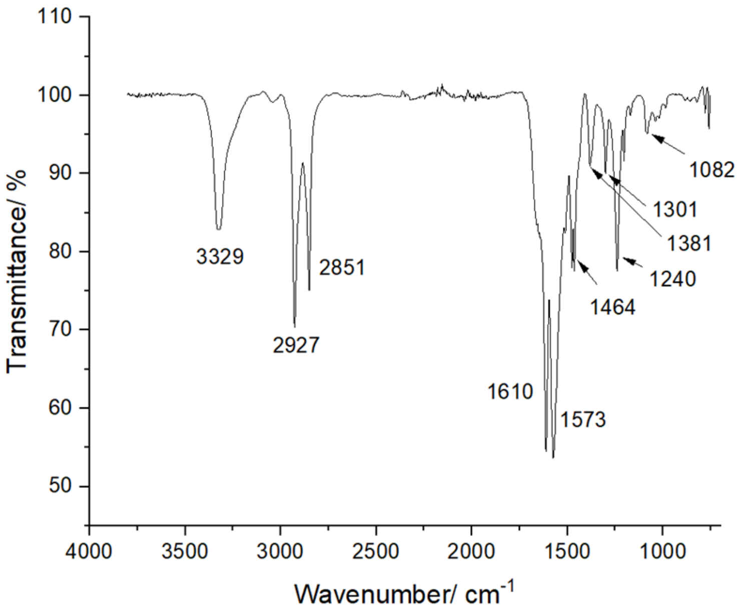


**Figure S55** IR spectrum of Entry 12.

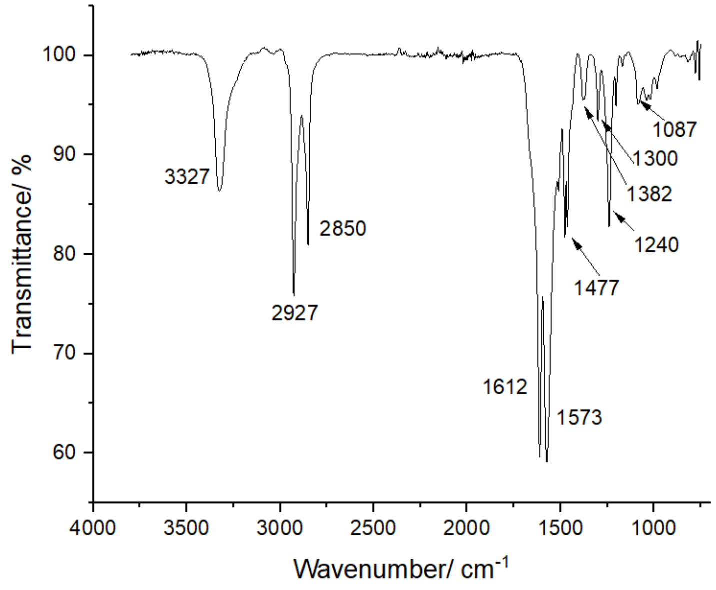


**Figure S56** IR spectrum of Entry 13.

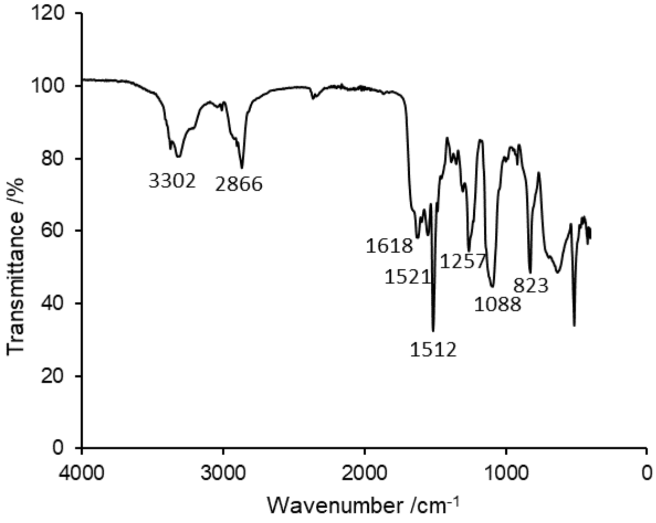


**Figure S57** IR spectrum of Entry 14.

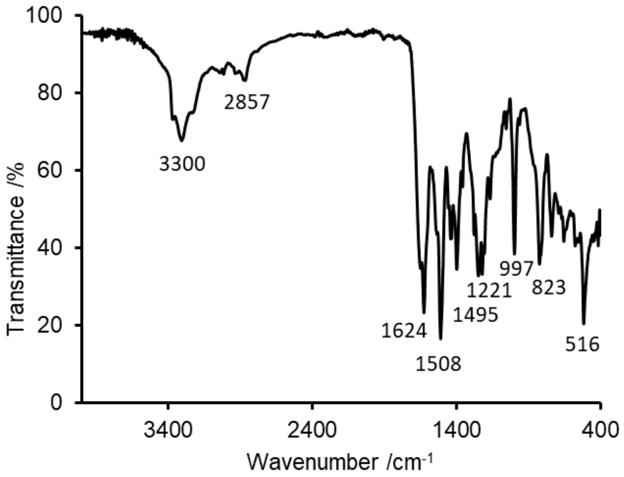


**Figure S58** IR spectrum of Entry 15.

# 9. Polymers observed via MALDI FT-ICR MS

Polyurea

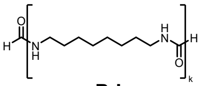

**P-k**

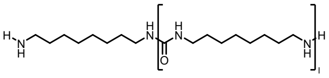

**P-i**

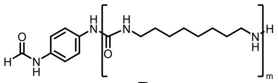

**P-m**

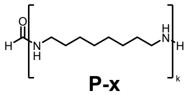

**P-x**

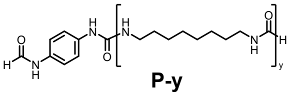

**P-y**

Alternating co-polymer

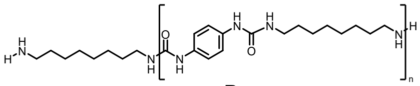

**P-n**

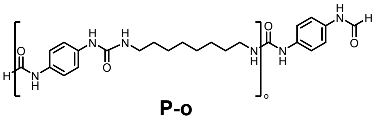

**P-o**

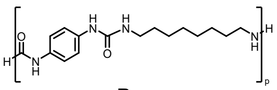

**P-p**

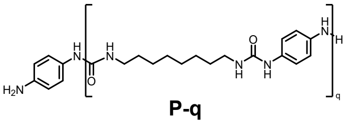

**P-q**

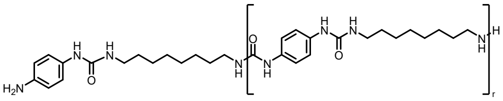

**P-r**

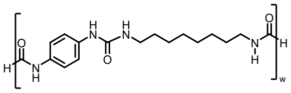

**P-w**

Block co-polymer

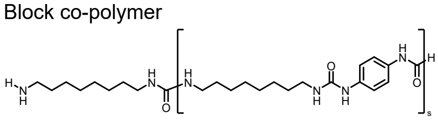

**P-s**

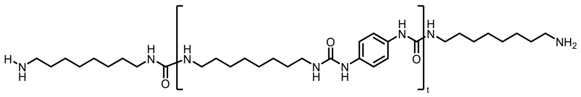

**P-t**

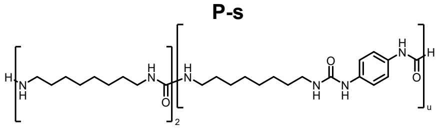

**P-u**

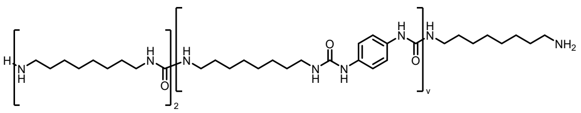

**P-v**

**Table S2** The oligomers observed via MALDI-FT-ICR MS experiments for the polymers synthesised from the reaction of diformamide with diamine in the presence of **1** (entry numbers are the same as described in Table S1). N/O = not observed

| Entry | P-k | P-x | P-i | P-m | P-y | P-n | P-o | P-p | P-q | P-r | P-s | P-t | P-u | P-y | P-w |
|---|---|---|---|---|---|---|---|---|---|---|---|---|---|---|---|
| 1 | 3-9 | N/O | 1-4 | 1-10 | N/O | 1-5 | 1-4 | 1-5 | 1-5 | 1-5 | 1-4 | 1-4 | 1-2 | 1-3 | - |
| 2 | 1-12 | 1-13 | N/O | 1-10 | 1-10 | 1-6 | N/O | 1-5 | 1-5 | 1-5 | 1-4 | 1-4 | 1-2 | 1-3 | - |
| 3 | 1-12 | N/O | N/O | 1-9 | 1-9 | 1-3 | N/O | N/O | N/O | N/O | 1-2 | N/O | N/O | N/O | - |
| 4 | 1-9 | N/O | N/O | 1-9 | 1-10 | 1-5 | N/O | 1-6 | 2-3 | 1-2 | 1-4 | N/O | 1-2 | N/O | - |
| 6 | 1-10 | N/O | N/O | 1-9 | 1-9 | N/O | N/O | 1-3 | 1-2 | 1-3 | 1-4 | N/O | 1-3 | N/O | - |
| 7 | 1-12 | 2-12 | 1-8 | - | - | - | - | - | - | - | - | - | - | - | - |
| 8 | 1-13 | 2-12 | 1-10 | - | - | - | - | - | - | - | - | - | - | - | - |
| 9 | 1-14 | 2-15 | 1-12 | - | - | - | - | - | - | - | - | - | - | - | - |
| 14[a] | - | - | - | - | 3 | 2-3 | 2 | 3 | 1 | - | - | - | 2 | - | 1 |

[a] The MALDI-TOF of Entry 14 was carried out on a sonicated solution (5 min) of polymer (1 mg/mL) in 1:1 MeOH:$H_2O$ using α-cyano-4-hydroxycinnamic acid (CHCA) matrix in the presence of NaI (1 mg/mL). All other samples were prepared as 10 mg/mL solutions in 50:50 acetonitrile: 0.1% TFA using 2,5-dihydroxybenzoic acid or CHCA matrix.

## 10. Results of the MALDI-FT-ICR mass spectrometry measurements

We observe various polyureas (P-k, P-x, P-i, P-m, P-y, P-n, P-q, P-o, P-p, P-r, P-s, P-u, P-t and P-v) as described in Table S2. In the following (**Figure S*53*** - **Figure S*60***), the letters (k, x, i, m, y, n, q, r, s, u, t, and v) correspond to a specific polyurea and numbers correspond to the number of repeating units as described in the structure. For example, k=1 corresponds to polyurea P-k with one repeating unit in the following structure:

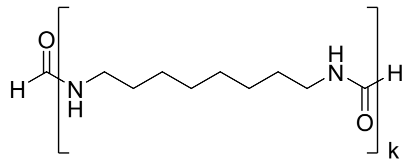


P-k

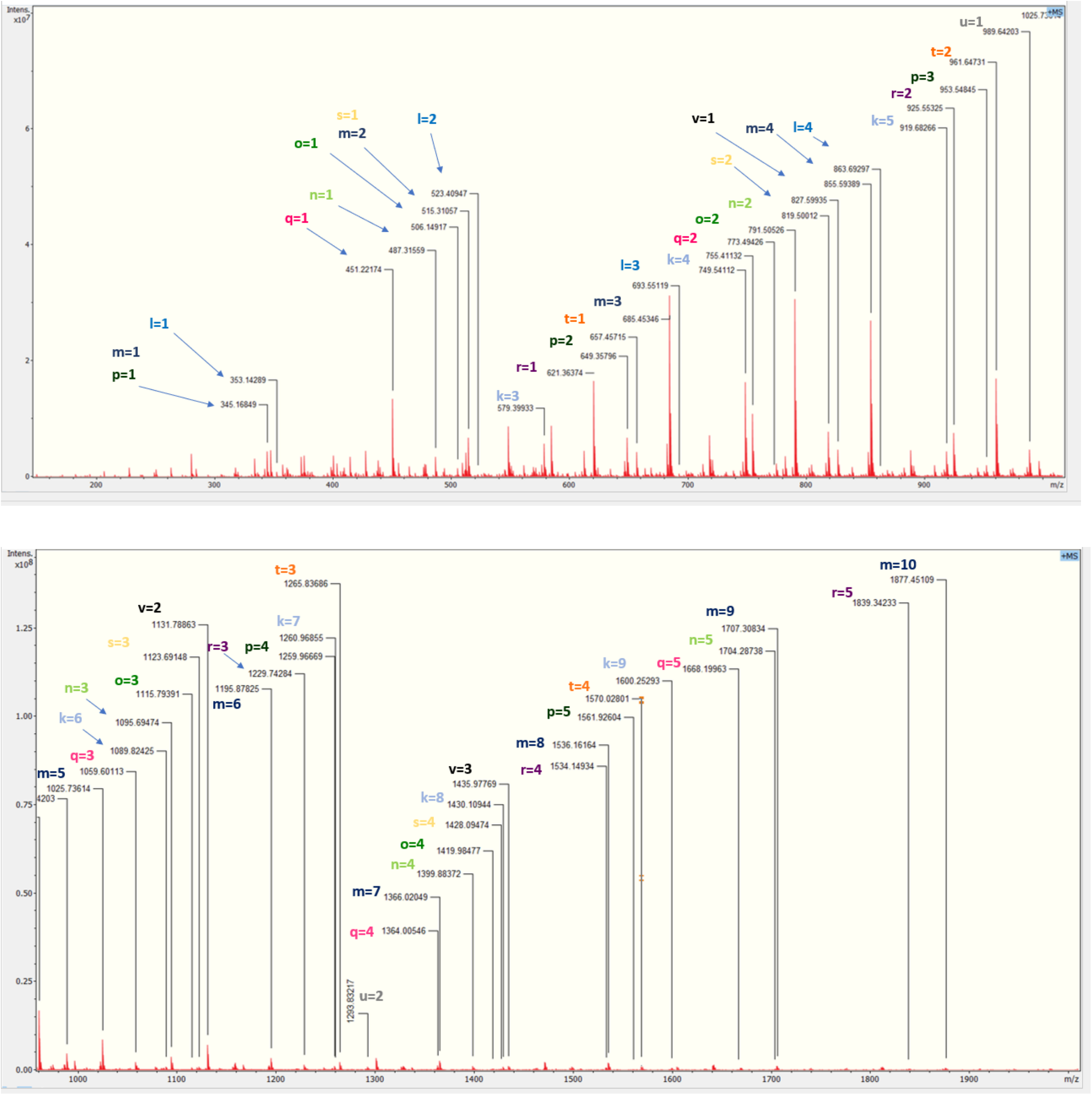


**Figure S59** MALDI-FT-ICR mass spectrometry result of the polyurea of Entry 1. The labelled peaks correspond to [Polyurea + K]$^+$.

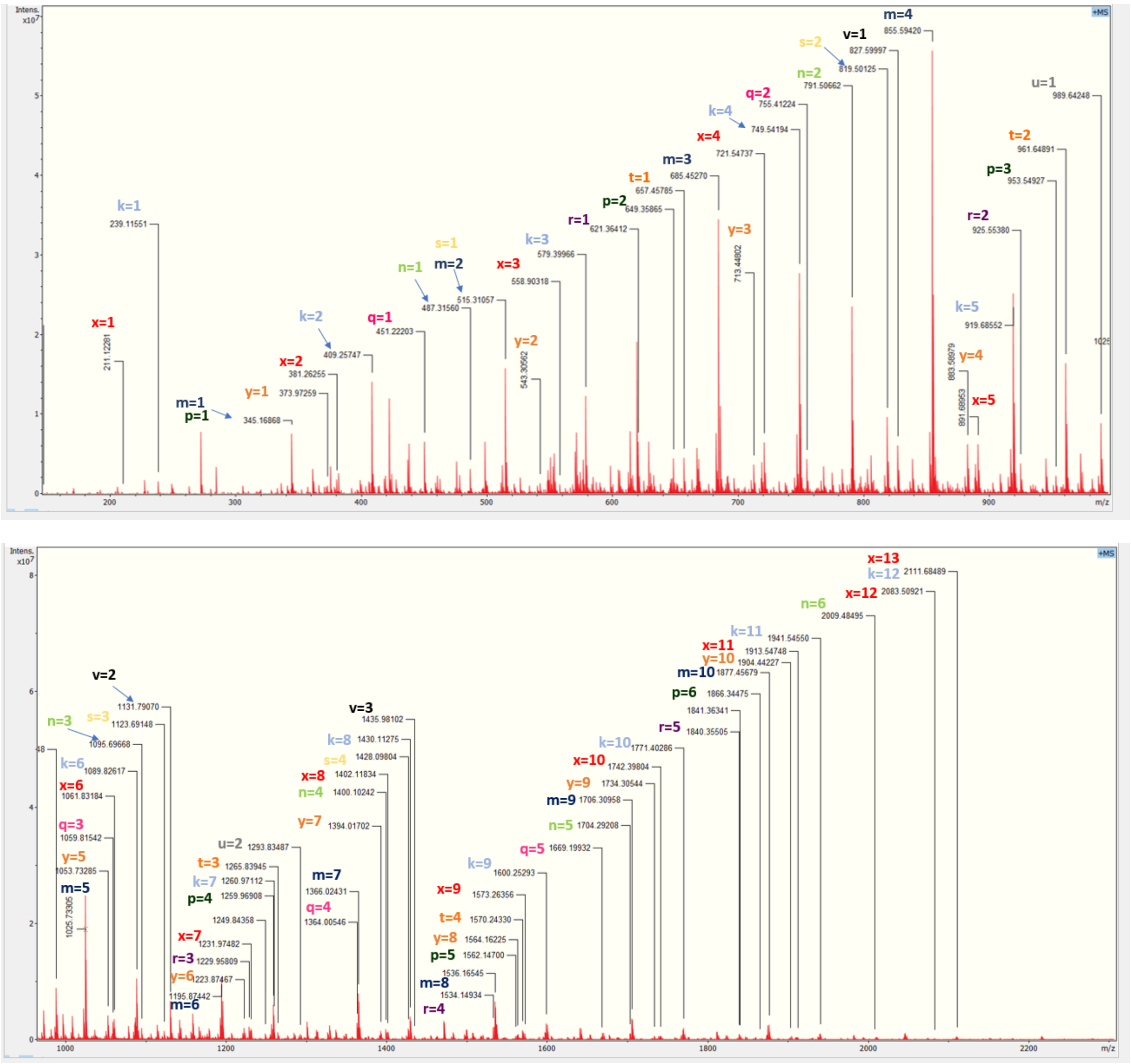

**Figure S60** MALDI-FT-ICR mass spectrometry result of the polyurea of Entry 2. The labelled peaks correspond to [Polyurea + K]$^+$.

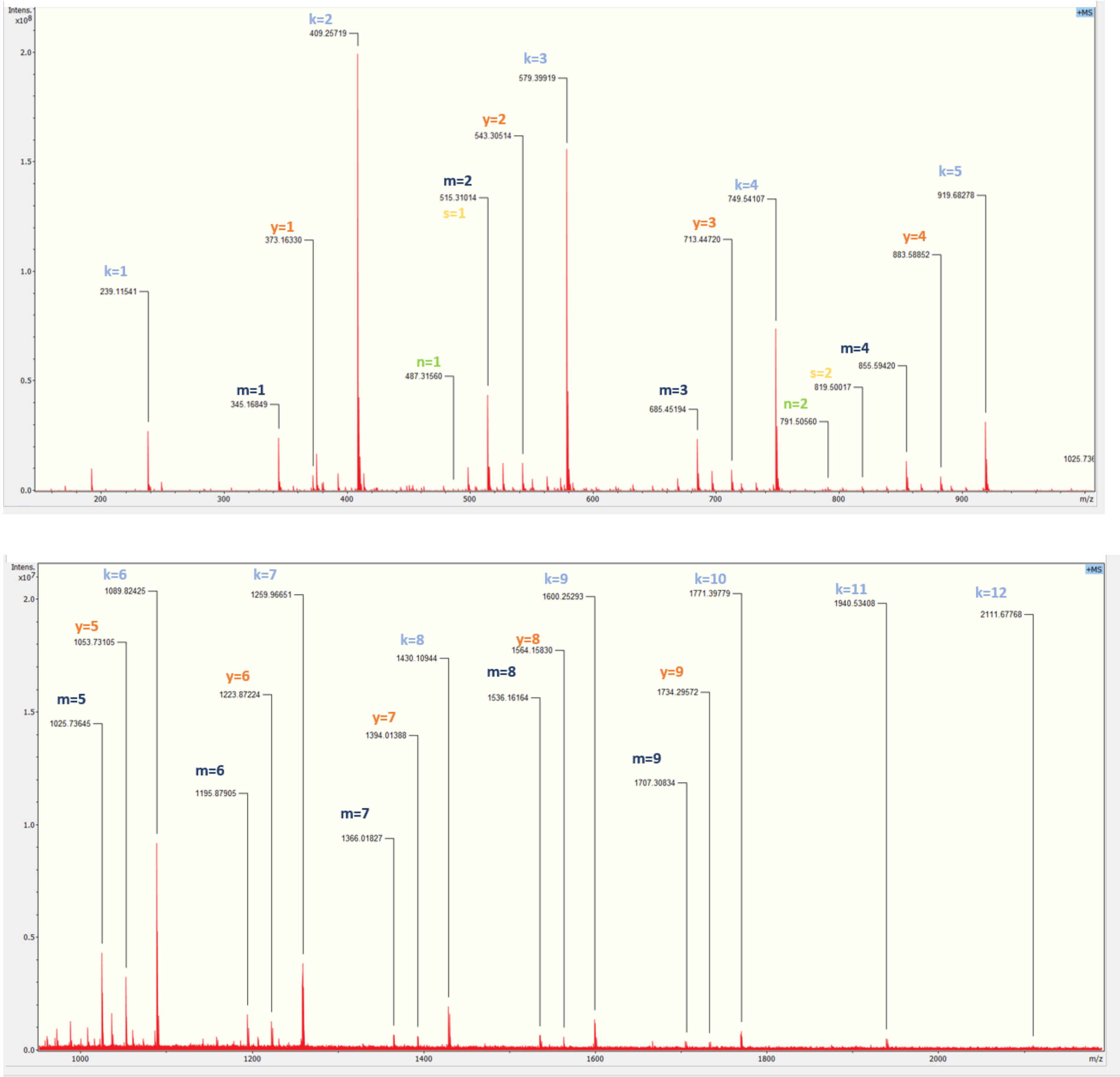


**Figure S61** MALDI-FT-ICR mass spectrometry result of the polyurea of Entry 3. The labelled peaks correspond to [Polyurea + K]$^+$.

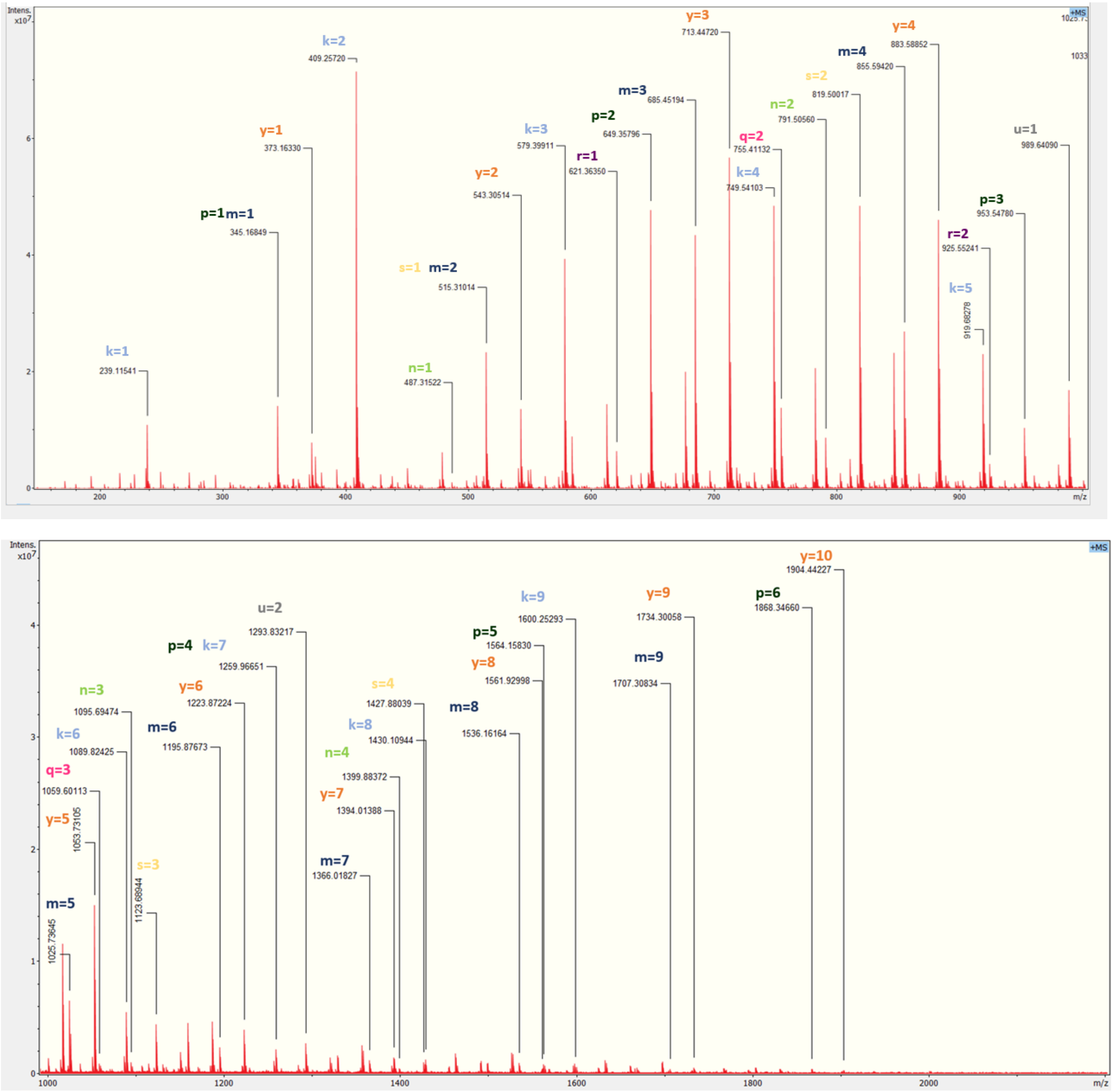


**Figure S62** MALDI-FT-ICR mass spectrometry result of the polyurea of Entry 4. The labelled peaks correspond to [Polyurea + K]$^+$.

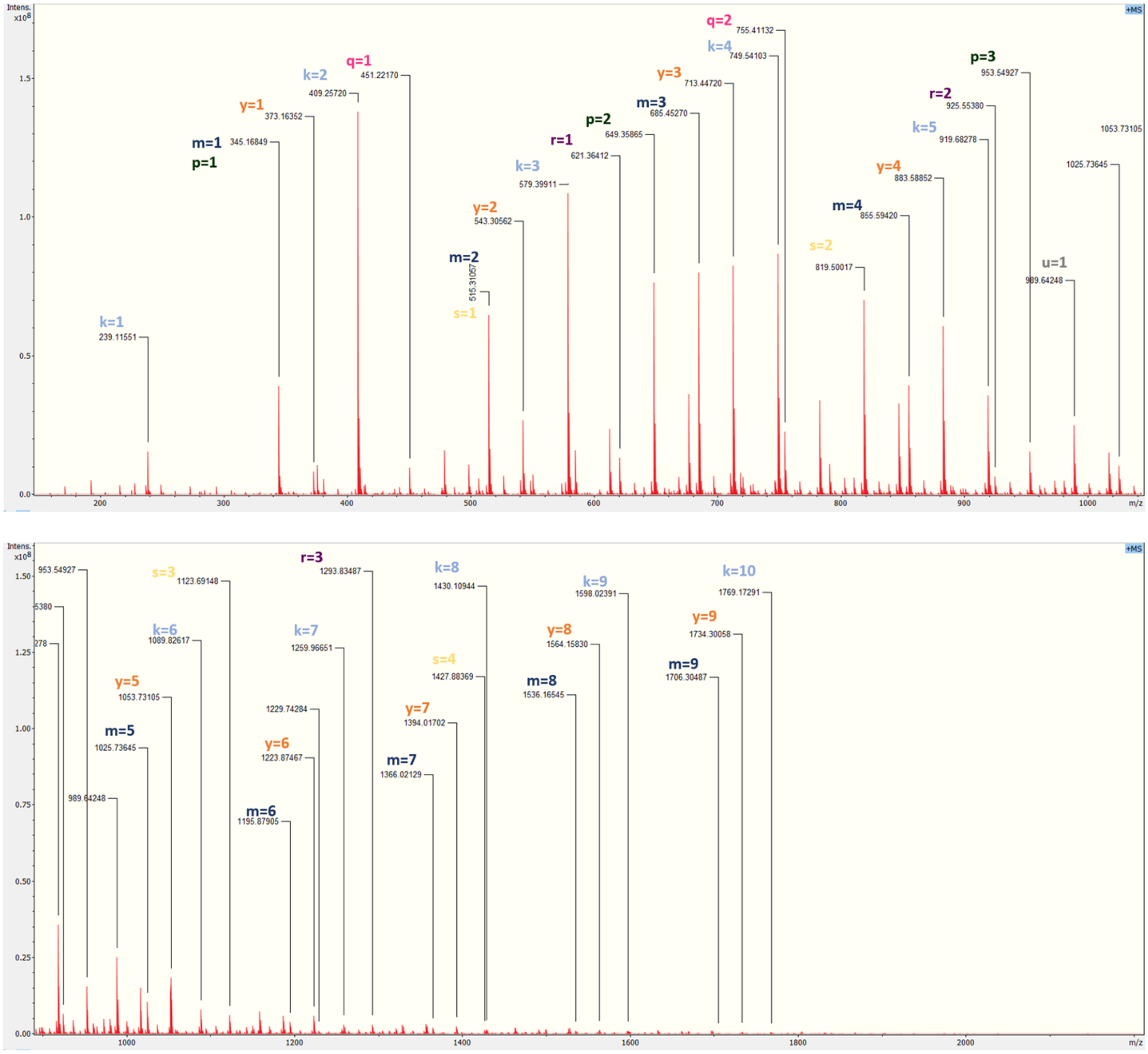


**Figure S63** MALDI-FT-ICR mass spectrometry result of the polyurea of Entry 6. The labelled peaks correspond to [Polyurea + K]$^+$.

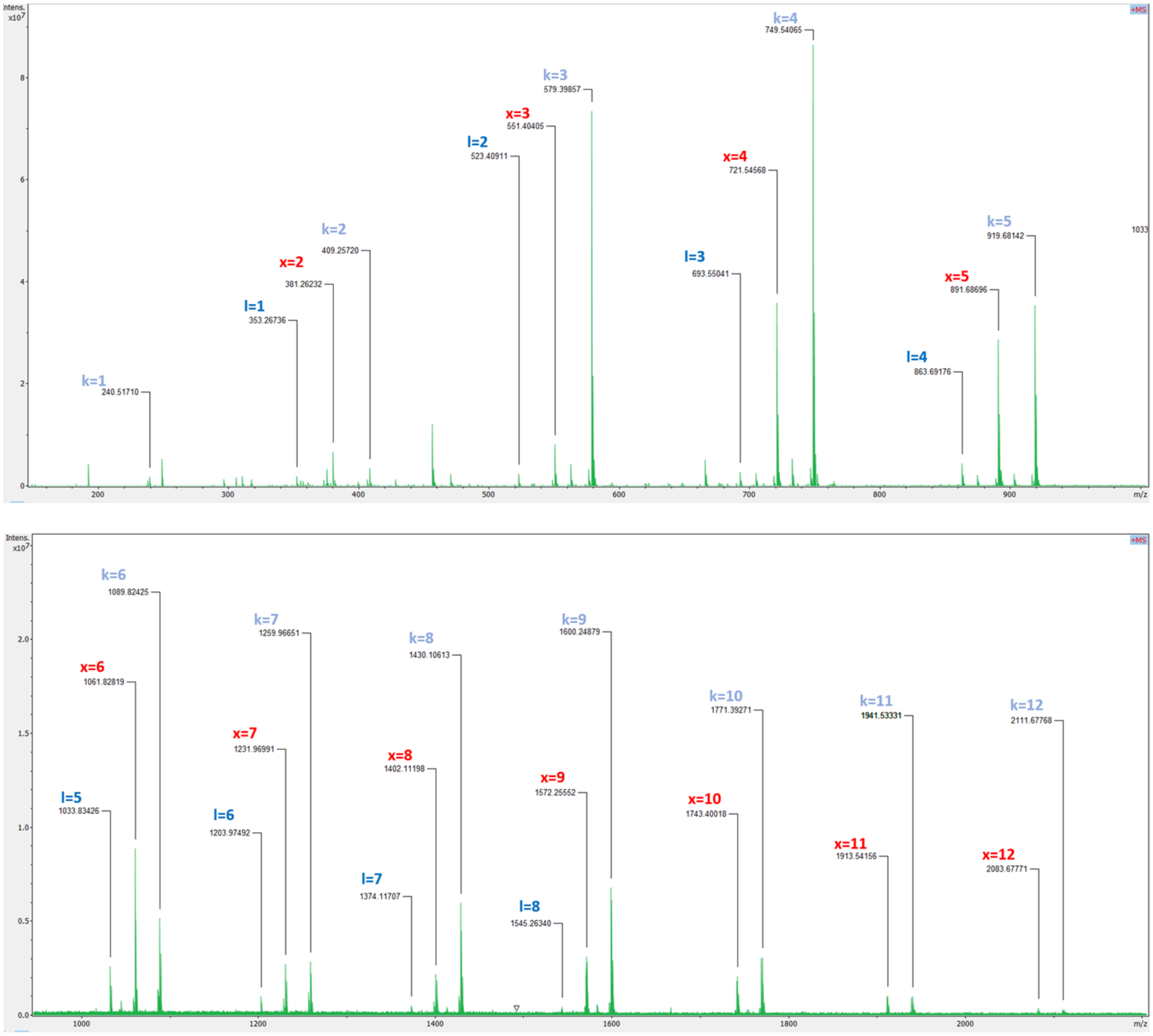


**Figure S64** MALDI-FT-ICR mass spectrometry result of the polyurea Entry 7. The labelled peaks correspond to [Polyurea + K]$^+$.

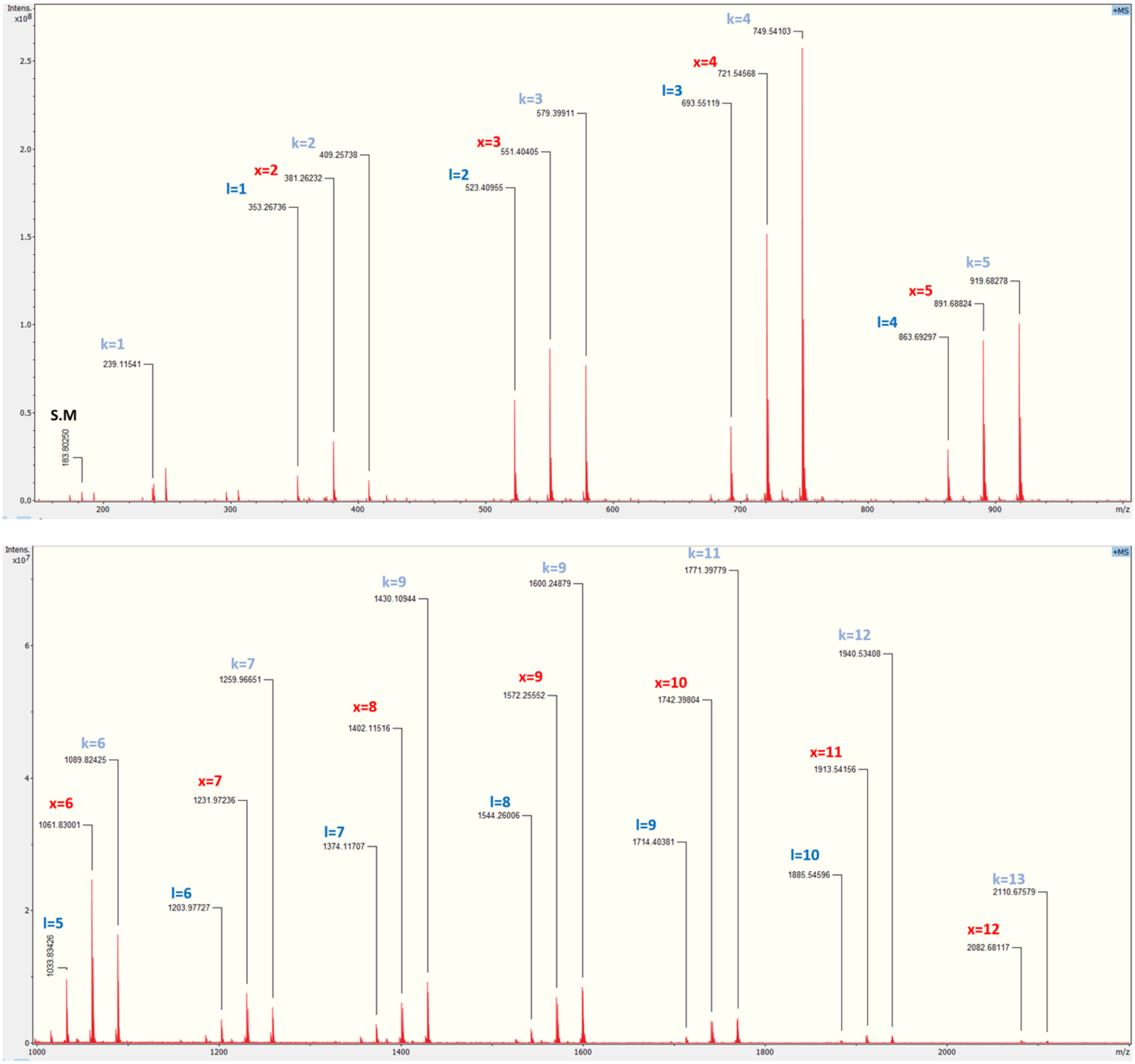


**Figure S65** MALDI-FT-ICR mass spectrometry result of the polyurea of Entry 8. The labelled peaks correspond to [Polyurea + K]$^+$.

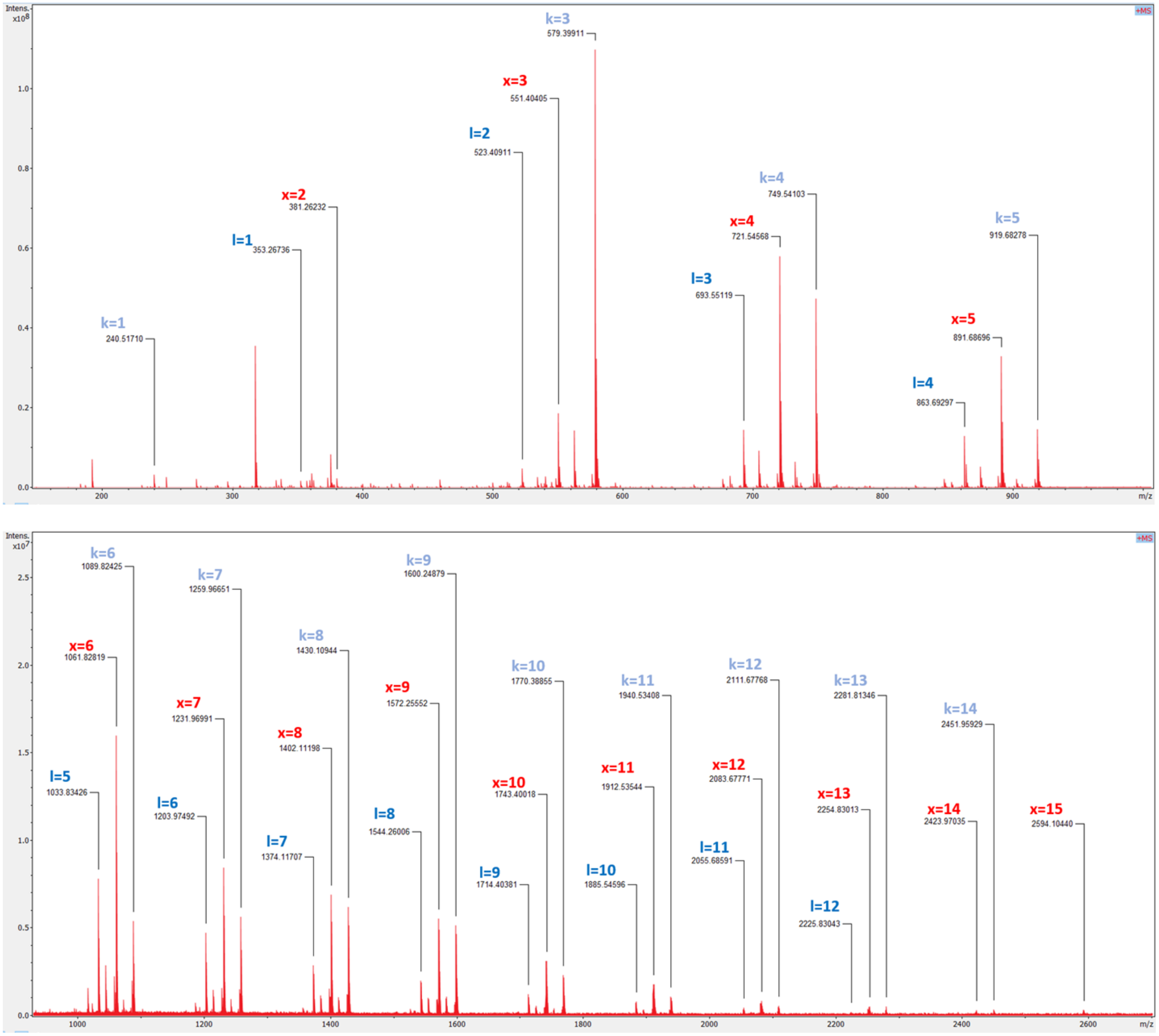


**Figure S66** MALDI-FT-ICR mass spectrometry result of the polyurea of Entry 9. The labelled peaks correspond to [Polyurea + K]$^+$

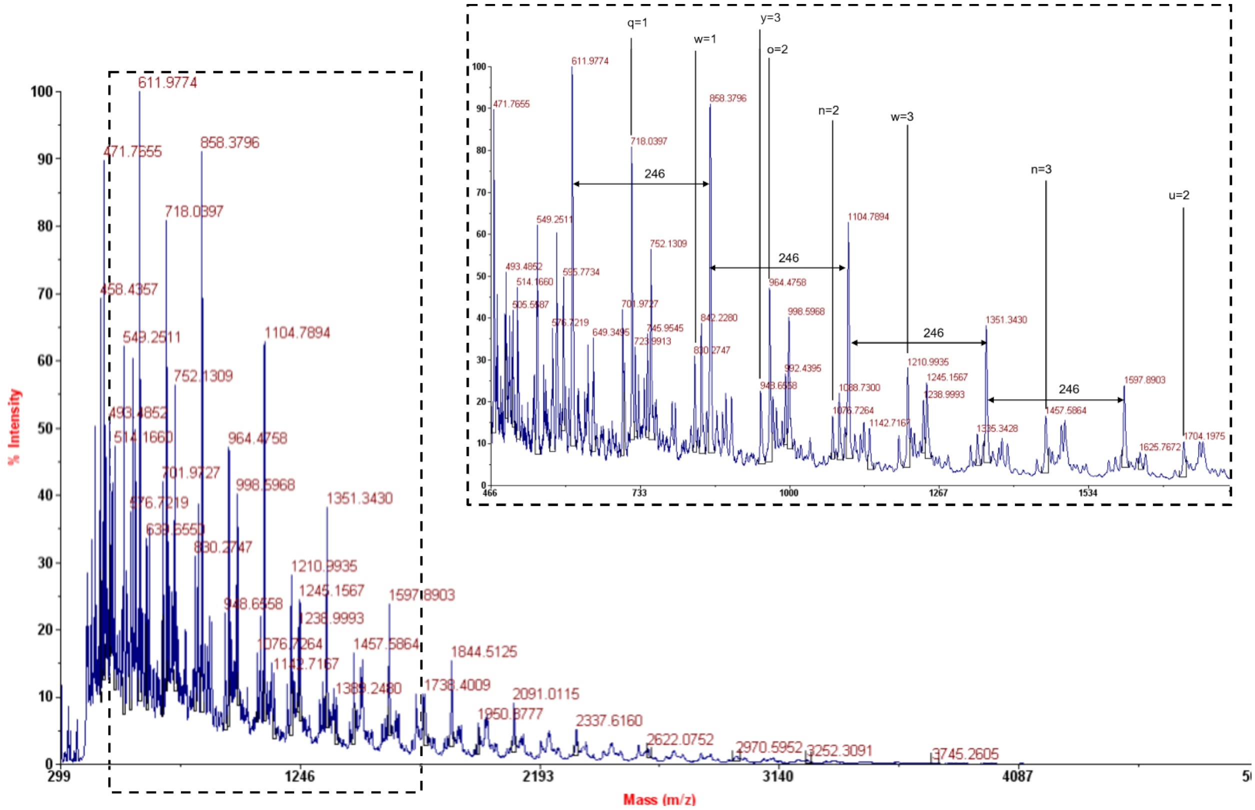

**Figure S67** MALDI-TOF spectrum obtained from sample corresponding to Table S1; Entry 14. The labelled peaks correspond to [urea + K]$^+$

## 11. TGA, DSC and GPC data

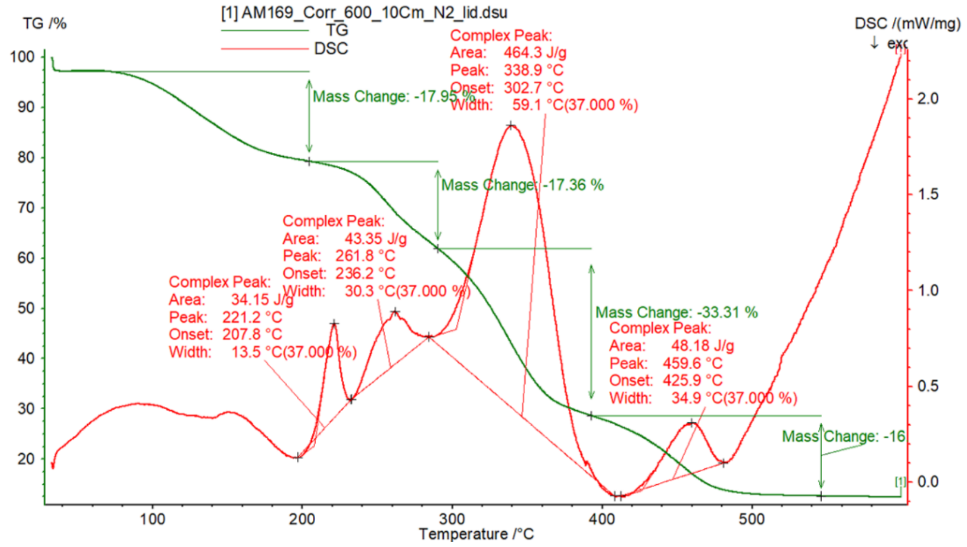


**Figure S68** TGA and DSC results for Entry 1.

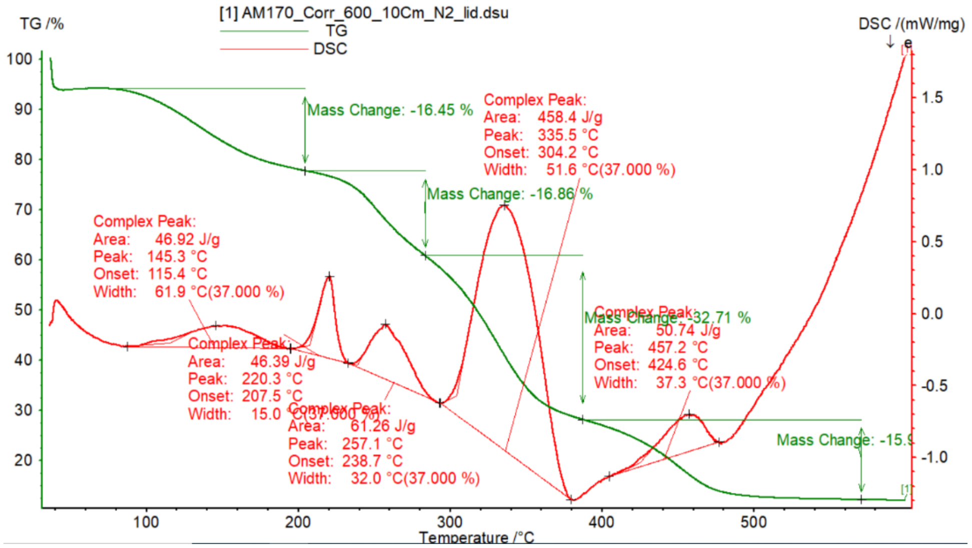


**Figure S69** TGA and DSC results for Entry 2.

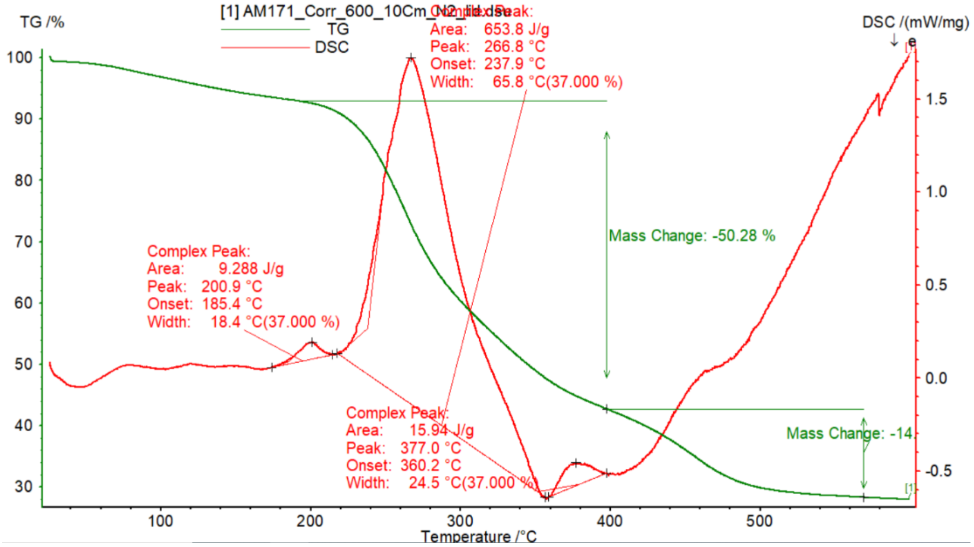


**Figure S70** TGA and DSC results for Entry 3.

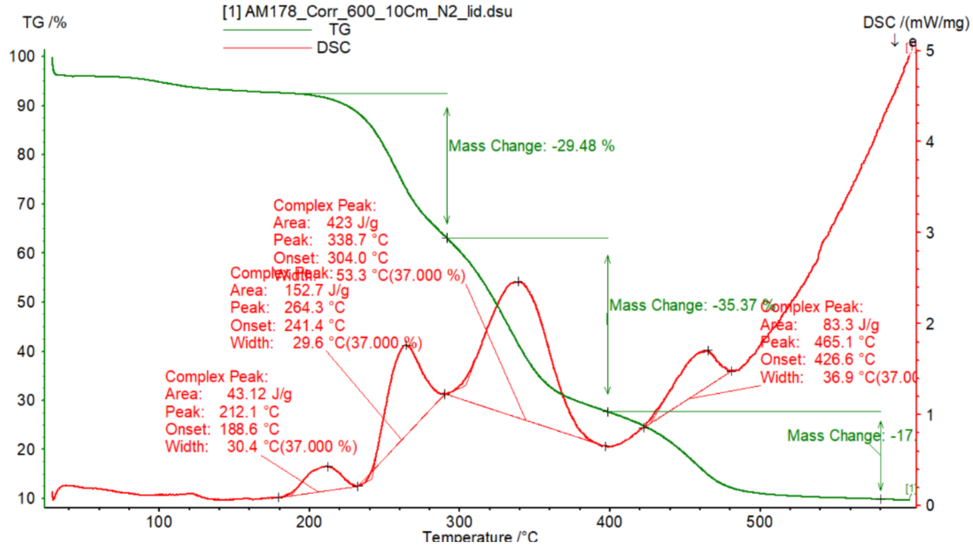


**Figure S71** TGA and DSC results for Entry 4.

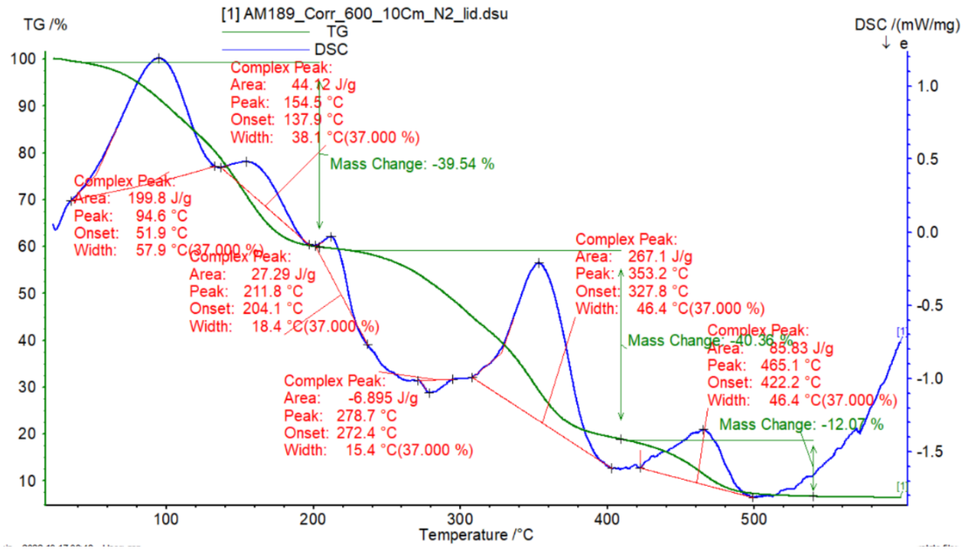


**Figure S72** TGA and DSC results for Entry 5.

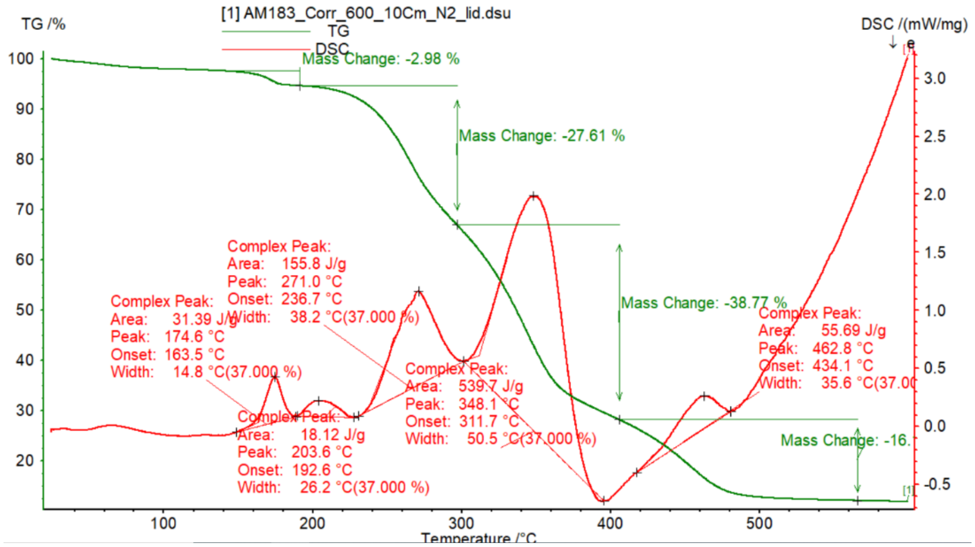


**Figure S73** TGA and DSC results for Entry 6.

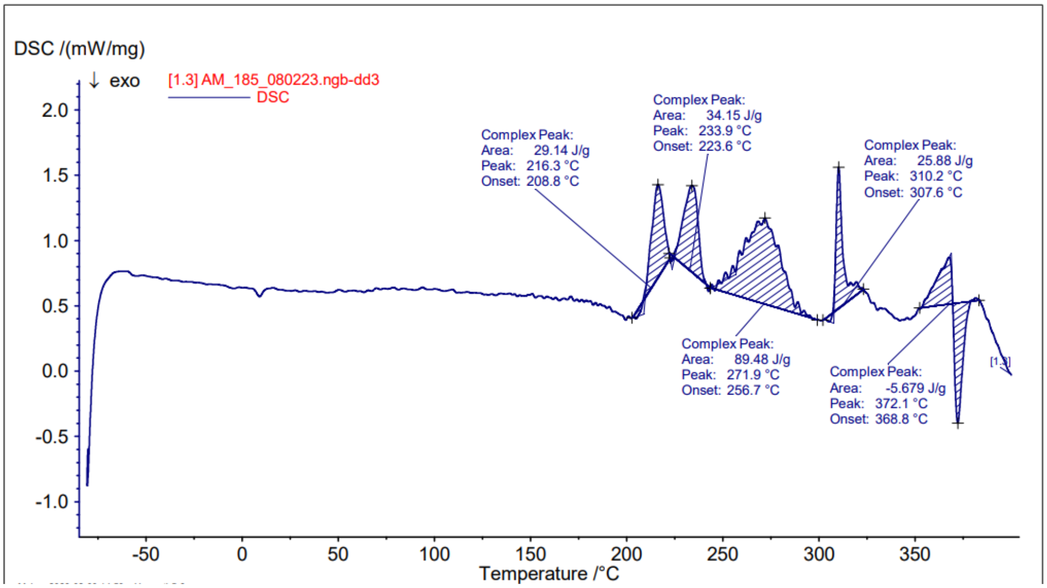


**Figure S74** DSC results for Entry 7.

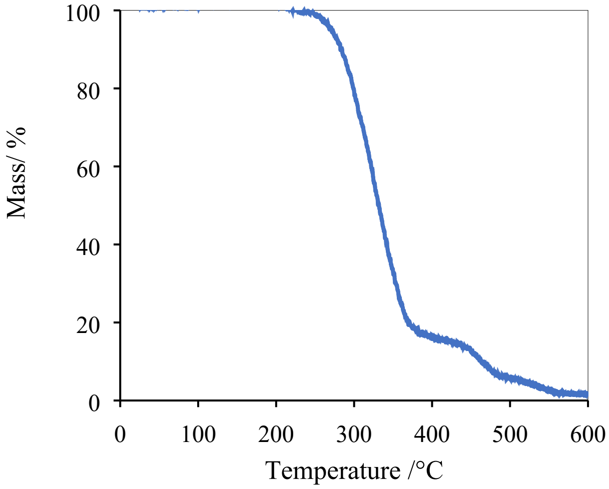


**Figure S75** TGA results for Entry 7.

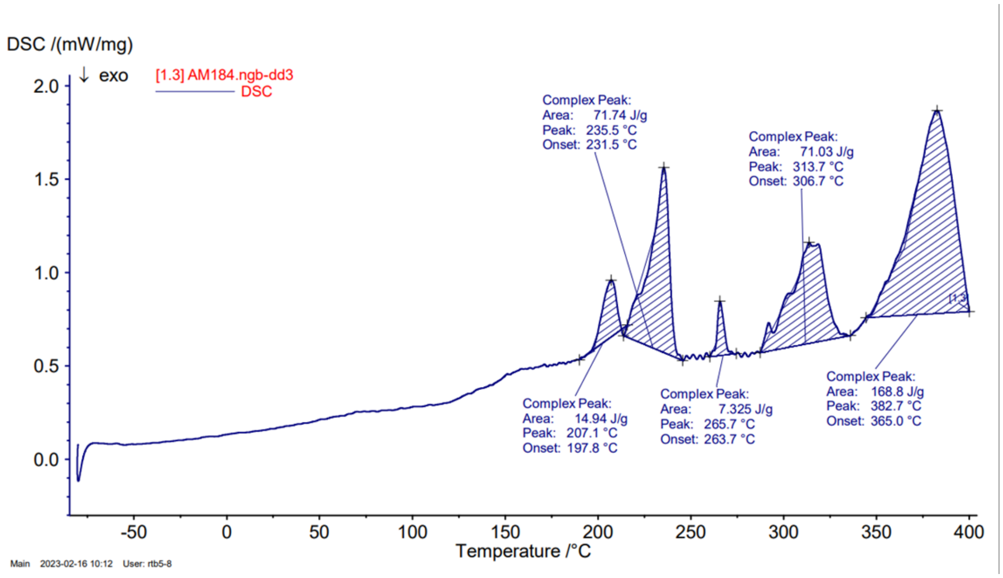


**Figure S76** DSC results for Entry 8.

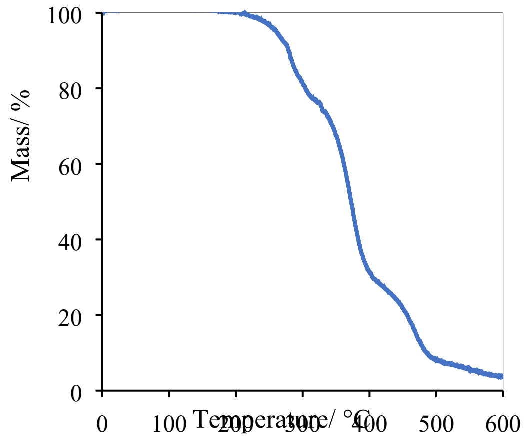


**Figure S77** TGA results for Entry 8.

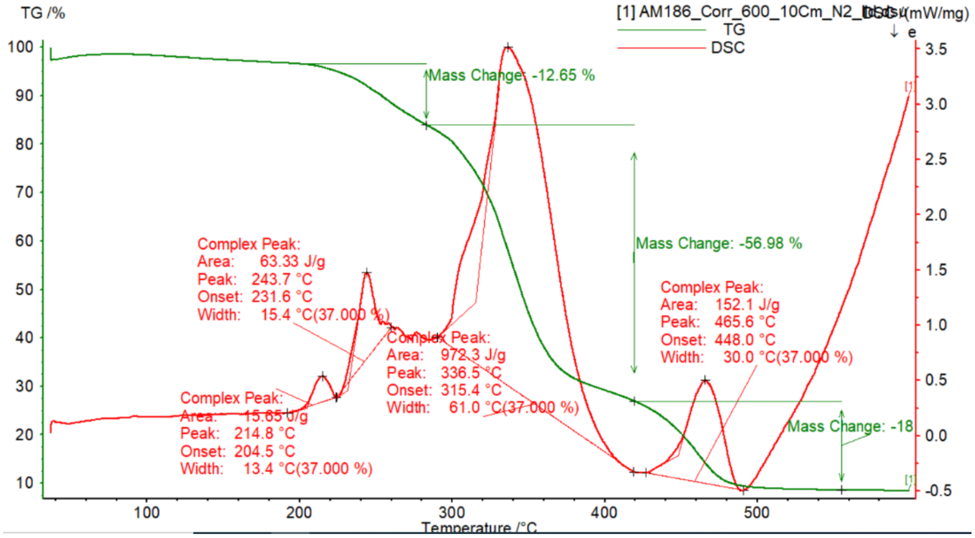


**Figure S78** TGA and DSC results for Entry 9

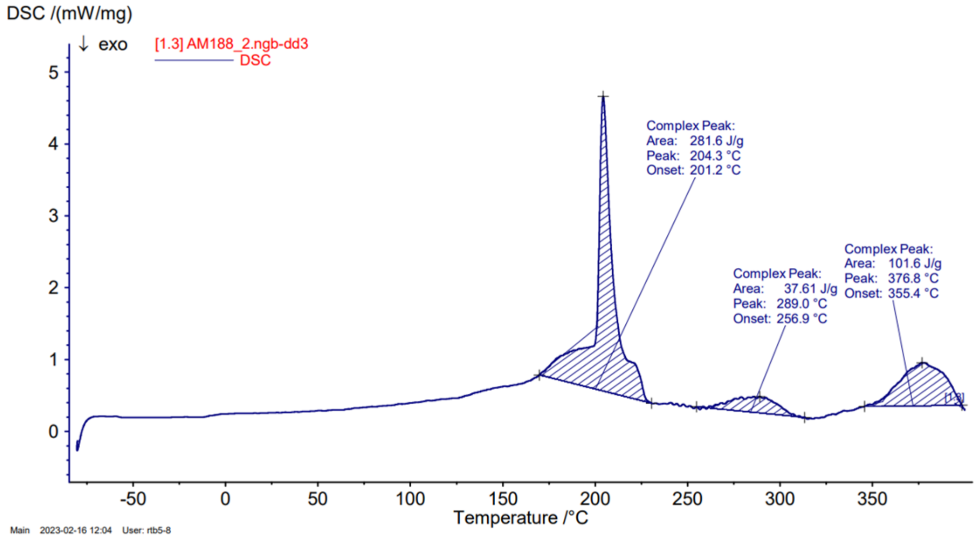


**Figure S79** DSC results for Entry 10.

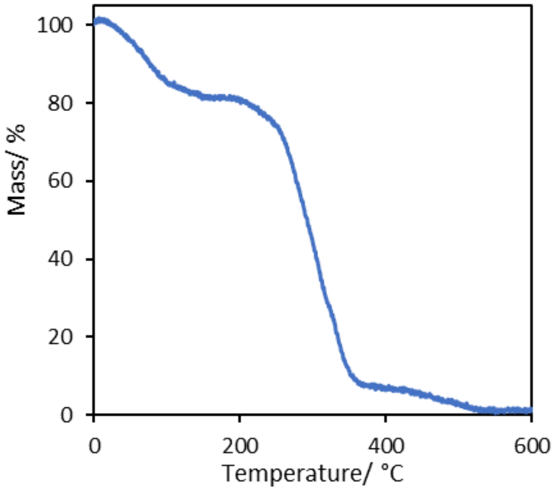


**Figure S80** TGA results for Entry 10.

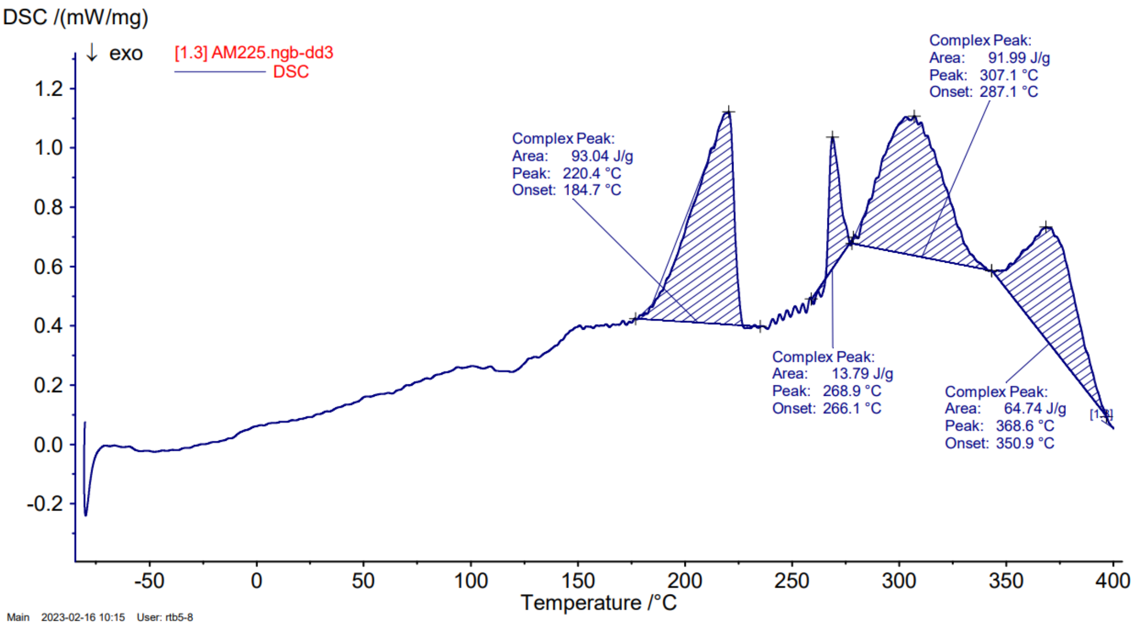


**Figure S81** DSC results for Entry 12.

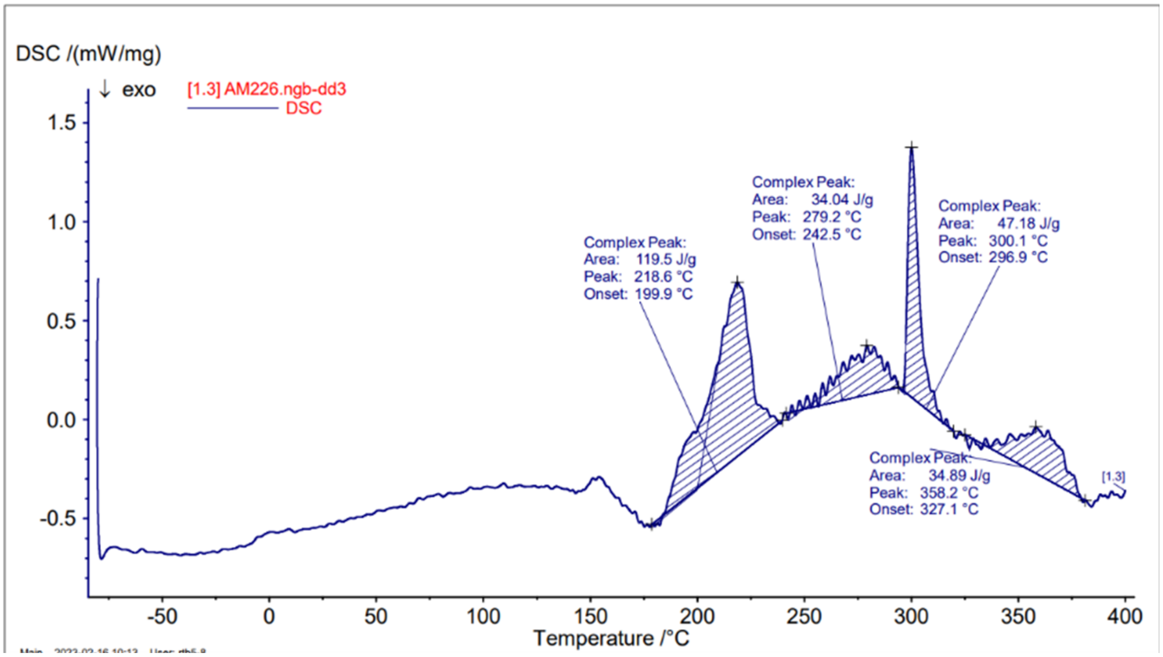


**Figure S82** DSC results for Entry 13.

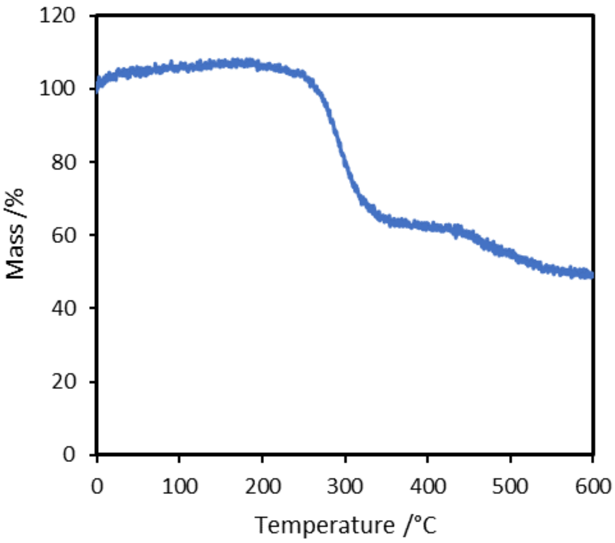


**Figure S83** TGA results for Entry 13.

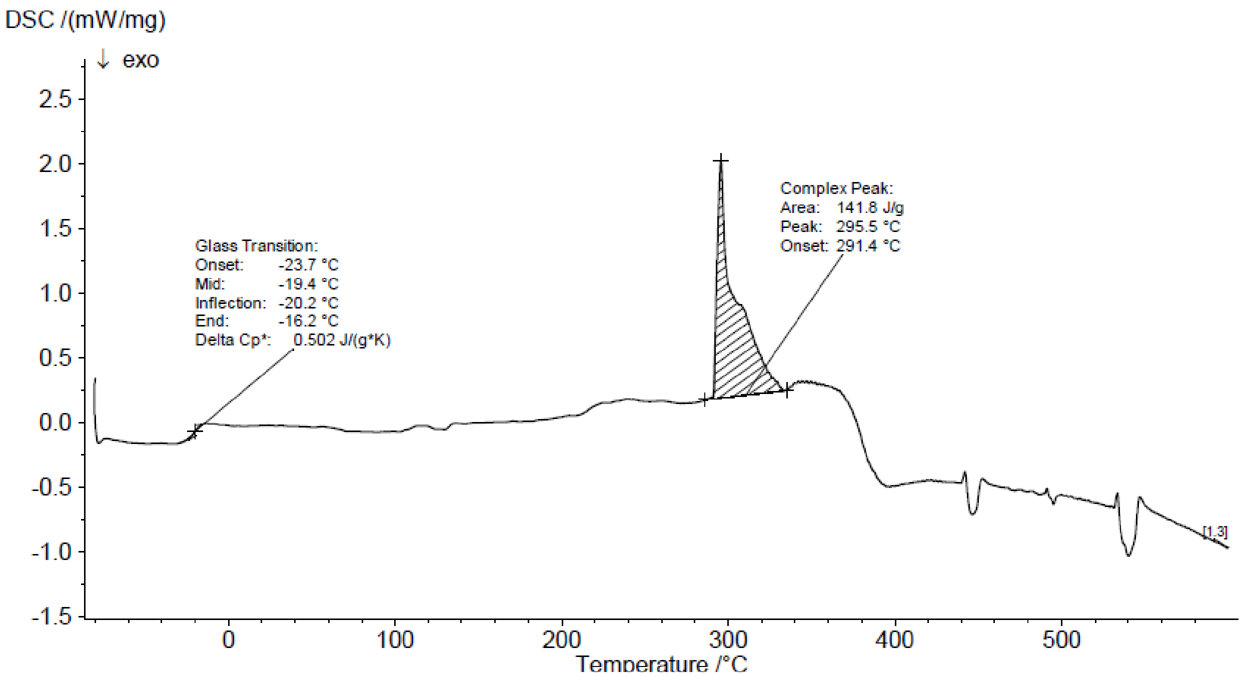


**Figure S84** DSC results for Entry 14. The complex peak observed corresponds to sample decomposition.

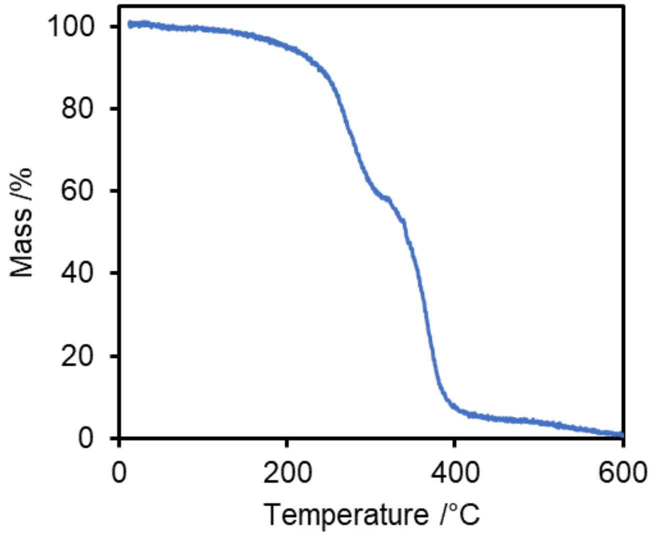


**Figure S85** TGA results for Entry 14.

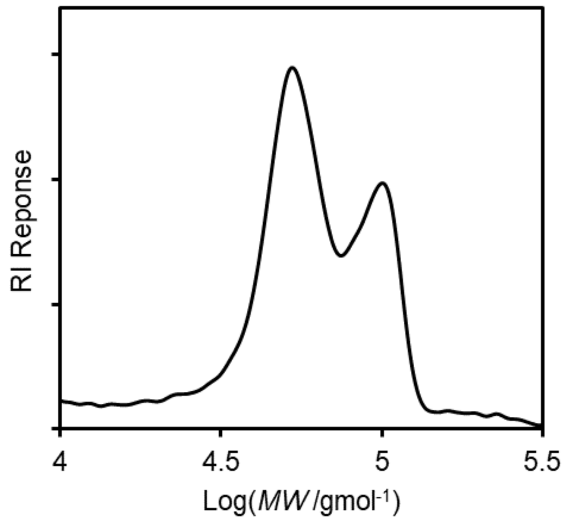


**Figure S86** GPC data obtained for Entry 14.

## 12. Mechanical characterisation of polyureas (Entries 4 and 7, Table S1)

To get reliable results from indentation tests, the contact surface must be flat. Therefore, the specimen surface was thoroughly polished with sandpapers and diamond suspensions.

All the tests were carried out using a KLA iMicro nanoindenter, equipped with a 50mN force actuator. A Berkovich tip was used. Continuous Stiffness Measurements (CSM) were performed, allowing to measure the indentation modulus E* (($E^* = E/1\text{-}\nu^2$), it is used in place of the Young's modulus E when the Poisson's ratio ν is unknown) and H as a function of the indentation load.

The maximum indentation depth was set to 2000 nm.

The tests were carried out using a constant indentation strain rate of 0.1 $s^{-1}$.

The max load was held for 1 second before unloading to quantify creep.

Upon unloading, the load was held again at 10% of max load for 3 minutes to quantify thermal drift and correct the recorded value of load and depth accordingly.

**Table S3** Results for the mechanical characterisation of polyreas.

| Sample | Number of tests | Indentation modulus E* / GPa | Hardness / MPa |
|---|---|---|---|
| 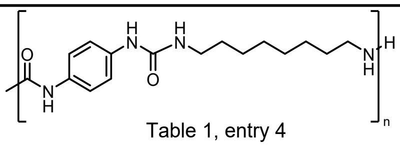 Table 1, entry 4 | 31 | 4.93 ± 0.63 | 291 ± 59 |
| 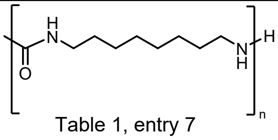 Table 1, entry 7 | 35 | 4.25 ± 0.72 | 252 ± 64 |

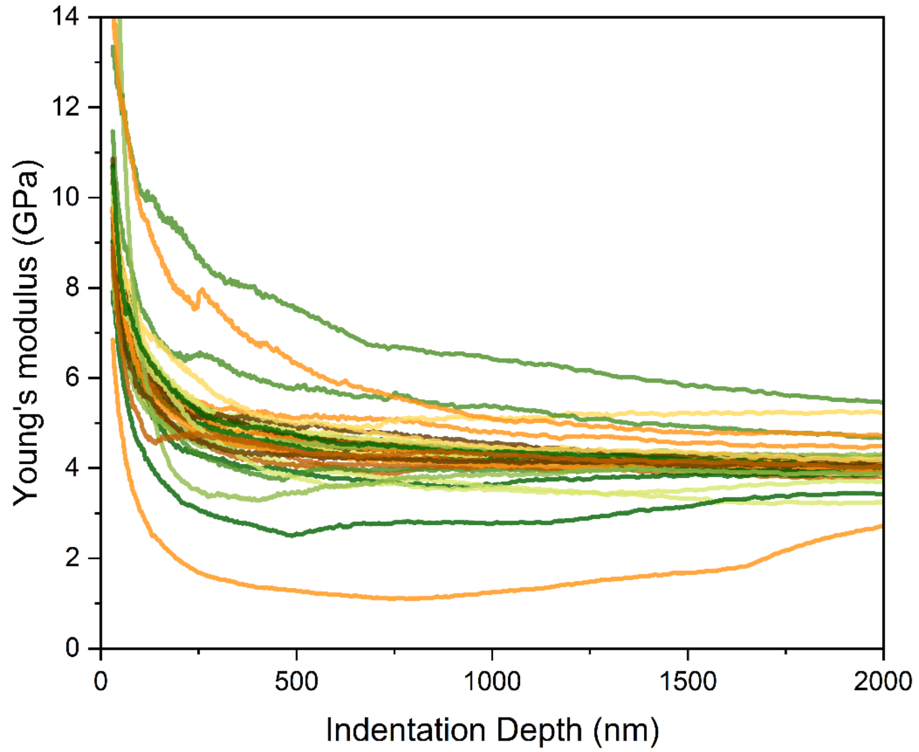

**Figure S87** Young's modulus vs indentation depth plot for the polyurea described in Table S1, entry 4.

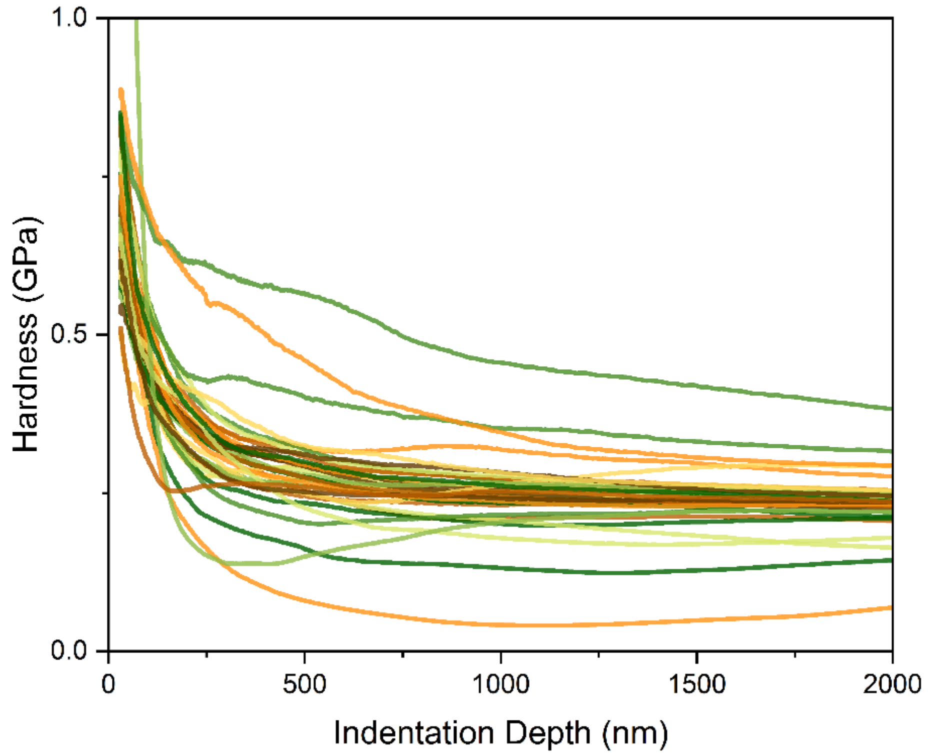

**Figure S88** Hardness vs indentation depth plot for the polyurea described in in Table S1, entry 4.

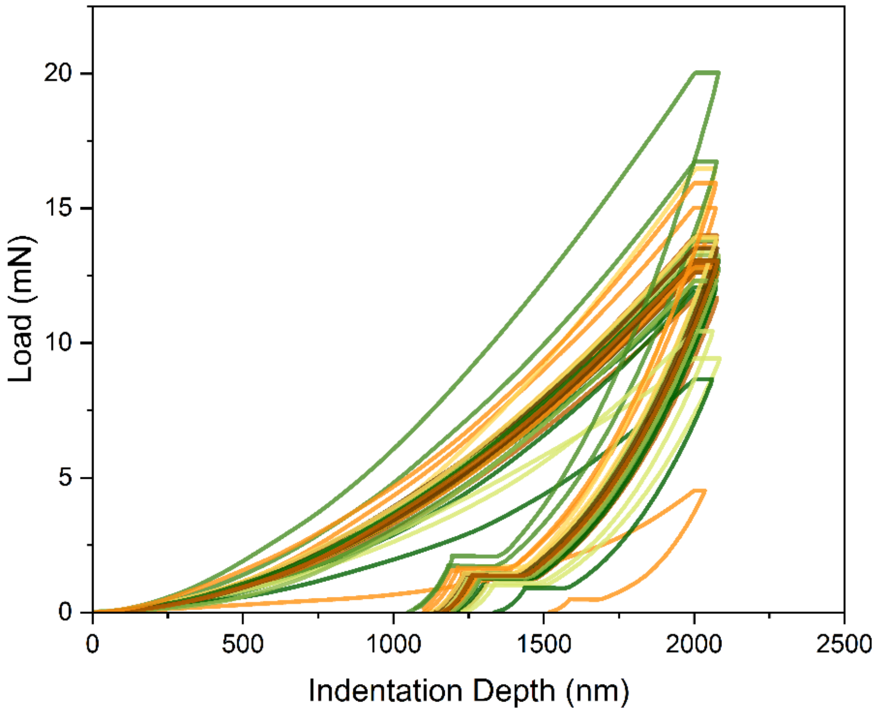


**Figure S89** Load vs indentation depth plot for the polyurea described in in Table S1, entry 4.

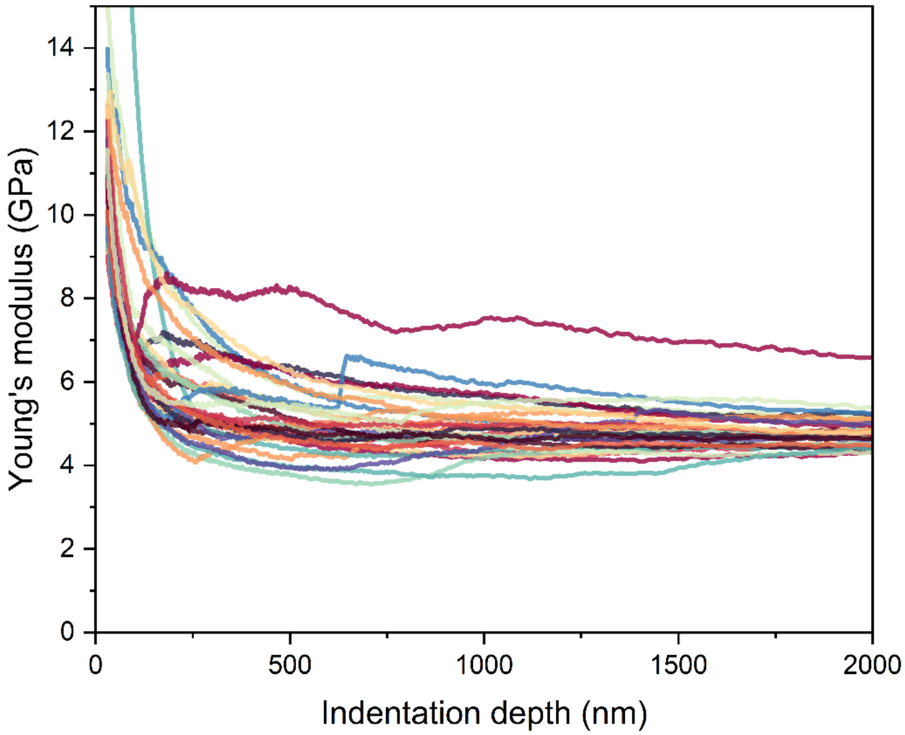


**Figure S90** Young's modulus vs indentation depth plot for the polyurea described in Table S1, entry 7.

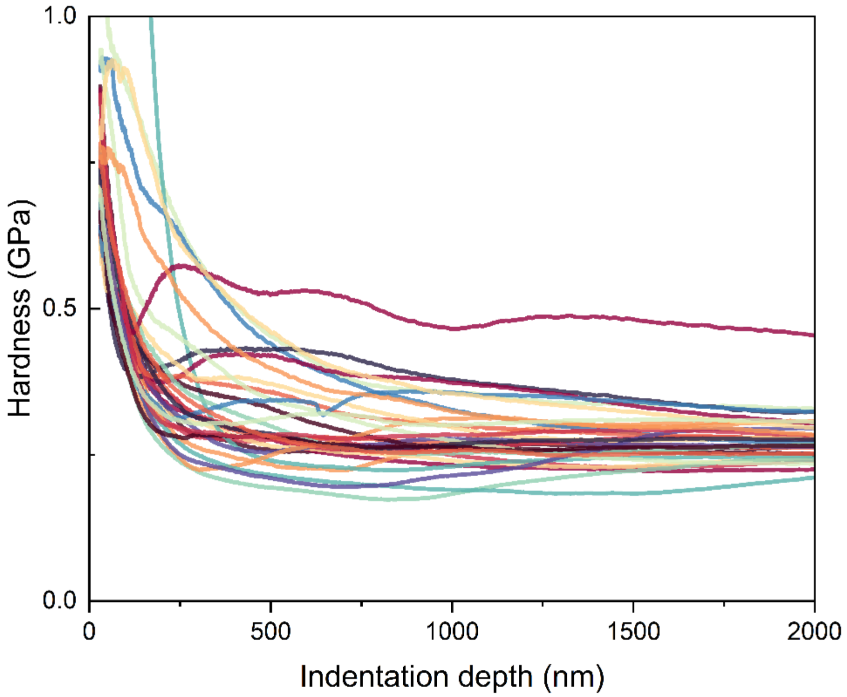


**Figure S91** Hardness vs indentation depth plot for the polyurea described in Table S1, entry 7.

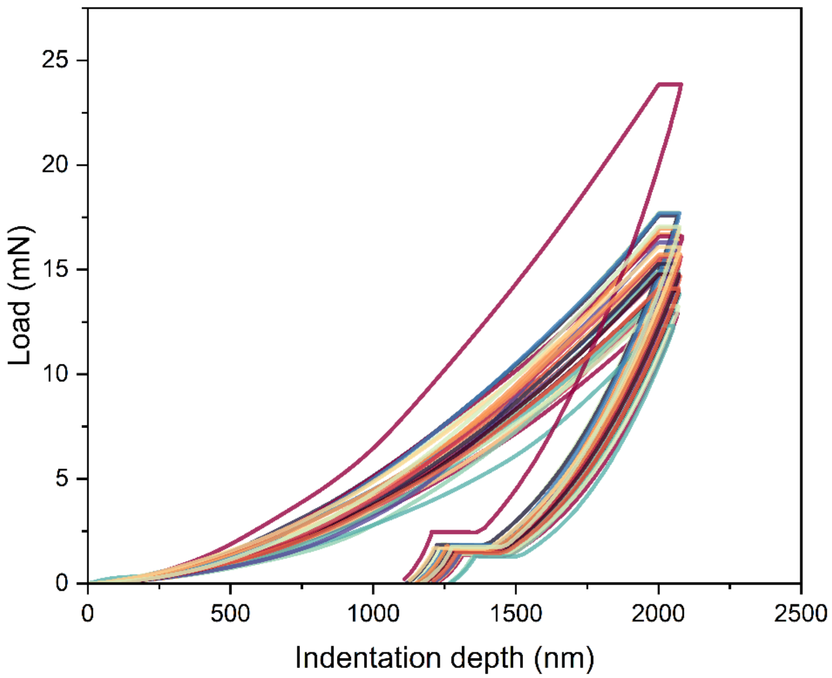


**Figure S92** Load vs indentation depth plot for the polyurea described in Table S1, entry 7.

## 13. SEM and EDX studies.

Figure S83(a-c) shows SEM data from the aliphatic polyurea (made from DF2 and DA1, Entry 7, Table S1) and Figure S83(d-f) shows the SEM data from the aromatic polyurea (made from DF1 and DA1, Entry 4, Table S1). It can be seen from the lower magnification images in Figure S83(a,d) that the samples form larger agglomerations, typically in the region of tens of microns, that are composed of finer features, as shown in the higher magnification images in Figure S83(b,e). These images show that the sample corresponding to the aliphatic polyurea is composed of small sheet-like features typically ~1 – 3 µm long and ~50 – 70 nm thick whereas that corresponding to the aromatic polyurea exhibits a more porous and less densely packed microstructure within the larger agglomerations. Energy dispersive X-ray spectroscopy (EDX) provides a convenient means of rapidly assessing the elemental composition of the samples, confirming the presence of C, N and O; here the Al, Fe and Si signals arising from absorption and fluorescence within the microscope. Slightly increased C and O signal can be seen in aliphatic polyurea compared to that of aromatic polyurea – Figure S83(c) and (f), respectively – though due to the low energies of these X-ray lines, they are particularly prone to absorption/fluorescence hence the quantification would be unreliable.

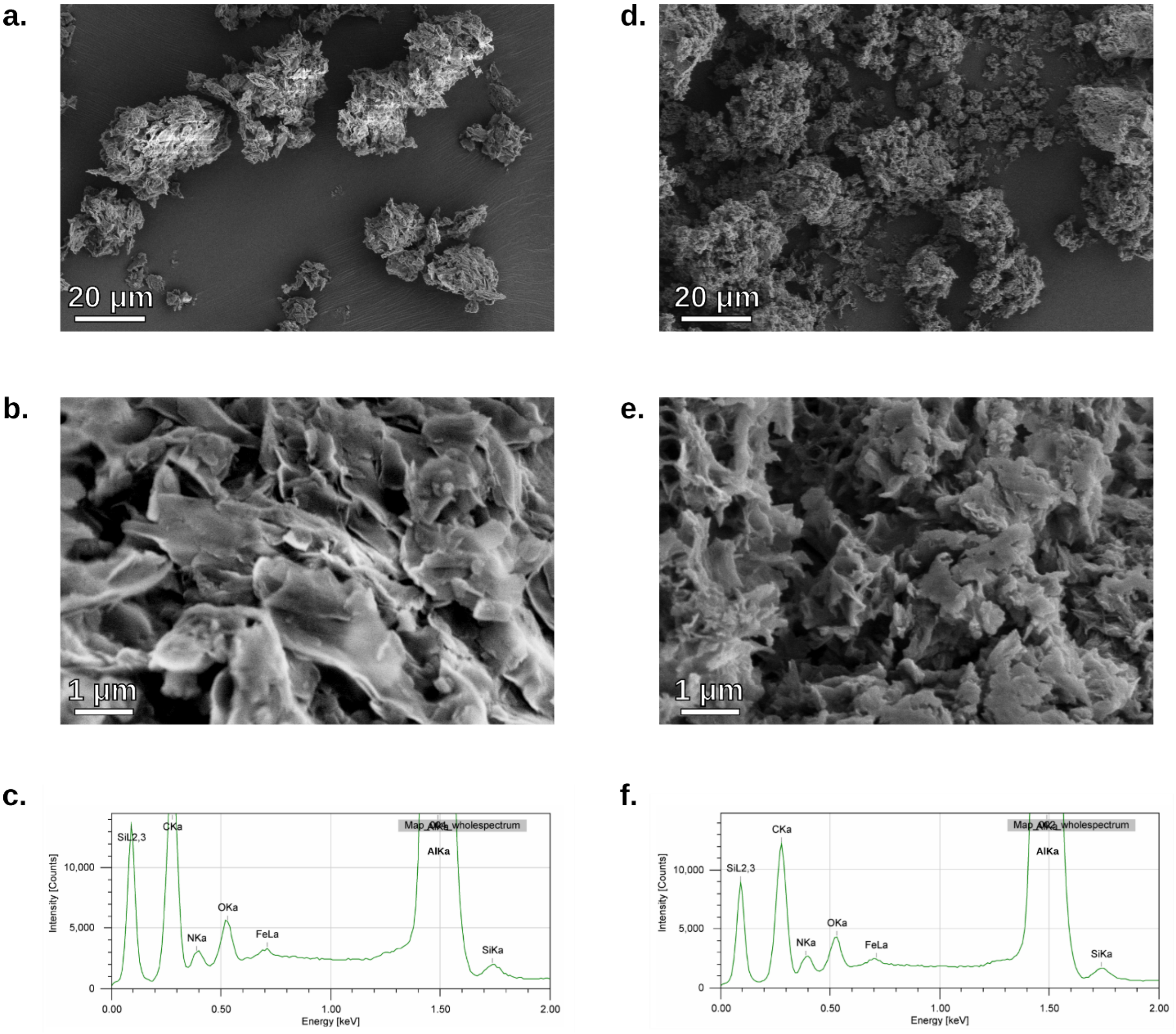


**Figure S93** SEM data from samples corresponding to aliphatic (a-c) and aromatic polyurea (d-f). The top row shows lower magnification secondary electron images, the middle higher magnification ones and the bottom representative EDX spectra, confirming the presence of C, N and O.

## 14. Mechanistic studies

### 14.1. Stochiometric reaction of $Mn(PN^HP\text{-}iPr)(CO)_2Br$ (1) with formamides

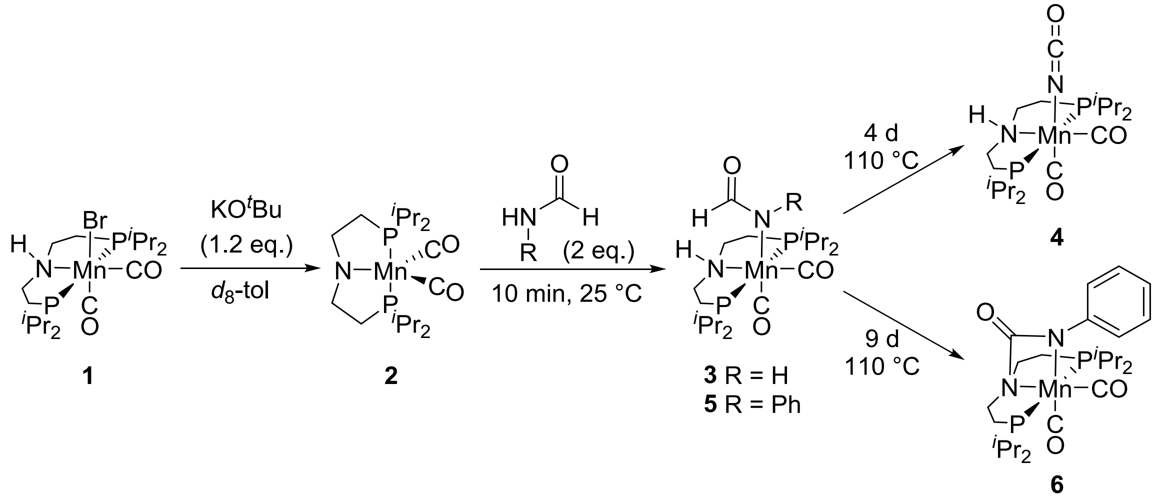


*General procedure:* A J-young's NMR tube is charged with **1** (10 mg, 20 µmol) and KO$^t$Bu (1.2 eq., 2.7 mg, 24 µmol) and $d_8$-toluene (0.5 mL). The sealed NMR tube is heated for 5 minutes at 110 °C to quantitatively generate Mn(PNP-iPr)$(CO)_2$ (**2**) *in situ*. Formamide (*e.g* formamide, 2 eq. 16 µL 40 µmol) is added and the NMR tube shaken vigorously. After 10 minutes at room temperature, the resulting solution is interrogated by NMR spectroscopy and intermediates **3** and **5** identified. The NMR tube is then heated to 110 °C, and the reaction progression monitored by NMR spectroscopy until a single major product (**4** or **6**) is obtained.

***Complex 3*: Mn(PN$^{H}$P-iPr)(NHCOH)(CO)$_2$**

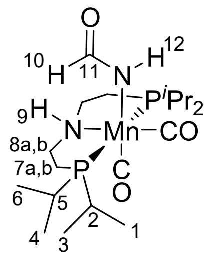


**$^1$H NMR** (500 MHz, *d*$_8$-toluene): $\delta_H$ 9.15 (t, J = 11.3 Hz, 1H, H-9), 8.97 (br s, $\nu_{1/2}$ 60 Hz, H-10/12), 8.26 (br s, $\nu_{1/2}$ 72 Hz, H-10), 2.97-2.79 (m, 2H, H-7a/7b), 2.28-2.20 (m, 2H, H-2), 2.19-2.20 (m, 2H, H-5), 2.07-1.96 (m, 2H, H-7a/7b), 1.83-197 (m, 2H, H-8a/8b), 1.71-1.58 (m, 2H, H-8a/8b) 1.33-1.26 (m, 6H, H-6), 1.27-1.23 (m, 6H, H-3), 1.22-1.16 (m, 6H, H-4), 1.01-0.94 (m, 6H, H-1).

**$^{13}$C{$^1$H} NMR** (126 MHz, *d*$_8$-toluene): $\delta_C$ 176.5 (C-11), 53.0 (t, J = 5.9 Hz, C-8), 28.2 (t, J = 6.1 Hz, C-7), 26.3 (t, J = 8.2 Hz, C-2), 24.7 (t, J = 9.3 Hz, C-5), 20.8 (beneath toluene signal, C-2/C5), 19.2 (C-4), 18.2 (t, J = 3.0 Hz, C-1).

**$^{31}$P{$^1$H} NMR** (162 MHz, *d*$_8$-toluene): $\delta_P$ 83.9 (s).

**HR-MS** (ESI, MeCN, Da): *m/z* 460.1799 ([M]$^+$, $C_{19}H_{39}MnP_2N_2O_3$, calc. 460.1816), with the correct isotope pattern.

***Complex 4*: Mn(PN$^{H}$P-iPr)(NCO)(CO)$_2$**

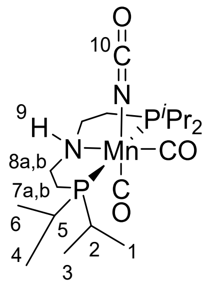


Dissolved $H_2$ is observed in the $^{1}H$ NMR spectrum of the synthesis of complex **4** by the presence of a signal with $\delta_H$ 4.51 ppm.

**$^{1}$H NMR** (500 MHz, $d_8$-toluene): $\delta_H$ 3.87 (m, 1H, H-9), 2.88-2.82 (m, 2H, H-2), 2.82-2.75 (m, 2H, H-7a), 2.28-2.15 (m, 2H, H-5), 1.85-1.75 (m, 4H, H-7b/8a), 1.55-1.49 (m, 6H, H-3), 1.44-1.37 (m, 2H, H-8b), 1.32-1.29 (m, 6H, H-6), 1.22-1.18 (m, 6H, H-4), 1.14-1.10 (m, 6H, H-1).

**$^{13}$C{$^{1}$H} NMR** (126 MHz, $d_8$-toluene): $\delta_C$ 159.7 (C-10), 53.2 (t, J = 5.2 Hz, C-8), 29.9 (t, J = 10.4 Hz, C-7), 28.4 (t, J = 6.8 Hz, C-2), 24.7 (t, J = 9.3 Hz, C-5), 19.7 (C-6), 19.6 (C-3), 18.8 (C-4), 18.4 (C-1).

**$^{31}$P{$^{1}$H} NMR** (162 MHz, $d_8$-toluene): $\delta_P$ 90.7 (s).

**HR-MS** (ESI, MeCN, Da): *m/z* 457.1929 ([M-H]$^+$, $C_{19}H_{39}MnP_2N_2O_3$, calc. 457.1587), with the correct isotope pattern.

***Complex 5*: *in solution*: [Mn(PN^HP-iPr)(NPhCOH)(CO)$_2$]**

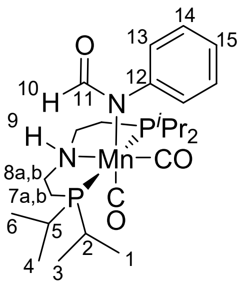


**$^1$H NMR** (500 MHz, $d_8$-toluene): $\delta_H$ 8.57 (s, 1H, H-10), 8.21 (br t, 1H, H-9), 7.18 (t, J = 7.5 Hz, 2H, H-13), 6.92-6.88 (overlapping m, 3H, H-14 and H-15), 2.96-2.85 (m, 2H, H-7a), 2.22-2.12 (overlapping m, 6H, H-7b and H-2/H-5), 1.87-1.73 (m, 2H, H-8a), 1.67-1.59 (m, 2H, H-8b), 1.39 (*apparent* q, J = 7.3 Hz, 6H, H-1/H-3), 1.32 (*apparent* q, J = 7.1 Hz, 6H, H-4/6), 1.21 (*apparent* q, J = 7.3 Hz, H-4/6), 1.04-0.95 (m, 6H, H-1/H-3).

The following signals were observed corresponding to free/H-bonded formanilide in the solution: $\delta_H$ 7.98 (br s), 7.57 (d, J = 8.2 Hz), 7.05 (t, J = 7.7 Hz), 6.83 (t, J = 7.5 Hz), 6.67 (br H, $\nu_{1/2}$ ~300 Hz).

**$^{13}$C{$^1$H} NMR** (126 MHz, $d_8$-toluene): $\delta_C$ 171.2 (C-11), 138.8 (C-12), 129.2 (C-13), 121.8 (C-15), 121.5 (C-14), 52.8 (t, J = 5.9 Hz, C-8), 27.7 (t, J = 5.9 Hz, C-7), 25.9 (t, $^3J_{CP}$ 7.5 Hz, C-2), 24.5 (t, J = 9.8 Hz, C-5), 18.8 (C-4,6), 17.9 (C-1,3).

$^{13}$C{$^1$H} signals corresponding to C-3 and C-6 are obscured beneath residual *protio*-toluene signal (as confirmed by a $^1$H-$^{13}$C HSQC NMR spectrum). The following signals were observed corresponding to free/H-bonded formanilide in the solution: $\delta_C$ 151.6, 129.2, 125.4, 123.8, 119.6.

**$^{31}$P{$^1$H} NMR** (162 MHz, $d_8$-toluene): $\delta_P$ 87.0 (s).

**HR-MS** (ESI, MeCN, Da): *m/z* 502.2052 ([M–(CO)$_2$–H+Na]$^+$, $C_{23}H_{42}CMnN_2P_2ONa$, calc. 502.2045); 416.1664 ([M–(PhNCOH)]$^+$, $C_{18}$H37MnN$P_2O_2$, calc. 416.1680) with the correct isotope patterns.

**GC-MS** analysis of the reaction mixture diluted in DCM post heating allows for interrogation of volatile organic components, these data are summarized in Table S4

**Table S4.** Summary of GC-MS data obtained from reaction mixture.

| Retention Time /min | Observed *m/z* /Da | Ion fragment | Compound | Theoretical *m/z* /Da |
|---|---|---|---|---|
| 14.31 | 121.05 | M$^+$ | formanilide | 121.05 |

***Complex 6*: Mn(PN(CONPh)P-iPr)(CO)$_2$**

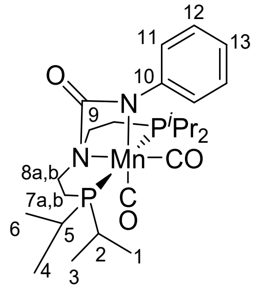


Dissolved $H_2$ is observed in the $^1$H NMR spectrum of the synthesis of complex **4** by the presence of a signal with $\delta_H$ 4.51 ppm.

**$^1$H NMR** (500 MHz, $d_8$-toluene): $\delta_H$ 7.21 (t, J = 7.8 Hz, H-17), 7.04-7.00 (m, overlapping signal with residual *protio*-toluene, 1H, H-12), 6.66 (t, J = 7.4 Hz, 1H, H-13), 6.31 (d, J = 7.4 Hz, 2H, H-11), 3.36-3.25 (m, 2H, H-7b), 2.22-2.12 (m, 2H, H-7a), 2.07-2.00 (m, 2H, H-5), 1.65-1.55 (m, 2H, H-2), 1.55-1.47 (m, 2H, H-8a), 1.47-1.37 (m, 2H, H-8b), 1.33 (dd, J = 14.9, 6.9 Hz, 6H, H-6), 1.31-1.26 (m, 6H, H-3), 1.15 (*apparent* q, J = 7.2 Hz, H-4), 0.64-0.60 (m, 6H, H-1).

Following signals were obtained corresponding to free or H-bonded aniline: $\delta_H$ 8.15 (br s, $\nu_{1/2}$ 560 Hz), 6.86 (*apparent* t, J = 7.3 Hz).

**$^{13}$C{$^1$H} NMR** (126 MHz, $d_8$-toluene): $\delta_C$ 156.8 (C-9), 129.3 (C-12), 124.2 (C-10), 118.2 (C-13), 115.0 (C-11), 55.6 (t, J = 4.1 Hz, C-8), 28.3 (t, J = 7.9 Hz, C-7), 24.6 (t, J = 5.4 Hz, C-8), 23.9 (t, J = 9.3 Hz, C-5),18.7 (C-4), 17.2 (t, J = 3.4 Hz, C-1).

$^{13}$C{$^1$H} NMR signals corresponding to C-3 and C-6 are obscured by residual *protio*-toluene signal (as confirmed by a $^1$H-$^{13}$C HSQC NMR spectrum). Following signals were obtained corresponding to free or H-bonded aniline: $\delta_C$ 147.4, 147.2, 129.2, 128.2, 122.5.

**$^{31}$P{$^1$H} NMR** (162 MHz, $d_8$-toluene): $\delta_P$ 89.2 (s).

**HRMS** (ESI, MeCN, Da): *m/z* 534.1954 ([M]$^+$, $C_{25}H_{41}MnP_2N_2O_3$, calc. 534.1973) with the correct isotope pattern.

**GC-MS** analysis of the reaction mixture diluted in DCM post heating allows for interrogation of volatile organic components, these data are summarised in Table S5.

**Table S5** Summary of GC-MS data obtained from reaction mixture.

| Retention Time /min | Observed *m/z* /Da | Ion fragment | Compound | Theoretical *m/z* /Da |
|---|---|---|---|---|
| 8.18 | 119.04 | M$^+$ | phenylisocyanate | 119.04 |
| 8.57 | 93.07 | M$^+$ | aniline | 93.06 |
| 10.37 | 106.08 | M-H[a] | *N*-methylaniline | 106.07 |
| 14.31 | 121.05 | M$^+$ | formanilide | 121.05 |

[a] M$^+$ ion is also observed with lower TIC.

## 14.2 NMR spectra of complex 3

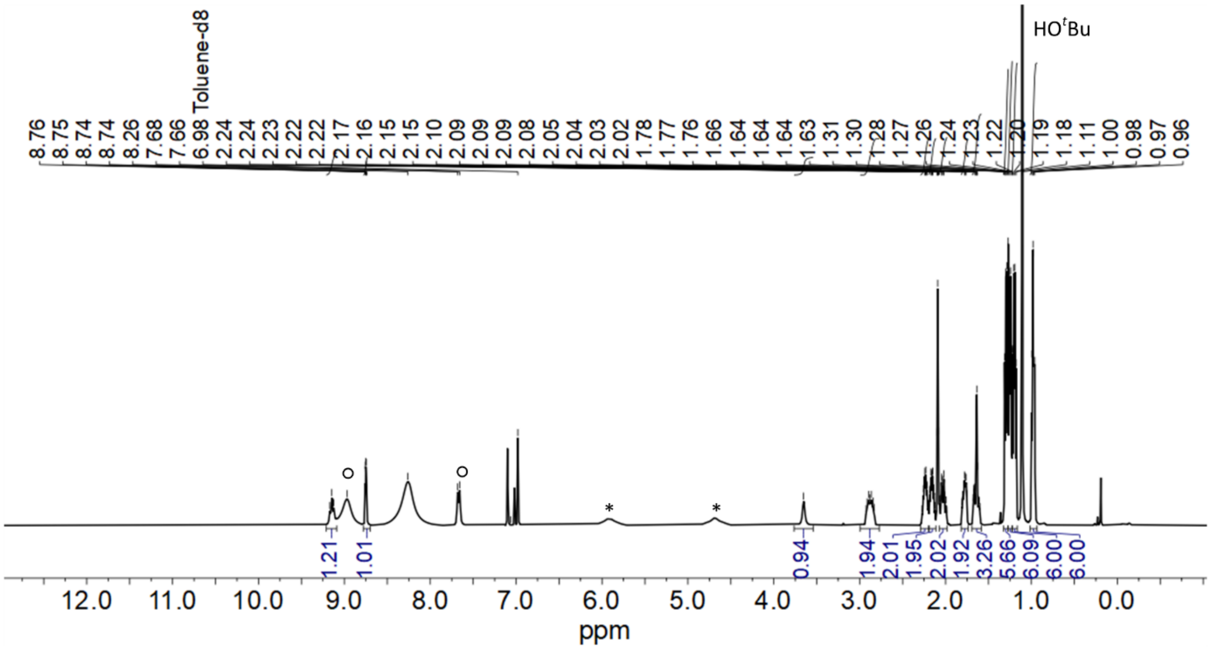


**Figure S94** $^{1}H$ NMR ($d_8$-toluene, 500 MHz) spectrum of complex **3**. ° denotes residual formamide. *denotes an unknown impurity in commercial formamide.

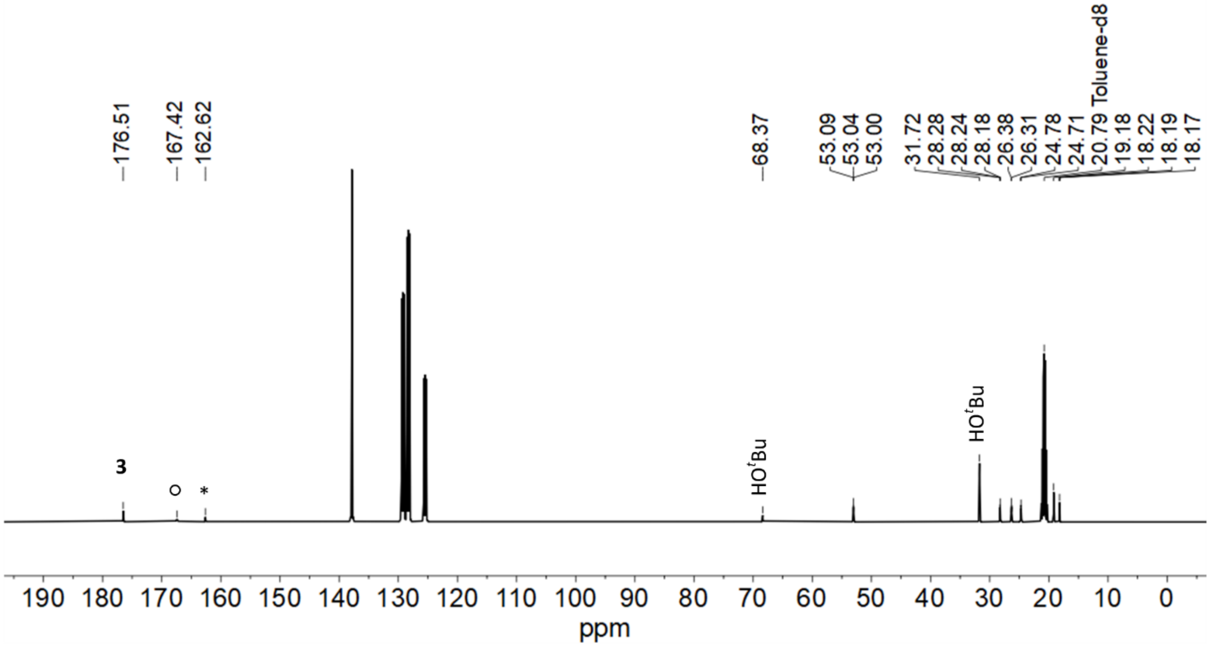


**Figure S95** $^{13}C\{^{1}H\}$ NMR ($d_8$-toluene, 126 MHz) spectrum of complex **3**. ° denotes residual formamide, * denotes an unknown impurity which is present within commercial formamide.

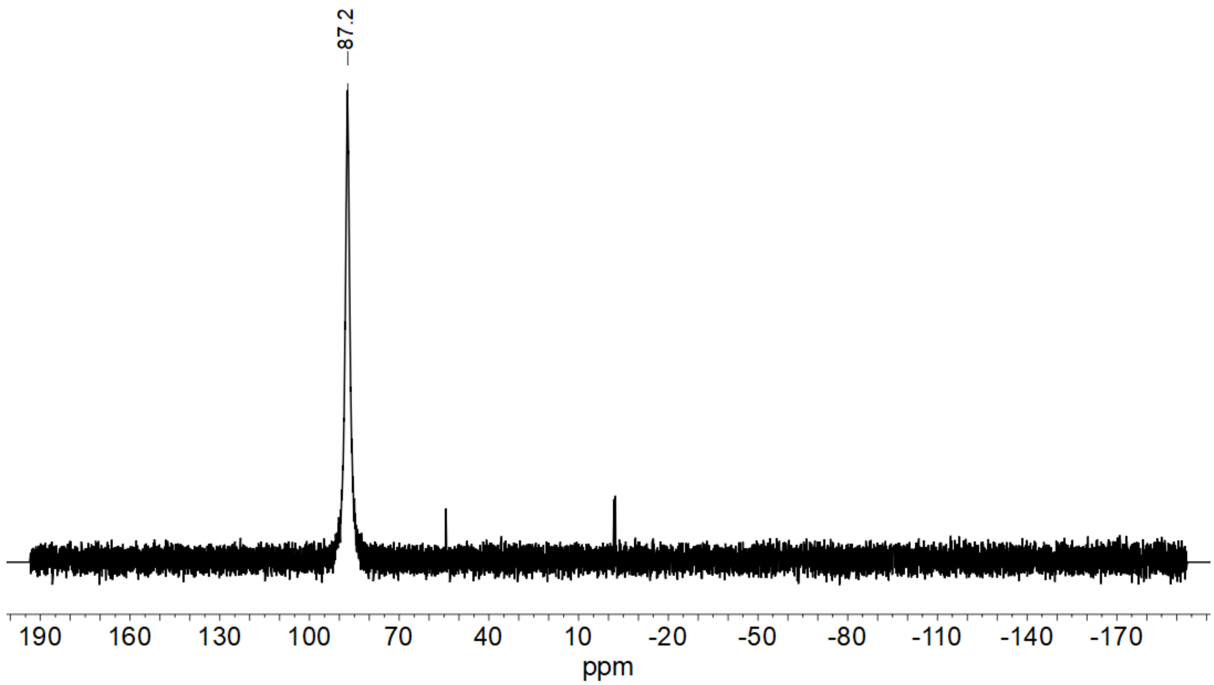


**Figure S96** $^{31}P\{^{1}H\}$ NMR (162 MHz, $d_8$-toluene) spectrum of complex **3**.

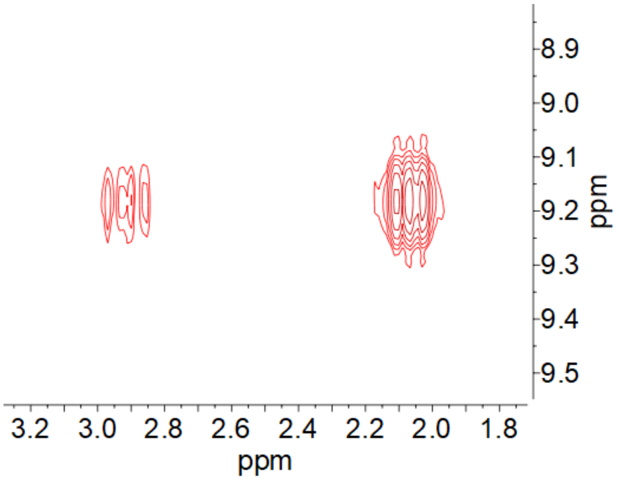


**Figure S97** $^{1}H$-$^{1}H$ COSY NMR ($d_8$-toluene, 500 MHz) partial spectrum for complex **3** showing correlation between N-H and backbone C-H protons.

## 14.3 NMR spectra of complex 4

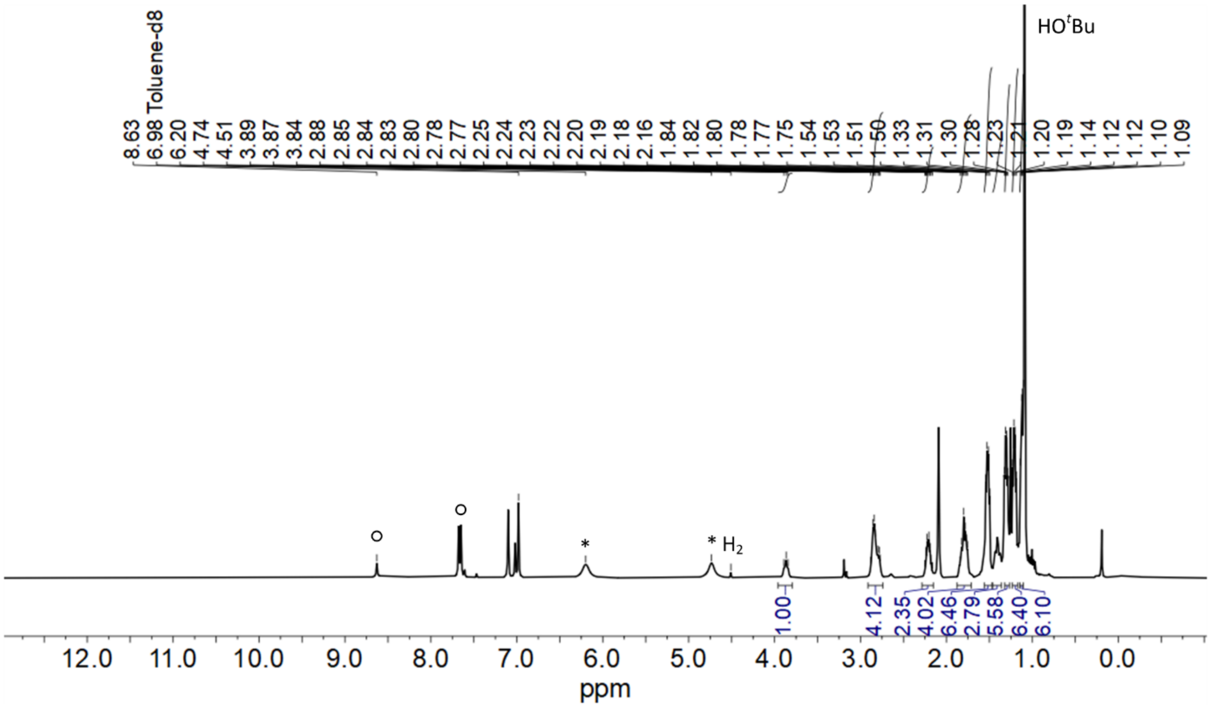


**Figure S98** $^1$H NMR ($d_8$-toluene, 500 MHz) spectrum of complex **4**. ° denotes residual formamide. *denotes an unknown impurity present in commercial formamide.

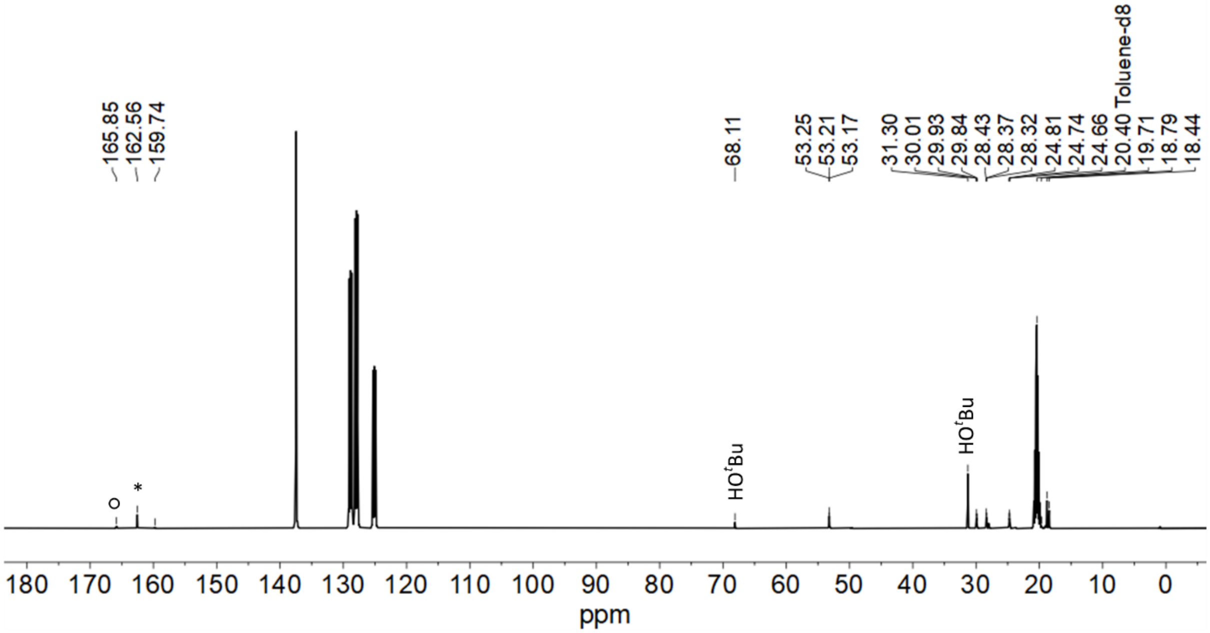


**Figure S99** $^{13}$C{$^1$H} NMR ($d_8$-toluene, 126 MHz) spectrum of complex **4**. ° denotes residual formamide, * denotes an unknown impurity which is present within commercial formamide.

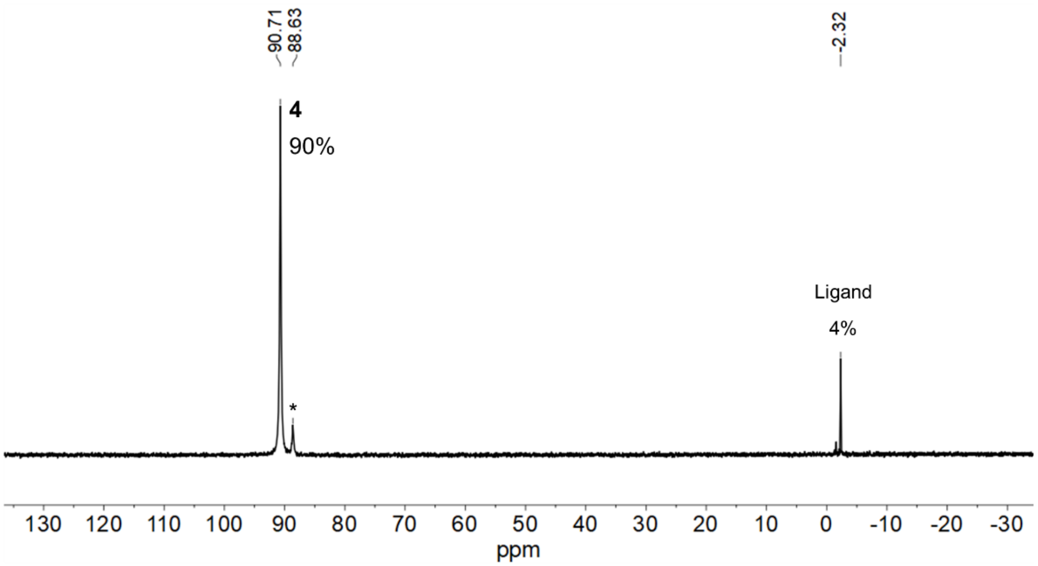


**Figure S100** $^{31}P\{^{1}H\}$ NMR (162 MHz, $d_8$-toluene) spectra of reaction progression for the generation of complex **4**.

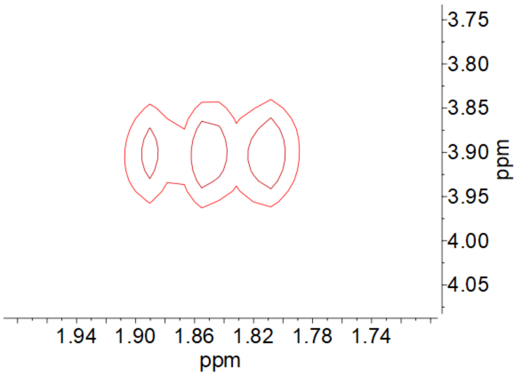


**Figure S101** $^{1}H$-$^{1}H$ COSY NMR ($d_8$-toluene, 500 MHz) partial spectrum for complex **4** showing correlation between N-H and backbone C-H protons.

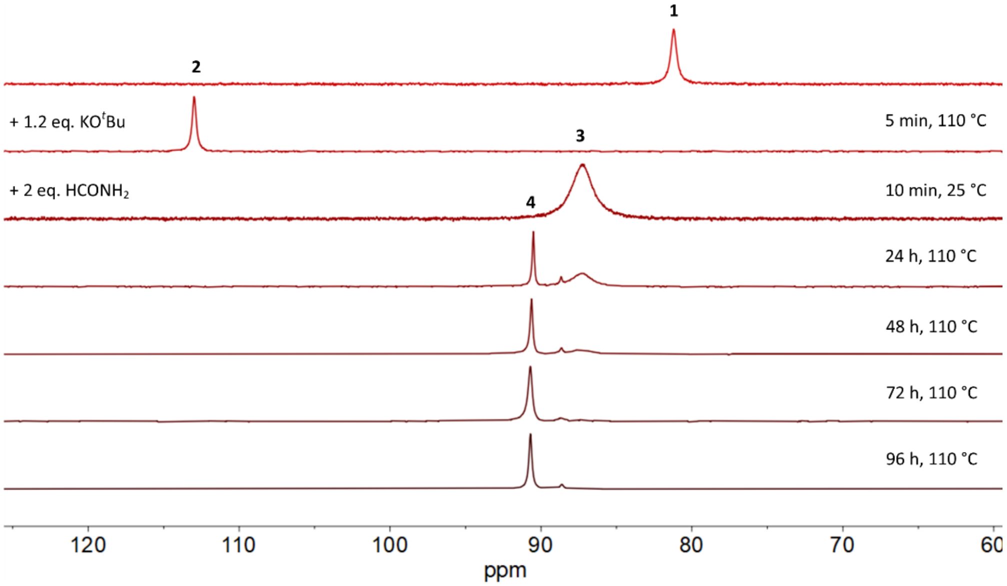


**Figure S102** $^{31}P\{^1H\}$ NMR (162 MHz, $d_8$-toluene) spectra of reaction progression for the generation of complex **4**.

## 14.4 NMR spectra of complex 5

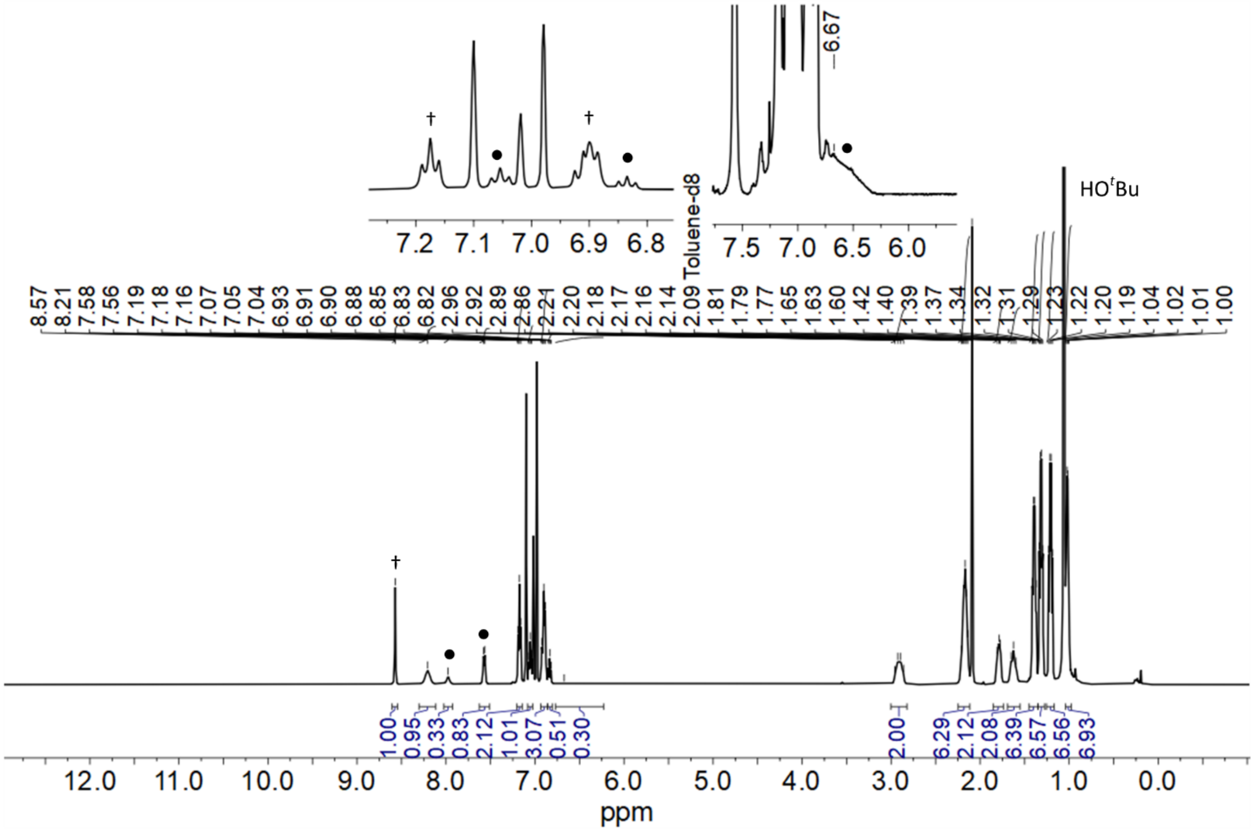


**Figure S103** $^{1}$H NMR ($d_8$-toluene, 500 MHz) spectrum of complex **5**. $^{\dagger}$ denotes N-H activated formanilide protons. $^{\bullet}$ denotes H-bonded formanilide protons.

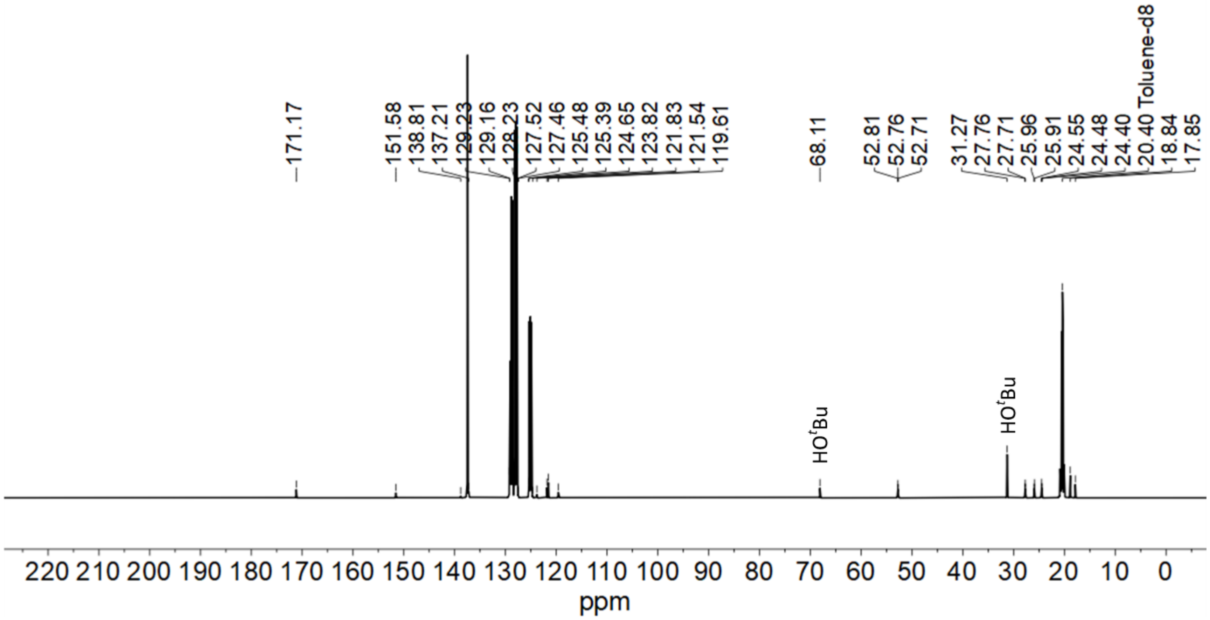


**Figure S104** $^{13}$C{$^{1}$H} NMR ($d_8$-toluene, 126 MHz) spectrum of complex **5.**

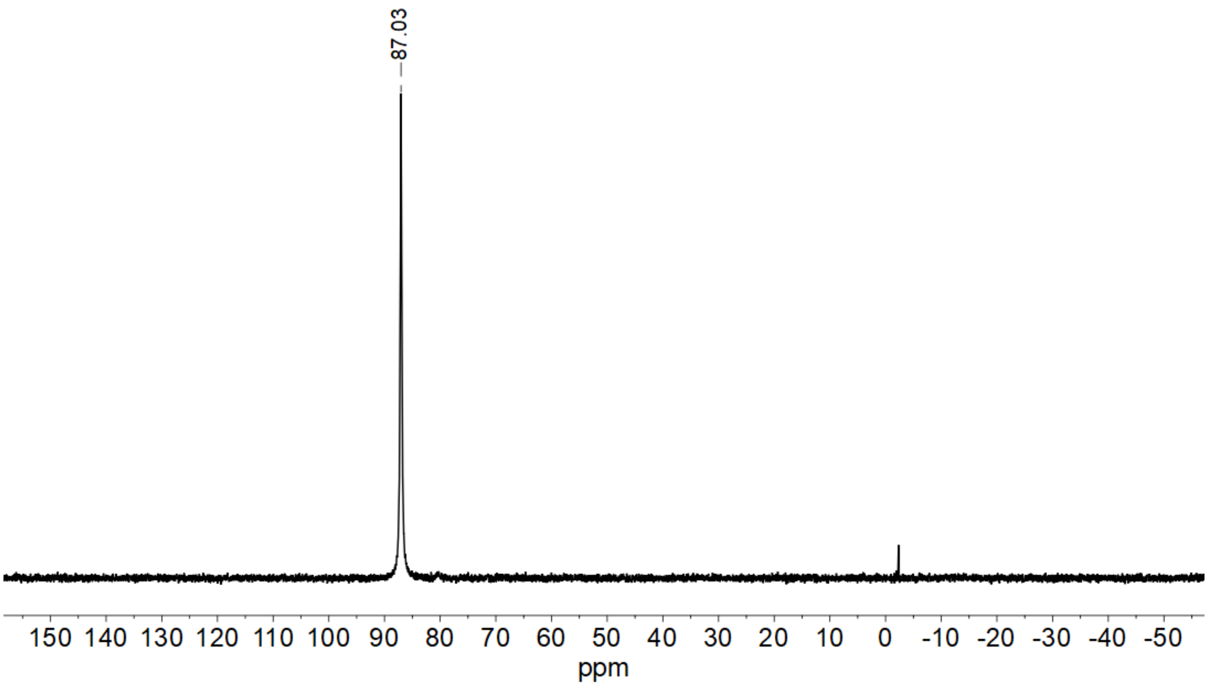


**Figure S105** $^{31}P\{^1H\}$ NMR (162 MHz, $d_8$-toluene) spectrum of complex **5**.

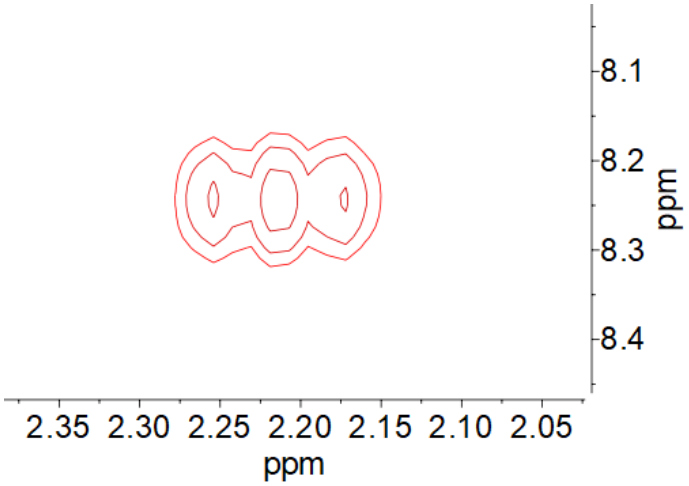


**Figure S106** $^1H$-$^1H$ COSY NMR ($d_8$-toluene, 500 MHz) partial spectrum for complex **5** showing correlation between N-H and backbone C-H protons.

## 14.5 NMR spectra of complex 6

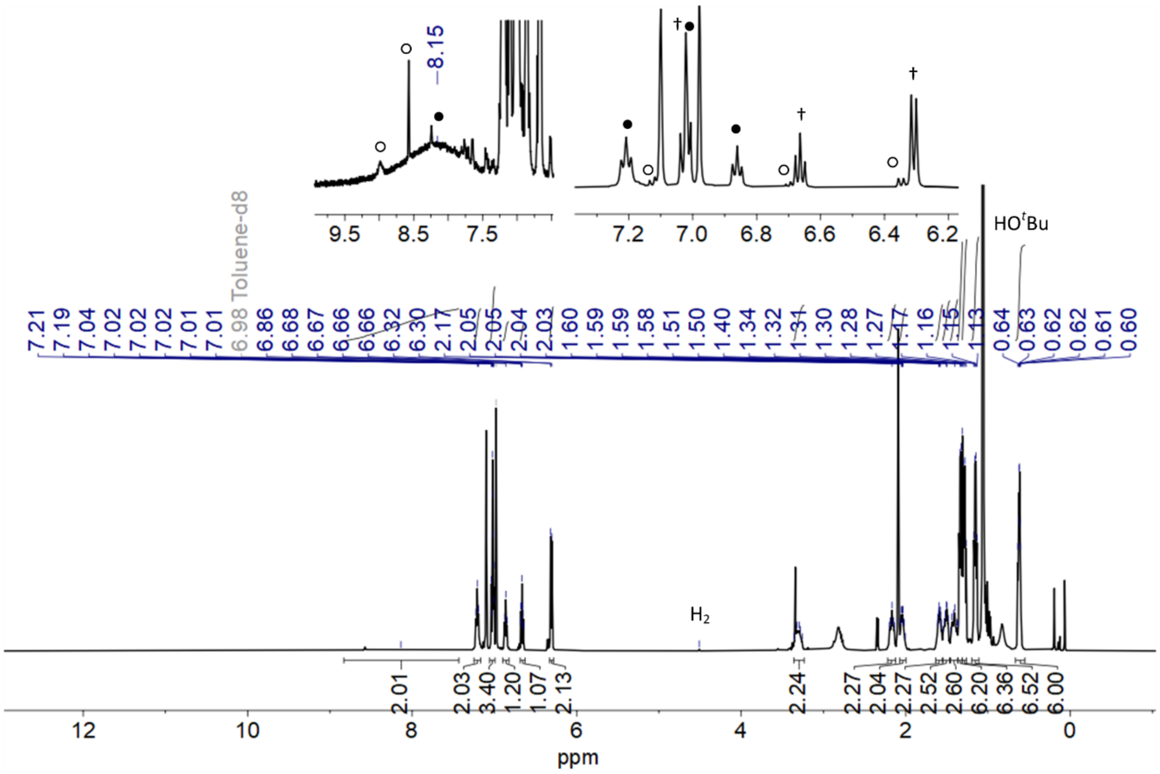


**Figure S107** $^1$H NMR ($d_8$-toluene, 500 MHz) spectrum of complex 6. $^†$denotes Ph protons of N-H/C-H activated formanilide. • denotes H-bonded formanilide. ° denotes free formanilide.

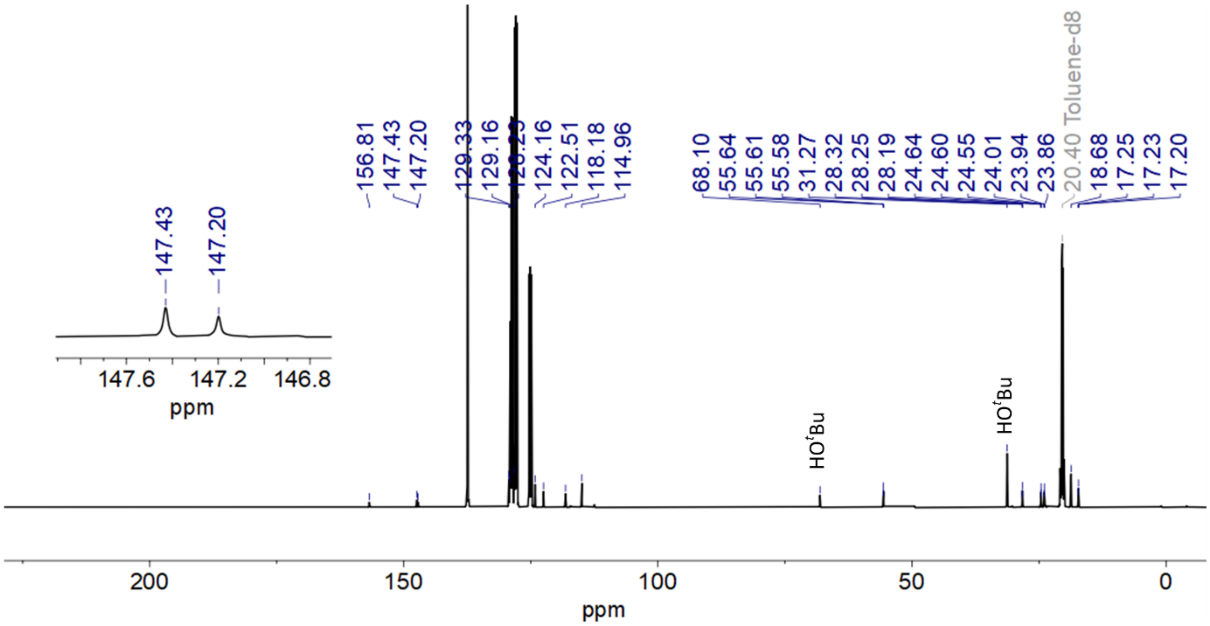


**Figure S108** $^{13}C\{^1H\}$ NMR ($d_8$-toluene, 126 MHz) spectrum of complex 6.

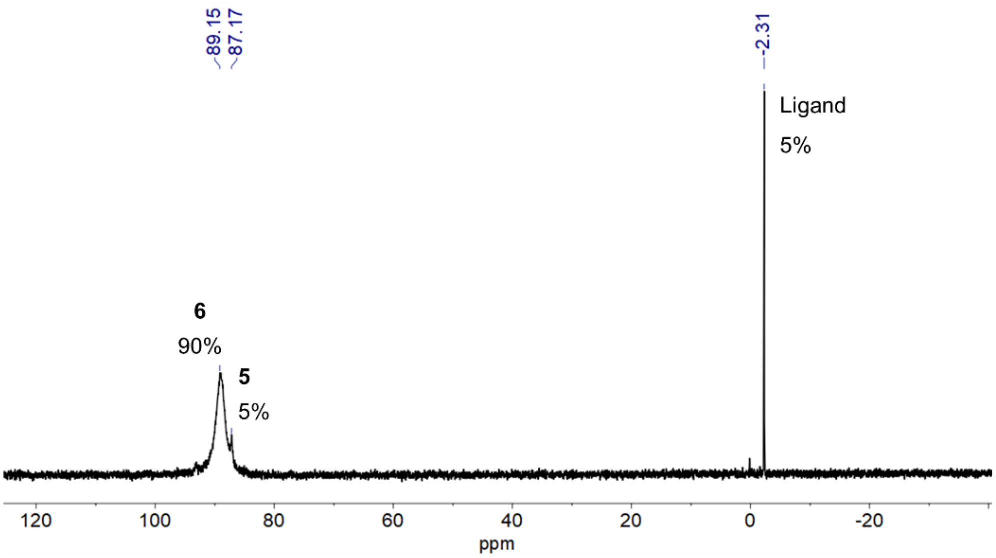


**Figure S109** $^{31}P\{^1H\}$ NMR (162 MHz, $d_8$-toluene) spectrum of complex 6, showing ca. 5% 5, and a small amount of decomposition (5%) indicated by the presence of free ligand.

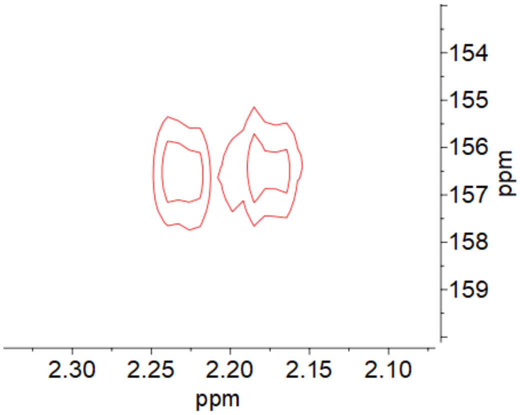


**Figure S110** $^1H$-$^{13}C$ HMBC ($d_8$-toluene, 500 MHz – 126 MHz) NMR partial spectrum of complex 6 showing correlation between N-H/C-H activated formanilide and backbone C-H protons.

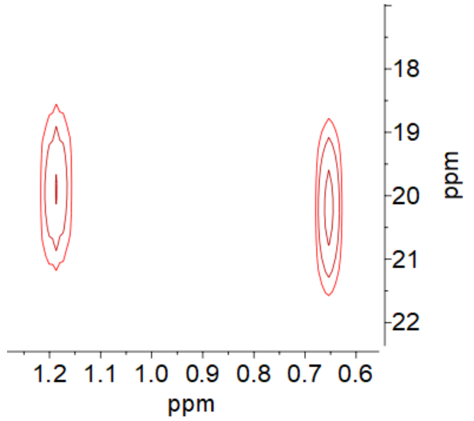


**Figure S111** $^{1}H$-$^{13}C$ HSQC ($d_8$-toluene, 500 MHz-126 MHz) partial spectrum of complex 6 showing correlation between isopropyl Me and signals at 20 ppm (under residual toluene signal).

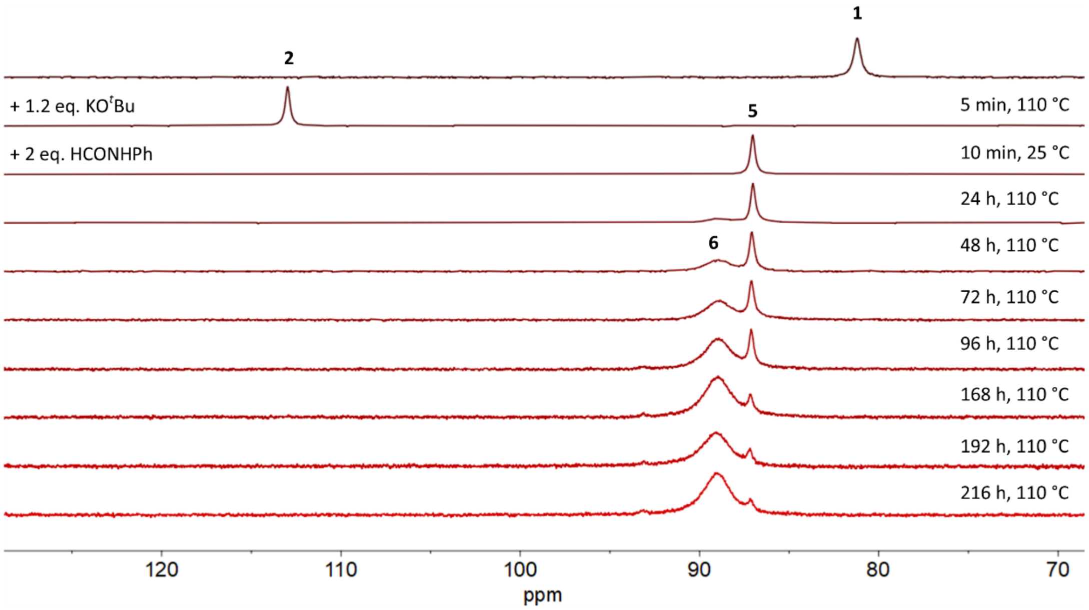


**Figure S112** $^{31}P\{^{1}H\}$ NMR (162 MHz, $d_8$-toluene) spectra of reaction progression for the generation of complex 6.

## 14.6 NMR spectra from *in situ* speciation

Using **DF-1** and **DA-1** with precatalyst **1** (4 mol%) and KO$^t$Bu (8 mol%), under polymerisation conditions (anisole solvent, 170 °C). The reaction was heated for 1 hour and the resulting $^{31}P\{^1H\}$ NMR obtained. After 6 hours, the major $^{31}P$ containing species observed was free ligand, suggesting catalytic decomposition.

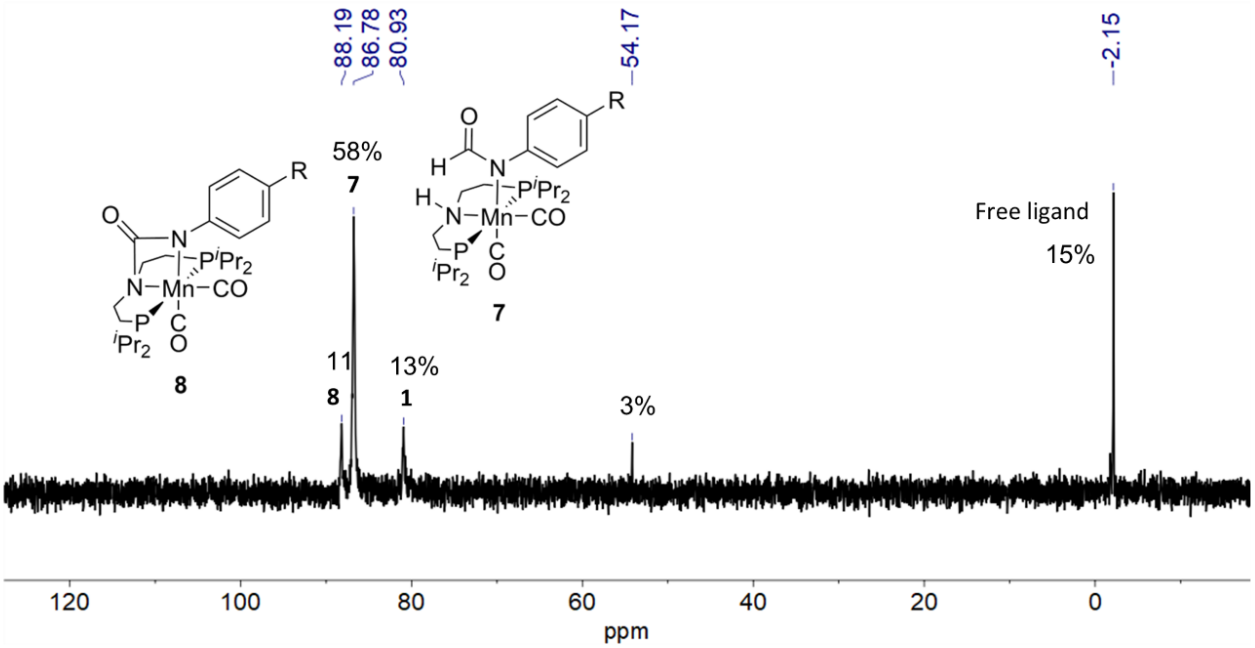


**Figure S113** $^{31}P\{^1H\}$ NMR (202 MHz, anisole) spectra obtained of the catalytic reaction mixture with proposed structures for complexes **7** and **8**, based on stochiometric experiments.

## 14.7. GC-MS selected chromatograms and mass spectra

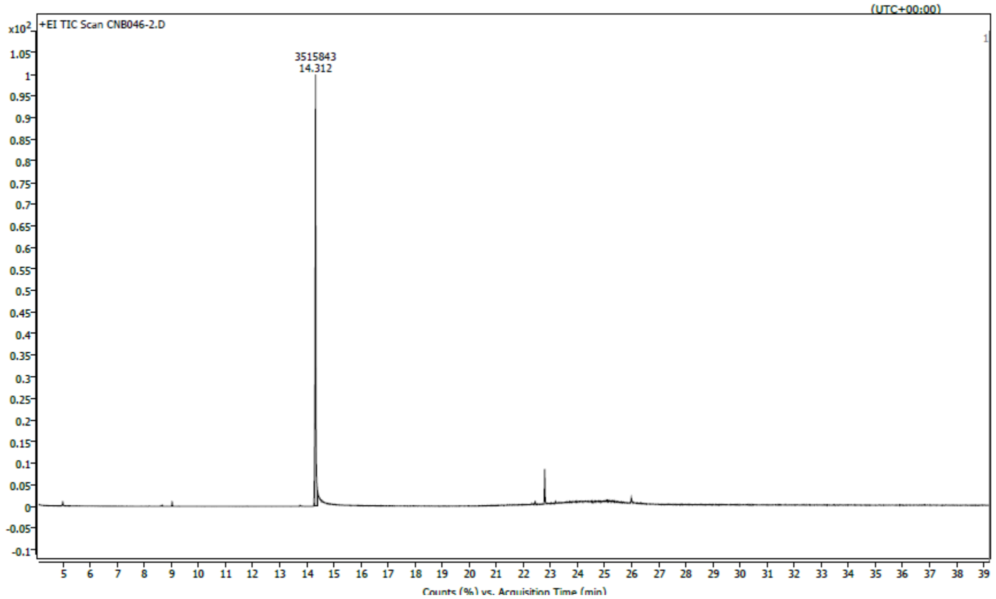


**Figure S114** Gas chromatogram of reaction of **1** with formanilide at room temperature.

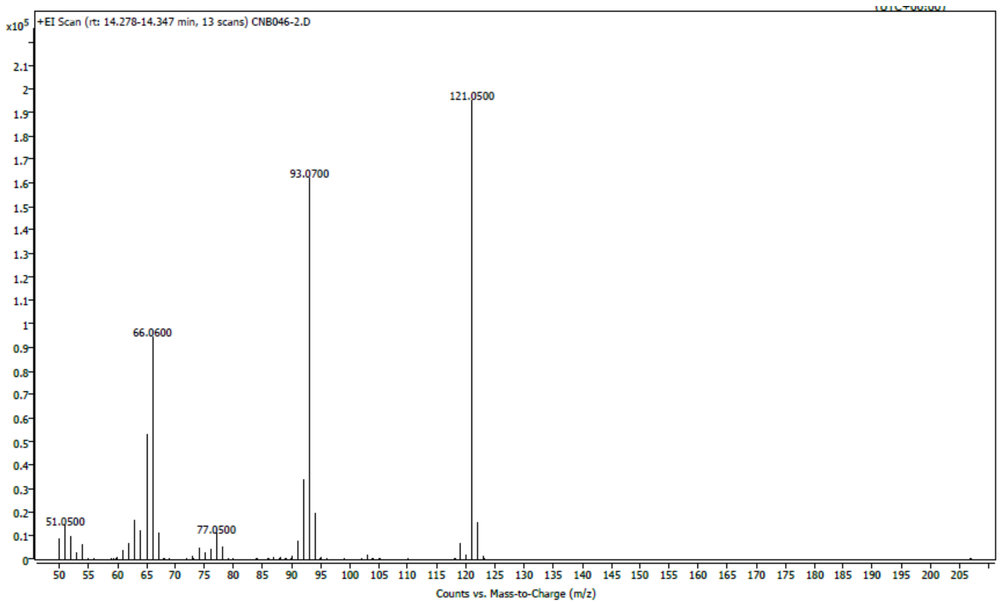


**Figure S115** Mass spectrum corresponding to formanilide observed in the GC of reaction of **1** with formanilide at room temperature.

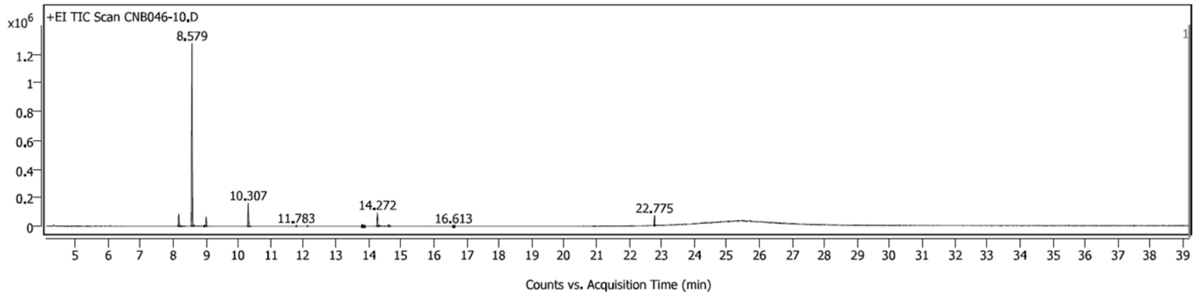


**Figure S116** Gas chromatogram of reaction of **1** with formanilide post-heating.

## 14.8 Mass Spectra

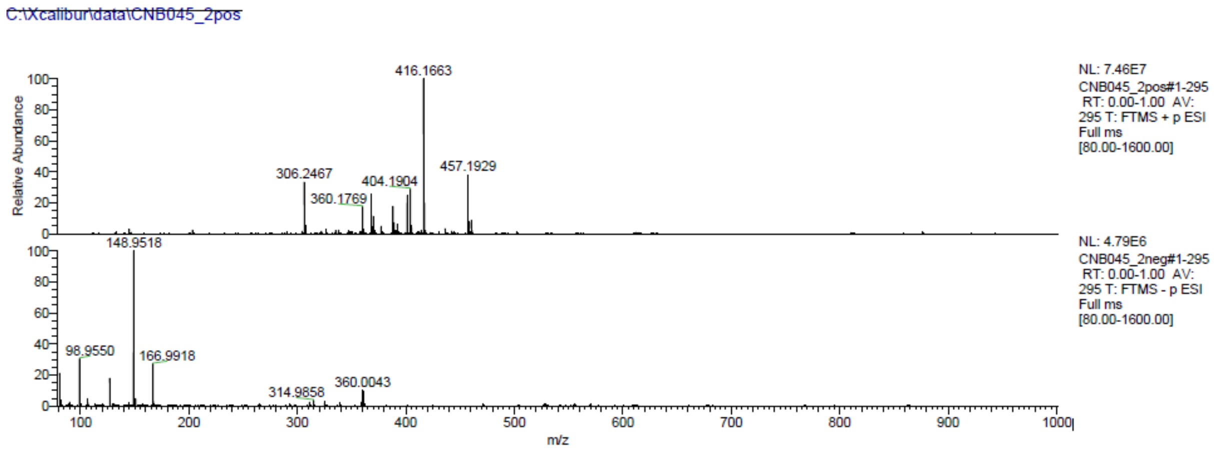


**Figure S117** HRMS (ESI, MeCN) spectrum of complex **3**.

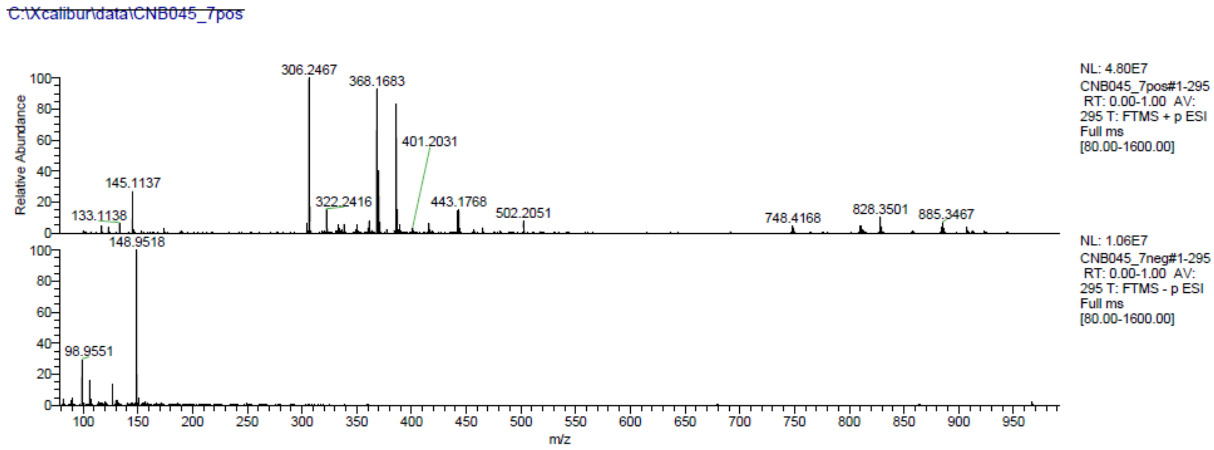


**Figure S118** HRMS (ESI, MeCN) spectrum of complex **4**.

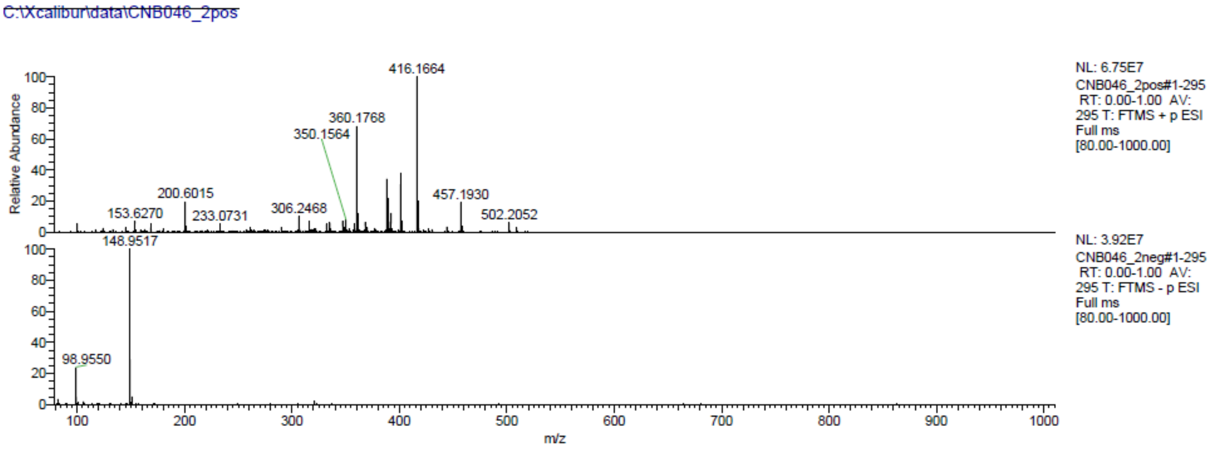


**Figure S119** HRMS (ESI, MeCN) spectrum of complex **5**.

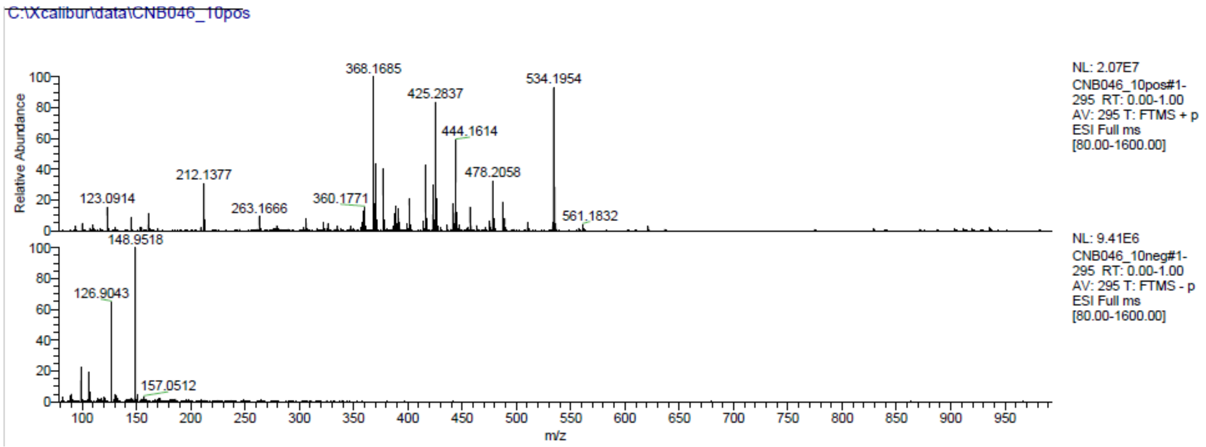


**Figure S120** HRMS (ESI, MeCN) spectrum of complex **6**.

## 15. References


1. D. H. Nguyen , X. Trivelli , F. Capet , J.-F. Paul , F. Dumeignil and R. M. Gauvin, *ACS Catal.*, 2017, **7** , 2022-2032.
2. K. P. Dhake, P. J. Tambade, R. S. Singhal, B. M. Bhanage, *Green Chem. Lett. Rev.* 2011, **4**, 151.
